# *Sidastrum paniculatum* (L.) Fryxell (Malvaceae): A Promising Source of Bioactive Sulfated Flavonoids Against *Aedes aegypti* L

**DOI:** 10.3389/fphar.2021.760156

**Published:** 2022-01-07

**Authors:** Sany D. G. Marques, Diégina A. Fernandes, Yanna C. F. Teles, Renata. P. B. Menezes, Mayara S. Maia, Marcus T. Scotti, Maria F. Agra, Tania M. S. Silva, Maria de Fátima Vanderlei de Souza

**Affiliations:** ^1^ Post Graduation Program in Bioactive Natural and Synthetic Products, Federal University of Paraíba, João Pessoa, Brazil; ^2^ Laboratory of Phytochemistry Prof. Dr. Raimundo Braz Filho, Department of Pharmaceutical Sciences, Health Sciences Center, Federal University of Paraíba, João Pessoa, Brazil; ^3^ Department of Chemistry and Physics, Agrarian Sciences Center, Federal University of Paraíba, Areia, Brazil; ^4^ Department of Chemistry, Exact and Nature Sciences Center, Federal University of Paraíba, João Pessoa, Brazil; ^5^ Deparment of Biotechnology, Biotechnology Center, Federal University of Paraíba, João Pessoa, Brazil; ^6^ Department of Molecular Sciences, Rural Federal University of Pernambuco, Recife, Brazil

**Keywords:** sulfated flavonoids, virtual screening, neglected tropical diseases, *Aedes aegypti* L, *Sidastrum paniculatum* (L.) Fryxell

## Abstract

*Aedes aegypti* L. is known as the most relevant vector mosquito for viruses such as yellow fever, chikungunya, dengue, and Zika, especially in places with unplanned urbanization, and erratic water supply. Plants used in folk medicine have become a useful source of active compounds with the potential to control the dissemination of *Ae. aegypti.* Compounds isolated from Malvaceae *sensu lato* have been previously reported as larvicides, repellents, and insecticides. Recent studies have demonstrated the anti *Ae. aegypti* activity of sulfated flavonoids, an uncommon type of flavonoid derivatives*.* This research reports the phytochemical investigation of *Sidastrum paniculatum* (L.) Fryxell, a Malvaceae species with the potential against *Ae. aegypti.* Chromatographic procedures resulted in the isolation of the compounds: stearic acid (**1**), N-*trans*-feruloyltyramine (**2**), acacetin (**3**), apigenin (**4**), tiliroside (**5**), along with the sulfated flavonoids: wissadulin (**6**), 7,4′-di-*O*-methyl-8-*O*-sulfate flavone (**7**), yannin (**8**), beltraonin (**9a**), 7-*O*-sulfate isoscutellarein (paniculatumin) (**9b**), and condadin (**10**). This is the first report of compound 7-*O*-sulfate isoscutellarein (**9b**). The structures were elucidated by spectroscopic analysis (NMR, LC-HRMS and FT-IR). The sulfated flavonoids identified were submitted to a ligand-based and structure-based virtual screening against two targets: 1YIY (from adult *Ae. aegypti*) and 1PZ4 (from *Ae. aegypti* larvae). The results indicated that when the *O*-sulfate group is bearing the position 7, the structures are potentially active in 1PZ4 protein. On the other hand, flavonoids with the *O*-sulfate group bearing position 8 were showed to be more likely to bind to the 1YIY protein. Our findings indicated that *S. paniculatum* is a promising source of sulfated flavonoids with potential against *Ae. aegypti.*

## 1 Introduction

Mosquito-transmitted diseases are a great threat to billions of people in all tropical regions of the globe. Considering the global warming, researches have projected the increasing risk of mosquito-transmitted diseases in a warmer and more populated planet (Colon-González et al., 2021). Accordingly, it is crucial to increase investments in the development of treatments, vaccines and alternative tools to combat these viruses, including sustained ways to control the virus’s vector, especially in places with unplanned urbanization, and poor infrastructures and erratic water supply (Iwamura et al., 2020).

The mosquito *Aedes aegypti* L. is known as the most relevant vector for viruses such as yellow fever, chikungunya, dengue, and Zika (Rodrigues et al., 2021). In spite of that, it is still considered a great challenge to achieve the control of *Ae. aegypti* dissemination in poor and developing countries (Quintero et al., 2017). In this context, plants used in folk medicine have become a promising tool to be used to avoid the dissemination of *Ae. aegypti* mosquitoes (Silvério et al., 2020)*.*

The use of natural products presents the great advantage of being considered a safe alternative since they are biodegradable into nontoxic compounds, preventing the contamination of the environment with chemicals (Silvério et al., 2020). Extracts and compounds isolated from Malvaceae *sensu lato* species, such as *Sida acuta, Abutilon indicum, Waltheria viscosissima,* and *Helicteres velutina*, have been previously reported as larvicides, repellents and insecticides against *Ae. aegypti*. Sulfated flavonoids with anti-*Ae. aegypti* potential have been recently reported from *H. velutina* and *W. viscosissima* (Fernandes et al., 2018, 2019, 2020; Leite-Ferreira, et al., 2019).

Species from *Sidastrum* genus are traditionally used in Brazilian northeast to treat inflammatory diseases, cough, bronchitis, and insect bites (Teles Y. C. F. et al., 2015; Gomes et al., 2015). *Sidastrum paniculatum* (L.) Fryxell is a Malvaceae species known in Brazil as “malva roxa” and “malvavisco”. Previous studies on *S. paniculatum* showed its antileishmanial activity and led to the isolation of steroids, phaeophytins, terpenoids, and phenolic compounds (Teles Y. C. F. et al., 2015). Based on the diversity of compounds reported from Malvaceae species, the knowledge on *S. paniculatum* metabolites is still poor.

Considering that, this study presents a phytochemical investigation on *S. paniculatum*, aiming to contribute to the chemotaxonomy of Malvaceae *sensu lato* family. In addition to that, we performed a virtual screening to evaluate the activity of the identified sulfated flavonoids by analyzing their interactions with *Ae. aegypti* molecular targets.

## 2 Materials and Methods

### 2.1 General Procedures and Chemicals

Chromatographic glass columns were used for purification of the compounds, packed with Silica gel 60 (ASTM, 230–400 mesh, Macherey Nagel®), Sephadex LH-20 (Sigma-Aldrich®) or Amberlite XAD-2 (Sulpeco®) as stationary phases. Thin layer chromatography (TLC) was carried out using Silica gel plates (Whatman®). The isolated compounds were identified by Infrared (IR), Perkin-Elmer apparatus, FT-IR-1750 and Shimadzu—Prestige 21 models using KBr disc; and 1D and 2D NMR (^1^H 500 MHz, ^13^C 125 MHz, and ^1^H 200 MHz, ^13^C 50 MHz—Varian System), using deuterated solvents: Methanol-*d*_*4*_ (CD_3_OD) or chloroform (CDCl_3_). The chemical shifts were expressed as parts per million (ppm) and the coupling constants (*J*) in Hz.

LC-HRMS (Accela 600 HPLC system combined with an Exactive (Orbitrap)—Thermo Fisher Scientific (Bremen, Germany) was used to obtain the high-resolution mass spectra in negative or positive mode. The samples were solubilized in methanol (HPLC grade) to obtain a concentration of 1 mg/ml. The injection volume was 20 µl. The column used was a reverse phase ACE C-18 (150 × 3 mm, 3 µm) from HiChrom (Reading, United States). The mobile phase gradient was a mixture of 0.1% formic acid in H_2_O (solvent A) and acetonitrile (solvent B). The flow rate was 300 μl/min. The obtained results were analyzed using Xcalibur 2.2 (Thermo Fisher Scientific) (Bremen, Germany) (Fernandes et al., 2018).

### 2.2 Plant Material

The botanical material (aerial parts) of *S. paniculatum*, were collected in Pedra da Boca Park, located in Araruna City, Paraiba/Brazil, in June 2008 (SISBIO Authorization Number 46923-2). The plant was identified by Prof. Dr. Maria de Fátima Agra, and a voucher specimen (JPB-6051) was authenticated and deposited at Lauro Pires Xavier Herbarium (CCEN/UFPB). This research has been registered at National System of Genetic Resource Management and Associated Traditional Knowledge (SisGen—A568B8A).

### 2.3 Extraction and Isolation of Compounds

The plant material was dried in an oven in a temperature of 40°C for 72 h. After that, it was grounded in a mechanical mill, yielding 3.7 kg of a powder which was submitted to maceration with ethanol for three consecutive days. This process was repeated in order to maximum extraction of chemical constituents. The obtained ethanol extractive solution was concentrated in a rotatory evaporator, resulting 250 g of crude ethanol extract (CEE). The CEE was solvent-extracted with *n*-hexane (Hex), chloroform (CHCl_3_), ethyl acetate (AcOEt) and *n*-butanol, resulting in their respective phases, and hydroalcoholic phase.

The precipitate from chloroform phase (2.0 g) was submitted to chromatographic column using Sephadex LH-20 and eluted with MeOH and MeOH:CHCl_3_ (1:1), resulting in 30 fractions. The obtained fractions were combined after thin layer chromatography (TLC) analyses. Fraction 7–25 (1.03 g) was submitted to chromatographic column (CC) using Silica gel eluted with hexane, AcOEt and MeOH in increasing polarity mixtures. Fraction 17–26 (100 mg) was again submitted to a similar procedure, resulting 24 fractions. The fraction 9 (amorphous solid) was found to be pure, named as compound **1** (5.0 mg) and fraction 16 (green amorphous solid) named as compound **2** (15.0 mg).

N-butanol phase (5.0 g) was submitted to CC using Amberlite XAD-2, eluted with H_2_O, H_2_O:MeOH (1:1), Acetone, AcOEt, and Hex. Acetone fraction (300.0 mg) was submitted to Sephadex (LH-20), as the mobile phase, MeOH, and MeOH:CHCl_3_ (7:3). After TLC, the fractions were combined and selected to new Sephadex CC. The fraction 3–5 (15.0 mg) was submitted to Silica Flash CC and eluted with hexane, AcOEt and MeOH, the fraction 2–3 (yellow crystal) was pure, named as compound **3** (5.0 mg).

The fraction H_2_O:MeOH (1:1) (2.0 g) was chromatographed in successive Sephadex CC, using as the mobile phase MeOH and MeOH: CHCl_3_ (1:1). This procedure provided compounds **7** (15.0 mg), **8** (15.0 mg), **9a,** and **9b** (9.0 mg) as yellow amorphous solids.

Methanol fraction (800 mg) was submitted to Sephadex CC eluted with MeOH and MeOH: CHCl_3_ (1:1). The fractions 14–20 (50.0 mg) were submitted to Silica gel CC using Hex, AcOEt, and MeOH, which led to isolation of the compounds **4** (6.0 mg) and **5** (10.0 mg) as yellow amorphous solids. The fraction 26–28 (20.0 mg) was submitted the same method previously described, resulting in the isolation of the compound **6** (7.0 mg), a yellow amorphous solid.

Hydroalcoholic phase (5.0 g) was chromatographed using Amberlite XAD-2 CC, eluted with H_2_O, H_2_O:MeOH, acetone, AcOEt, and hexane. Methanol fraction (1.0 g) was submitted to Silica gel CC twice, resulting in 10 fractions. These fractions were analyzed by TLC and combined. The fraction 9 (50.0 mg) was submitted to a similar chromatography. From this procedure, the fraction 10 showed one spot at TLC and it was named as compound **10** (10.0 mg).

### 2.4 Computational Study

#### 2.4.1 Dataset

From the ChEMBL database, we select a set of chemical structures for the construction of a predictive model. The set was composed by 162 diverse chemical structures that have been studied and inhibited larvae of *Ae. aegypti*.

The compounds were classified using values of −logIC_50_ (mol/L) = pIC_50,_ which led us to assign 85 active (pIC_50_ ≥ 4.15) and 76 inactive compounds (pIC_50_ < 4.15). In this case, the IC_50_ represented the concentration required for 50% inhibition of larvae of *Aedes aegypti.* Through a bibliographical search, 11 flavonoids (Gikonyo et al., 1998; Rajkumar and Jebanesan, 2008; Perumalsamy et al., 2015; Guarda et al., 2016) have been added to this bank against *Aedes aegypti* larvae, with a total of 173 molecules. Another dataset of molecules isolated from *S. paniculatum* was built from the phytochemical study of this species. For all structures, SMILES codes were used as input data in a Marvin 18.10, 2018, ChemAxon (http://www.chemaxon.com) (ChemAxon, 2018). We used (Standardizer software 2018) [JChem 18.10, 2018; ChemAxon (http://www.chemaxon.com)] to canonize structures, add hydrogens, perform aromatic form conversions, clean the molecular graph in three dimensions, and save compounds in *sdf*. format. Three dimensions structures (3D) were used as input data in the software Dragon 7.0 to generate descriptors (Talete, 2016) where it is possible to predict the biological and physical-chemical properties of the molecules. This calculus was realized for the set of chemical structures with knowledge activity against *Ae. aegypti* larvae*.*

#### 2.4.2 Prediction Model

The Dragon 7.0 software (Talete, 2016) generated 1,232 descriptors for 173 molecules of known activity against *Ae. aegypti* larvae. These descriptors were used as input date no Knime software for generation the predictive model. The Dragon 7.0 software can calculate 5,270 molecular descriptors, covering several approaches. These descriptors are organized in 30 logical blocks. The descriptors list includes the simplest types of atoms, functional groups and fragment counting, topological and geometric descriptors, tridimensional descriptors, but also several of them estimates properties such as logP, and Lipinski (Mauri et al., 2006).

The Knime 3.4.0 software (Knime 3.4.0 the Konstanz Information Miner Copyright, 2003–2014, www.knime.org) (Berthold et al., 2006) was used to perform the analyzes and created the model *in silico*. The descriptors and class variables were imported from the software Dragon 7.0, and for each one the data were divided using the “Partitioning” node with the “stratified sample” option to create a training set and a test set, and encompassing 80 and 20% of the compounds, respectively.

Although the compounds were selected randomly, the same proportion of active and inactive samples was maintained in set. For internal validation, we employed cross-validation using 10 randomly selected, stratified groups, and the distributions according to activity class variables were found to be maintained in all validation groups and in the training set.

Descriptors were selected, and a model was generated using the training set and the Random Forest algorithm (RF) (Salzberg, 1994), using the WEKA nodes (Hall et al., 2009). The parameters selected for RF included the following settings: number of trees to build = 50, seed for random number generator = 1, for the model. The internal and external performances of the selected models were analyzed for sensitivity (true positive rate, i.e., active rate), specificity (true negative rate, i.e., inactive rate), and accuracy (overall predictability).

Additionally, the sensitivity and specificity of the Receiver Operating Characteristic (ROC) curve was find to describe true performance with more clarity than accuracy. It was also used the domain of applicability based on Euclidean distances in order to signal compounds in the test set for which the predictions may be unreliable. Similarity measurements are used to define the applicability domain of the model based on Euclidean distances between all training, test and virtual screening compounds.

The distance from a compound of a test compound to its nearest neighbor in the training set is compared to the predefined applicability domain limit, if the similarity is beyond this limit, the prediction is considered unreliable (Zhang et al., 2006).

#### 2.4.3 Molecular Docking

The target proteins of *Ae. aegypti* 1YIY (Han et al., 2005), 1PZ4 (Dyer et al., 2003), with their respective inhibitor ligands were downloaded from Protein Data Bank (http://www.rcsb.org/pdb/home/home.do), details of each enzyme can be seen in Table 1.

**TABLE 1:** Information about the target proteins of *Aedes aegypti* and their respective ligand.

Protein ID	Classification	Ligand	Localization
*Aedes aegypti* kynurenine aminotransferase (1YIY)	Transferase	4′-Deoxy-4′-Aminopyridoxal-5′-Phosphate Pyridoxamine-5′-Phosphate	Adult mosquito head
Sterol Carrier Protein-2 (1PZ4)	Lipid binding	Palmitic acid	Large intestine of larvae

The sulfated flavonoids isolated from *S. paniculatum* were submitted to molecular docking using the Molegro Virtual Docker, v. 6.0.1 (MVD). All the water compounds were deleted from the enzyme structure, and the enzyme and compound structures were prepared using the default parameter settings in the software package (ligand evaluation: Internal ES, Internal H-Bond, Sp2-Sp2 Torsions, all checked; number of runs: 10 runs; algorithm: MolDock SE; maximum interactions: 1,500; Max. population size: 50; Max. steps: 300; neighbor distance factor: 1.00; Max. number of poses returned: 5).

The docking procedure was performed using a GRID of 15 Å in radius and 0.30 in resolution to cover the ligand-biding site of the enzyme’s structures. Template docking was used to focus the search on poses similar to the ligand interactions and conformation. The Moldock score algorithm was used as the score function (Thomsen and Christensen, 2006). The energy of the crystallized ligand of each protein is calculated automatically by the Moldock Score. Based on the pose of the crystallized ligand, the Moldock score algorithm automatically converts the ligand’s energy to the energy scale used by it.

#### 2.4.4 Molecular Dynamics

Molecular dynamics simulations were performed to estimate the stability of interactions between proteins and ligands using Gromacs 5.0 software (Berendsen et al., 1995; Abraham et al., 2015). The topology of the ligands was prepared using the PRODRG topology generator (http://davapc1.bioch.dundee.ac.uk/cgi-bin/prodrg/submit.html) (Schuttelkopf and Aalten, 2004) applying the GROMOS43a1 force field. The protein topology was also prepared using the GROMOS43a1 force field. The molecular dynamics simulation was performed using the SPC water model of point load extended in a dodecahedral box (Bondi, 1964). The system was neutralized by adding ions (Cl^−^ and Na^+^) and minimized to remove bad contacts between complex molecules and solvent. The system was also balanced at 300 K using the V-rescale algorithm at 100 ps as NVT set (constant number of particles, volume, and temperature) followed by equilibrium at 1 atm of pressure using the Parrinello-Rahman algorithm as set NPT (constant number particles, pressure and temperature) to 100 ps. The DM simulations were performed in 50,000 cycles at 1 ns. In order to determine the stability of the structure and if the complex is stable near the experimental framework, the mean square root displacement (RMSD) of all heavy atoms was calculated relative to the starting structures. Residual fluctuations (RMSF) were also analyzed to understand the role of residues near the receptor binding site. To verify the interaction energy of the protein-ligand complex, we calculated the short-range Coulombic interaction energy and the Lennard-Jones short-range energy for 10 ns. The RMSD and RMSF plots were generated in the Grace (http://plasma-gate.weizmann.ac.il/Grace/) software and the protein and ligands were visualized at UCSF Chimera (Pettersen et al., 2004).

## 3 Results

### 3.1 Phytochemical Study

**FIGURE 1:**
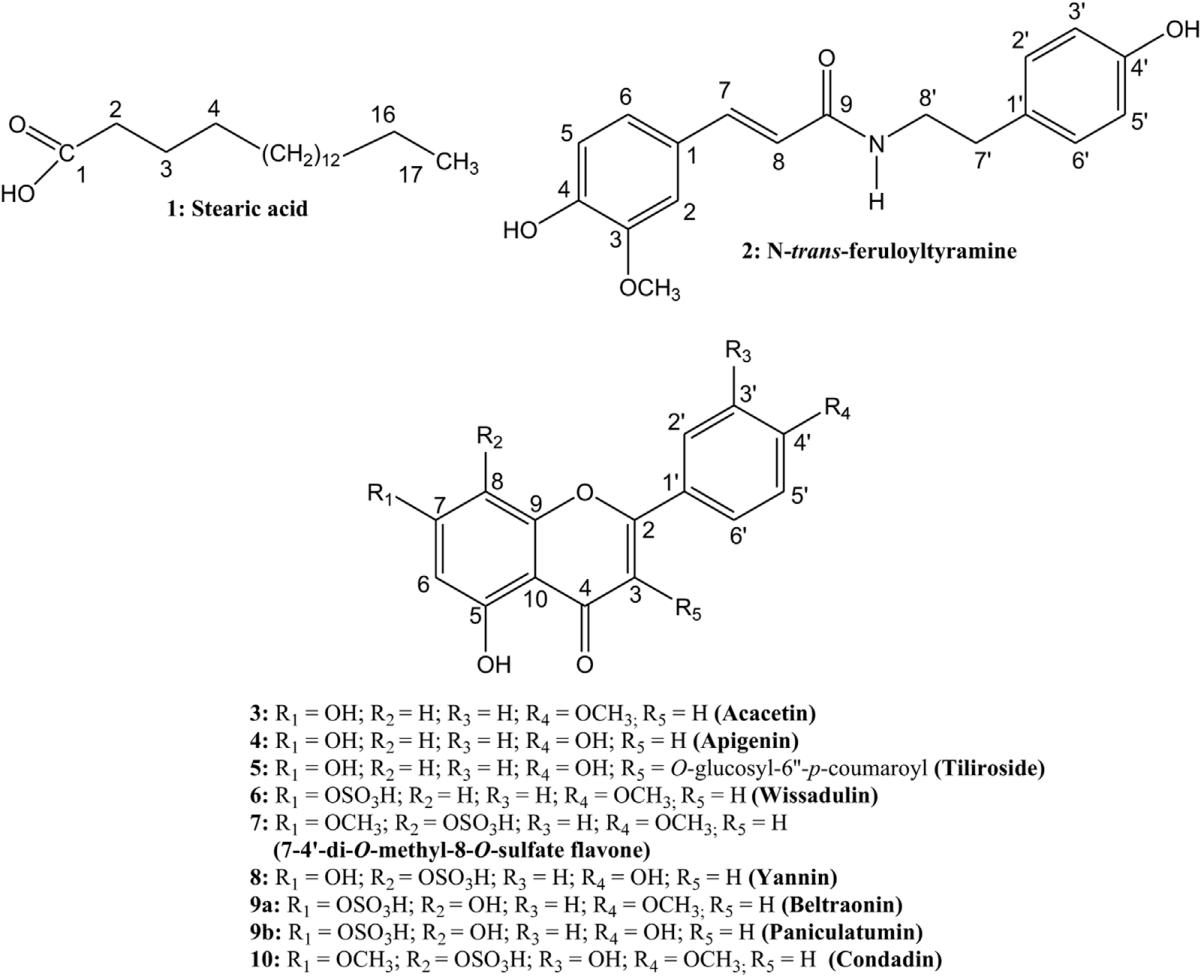
Compounds isolated from *Sidastrum paniculatum* (L.) Fryxell.

The compounds identified from aerial parts of *S. paniculatum* are shown in Figure 1. The respective spectra are available as Supplementary Material.

The IR spectra of sample **9** recorded bands at 3,466 cm^−1^, characteristic for hydroxyl axial deformation. It could be seen that there were absorptions at 1,606, 1,512, and 1,362 cm^−1^, indicating a C=C of aromatic compounds, as well as at 1,654 cm^−1^, suggestive of C=O of conjugated, and bridge ketones present in flavonoids. The absorbances of asymmetric stretches at 1,362 cm^−1^ and symmetrical stretches at 1,060 cm^−1^ indicated the occurrence of an S=O group and together with absorptions at 1,251 to 966 cm^−1^, assigned to an S-O bond, and pointed to the possible presence of a sulfate group in the structure.

The ^1^H-NMR spectra showed a complex set of signals in the *δ*_H_ 6–8 ppm range (aromatic region), with different intensities suggesting that **9** might be a mixture of two compounds. The signals of *δ*_H_ 8.21 (d, 2H, *J* 9.0 Hz) coupled with *δ*_H_ 7.08 (d, 2H, *J* 9.0 Hz) and 8.12 (d, 2H, *J* 9.0 Hz) with 6.92 (d, 2H, *J* 9.0 Hz) suggested two AA’BB’ systems. The first system was indicative of a methoxyl substituent in C-4′, deshielding the 3′5′ and 2′6′ positions. The second system proposed the presence of an OH-4′ group, which protects the H-3′5′ and H-2′, 6’. The substituents were later confirmed by 2D NMR analysis, evidence the presence of a mixture of two flavones, and renamed **9a** and **9b**, respectively. A singlet at *δ*_H_ 3.89 was attributed to the methoxyl group of C-4′ of an AA’BB’ system, later confirmed by 2D NMR.

The APT ^13^C-NMR spectra revealed weak peaks and aligned with HMBC, HMQC, and COSY spectra, thereby allowing identification of the substances, and confirming the position of the substituents. The major constituent **9a** showed singlets at *δ*_H_ 6.74 attached to δc 104.2 (C-3) and at *δ*_H_ 7.17 attached to *δ*_C_ 104.4 (C-6) (HMQC). The HMBC showed a strong correlation of the methoxyl with a carbon at *δ*_C_ 164.6, confirming the methoxyl to be at C-4′ and indicated a *para*-substituted B ring with a scaffold similar to 4′-*O*-methylisoscutellarein.

However, comparisons with 4′-*O*-methylisoscutellarein ^13^C-NMR and ^1^H-NMR data showed that for compound **9a** the signals for C-7 is shielded while C-6 and H-6 is deshielded. Besides, sample **9** was found to be more polar than 4′-*O*-methylisoscutellarein at TLC. These facts suggest an *O*-sulfate group attached at C-7 of compound **9a**. These effects in chemical shifts are usually observed for *O*-sulfate flavonoids (Teles et al., 2015b; Teles et al., 2018). The presence of *O*-sulfate group was confirmed by HRMS. The accurate mass for compound 9a (major compound) as an [M-H]- ion, was *m/z* 379.0120 (C_16_H_12_O_9_S).

The minor constituent **9b** showed singlets at *δ*_H_ 6.69 attached to C-3 (*δ*_C_ 103.7) and *δ*_H_ 7.16 attached to C-6 (*δ*_C_ 104.4) (Table 2). Comparison of the NMR data of **9b** and isoscutellarein indicated that positions 7 was more shielded and position C-6 and H-6 was found to be deshielded, like compound **9a**, indicating the presence of the *O*-sulfate group at C-7. Comparing **9b** ^13^C-NMR spectral data with ^13^C-NMR data of **8**, was observed that **9b** had C-7 more shielded (∼3 ppm) and positions C-6 and C-8 more deshielded, corroborating with the indication of the *O*-sulfate group at C-7. The The compound **9a** was identified as 4′-*O*-methyl-7-*O*-sulfate isoscutellarein (beltraonin) (Teles et al., 2015b) and compound **9b** as 7-*O*-sulfate isoscutellarein (paniculatumin) (Figure 1). The compound **9b** is being reported for the first time in the literature.

**TABLE 2:** NMR data for compounds Beltraonin (**9a)** and Paniculatumin **(9b)** (500 and 125 MHz, CD_3_OD).

Position	9a			9b		
	*δ*_C_ ppm	*δ*_H_ ppm	HMBC	*δ*_C_ ppm	*δ*_H_ ppm	HMBC
		(*J* = Hz)	^*2*^ *J/* ^*3*^ *J*		(*J* = Hz)	^*2*^ *J/* ^*3*^ *J*
2	166.9	—		167.1	—	
3	104.2	6.74 (s, 1H)	C-2, C-4/C-1′, C-10	103.7	6.69 (s, 1H)	C-2, C-4/C-1′, C-10
4	184.4	—		184.4	—	
5	158.4	—		158.4	—	
6	104.4	7.17 (s, 1H)	C-5, C-7/C-8, C-10	104.4	7.16 (s, 1H)	C-5, C-7/C-8, C-10
7	153.5	—		153.5	—	
8	125.6	—		125.6	—	
9	151.5	—		151.5	—	
10	108.1	—		108.2	—	
1′	124.5	—		123.6	—	
2′	130.3	8.21 (d, *J* 9.0)	C-4′, C-6′	130.4	8.12 (d, *J* 9.0)	C-4′, C-6′
3′	115.5	7.08 (d, *J* 9.0)	C-4’/C-1′, C-5′	116.9	6.92 (d, *J* 9.0)	C-4’/C-1′, C-5′
4′	164.6	—		163.0	—	
5′	115.5	7.08 (d, *J* 9.0)	C-4’/C-1′, C-3′	116.9	6.92 (d, *J* 9.0)	C-4’/C-1′, C-3′
6′	130.3	8.21 (d, *J* 9.0)	C-4′, C-2′, C-2	130.4	8.12 (d, *J* 9.0)	C-4′, C-2′, C-2
OCH_3_-4′	56.0	3.89 (s)	C-4′	—	—	

Stearic acid (**1**): IR (ν, KBr, cm^−1^) 3,459, 1,642, 1,093, 471; ^1^H NMR (500 MHz, CDCl_3_, *δ*) 2.23 (t, 2H, *J* 7.5 Hz, CH_2_), 1.64 (m, 2H, CH_2_), 1.29–1.38 (m, 26H, CH_2_), 2.02 (dq, 2H, CH_2_), 0.89 (t, 3H, *J* 7.0 Hz, CH_3_); ^13^C NMR (125 MHz, CDCl_3_, *δ*) 175.3 (C-1), 31.9 (C-2), 29.6 (C-3/C-10), 31.8 (C-11), 22.7 (C-16), 14.1 (C-17). The ^1^H- and ^13^C-NMR spectral data are consistent with published data (Bang et al., 2002).

N-*trans*-feruloyltyramine (**2**): ^1^H NMR (200 MHz, CD_3_OD, *δ*) 7.43 (d, 1H, *J* 15.8 Hz, CH), 7.10 (d, 2H, *J* 8.0 Hz, 2CH), 7.04 (brd, 1H, *J* 8.0 Hz, CH), 7.03 (bs, 1H, CH), 6.79 (d, 1H, *J* 8.0 Hz, CH), 6.71 (d, 2H, *J* 8.4 Hz, 2CH), 6.4 (d, 1H, *J* 15.8 Hz, CH), 3.87 (s, 3H, OCH_3_), 3.46 (t, 2H, *J* 6.9 Hz, CH_2_), 2.74 (t, 2H, *J* 6.9 Hz, CH_2_); ^13^C NMR (50 MHz, CD_3_OD, *δ*) 169.15 (C-9), 156.86 (C-4′), 149.87 (C-3), 149.22 (C-4), 142.02 (C-7), 131.26 (C-1′), 130.73 (C-2′, C-6′), 128.22 (C-1), 123.21 (C-6), 118.69 (C-8), 116.43 (C-5), 116.24 (C-3′,C-5′), 111.45 (C-2), 56.33 (3- OCH_3_), 42.54 (C-8′), 35.88 (C-7’). The ^1^H- and ^13^C-NMR spectral data are consistent with published data (Cavalcante et al., 2010).

Acacetin (**3**): ^1^H-NMR (500 MHz, CD_3_OD, *δ*) 6.64 (s, 1H, CH), 6.22 (d, 1H, *J* 2.0 Hz, CH), 6.47 (d, 1H, *J* 2.0 Hz, CH), 7.95 (d, 2H, *J* 9.0 Hz, 2CH), 7.09 (d, 2H, *J* 9.0 Hz, 2CH), 3.89 (s, 3H, OCH_3_); ^13^C-NMR (125 MHz, CD_3_OD, *δ*) 165.8 (C-2), 104.7 (C-3), 183.9 (C-4), 162.4 (C-5), 100.5 (C-6), 163.4 (C-7), 95.4 (C-8), 159.4 (C-9), 105.3 (C-10), 124.5 (C-1′), 129.5 (C-2′, C-6′), 115.8 (C-3′, C-5′), 164.3 (C-4′), 56.46 (4′-OCH_3_). The ^1^H- and ^13^C-NMR spectral data are consistent with published data (Teles et al., 2015b).

Apigenin (**4**): ^1^H-NMR (500 MHz,CD_3_OD, *δ*) 6.60 (s, 1H, CH), 6.22 (d, 1H, *J* 2.0 Hz, CH), 6.46 (d, 1H, *J* 2.0 Hz, CH), 7.85 (d, 2H, *J* 9.0 Hz, 2CH), 6.93 (d, 2H, *J* 9.0 Hz, 2CH); ^13^C-NMR (125 MHz, CD_3_OD, *δ*) 164.9 (C-2), 103.5 (C-3), 182.4 (C-4), 161.8 (C-5), 99.9 (C-6), 164.8 (C-7), 94.7 (C-8), 158.0 (C-9), 103.9 (C-10), 121.9 (C-1′), 129.5 (C-2′, C-6′), 116.6 (C-3′, C-5′), 161.3 (C-4’). The ^1^H- and ^13^C-NMR spectral data are consistent with published data (Teles et al., 2015b).

Tiliroside (**5**): ^1^H-NMR (500 MHz, CD_3_OD, *δ*) 6.13 (d, 1H, *J* 2.0 Hz, CH), 6.30 (d, 1H, *J* 2.0 Hz, CH), 8.00 (d, 2H, *J* 9.0 Hz, 2CH), 6.82 (d, 2H, *J* 9.0 Hz, 2CH), 5.23 (d, 1H, *J* 7.5 Hz, CH), 3.30–3.50 (m, 4H, 4CH), 4.18–4.30 (dq, 2H, *J* 2.0 and 11.0 Hz, *J* 6.5 and 12.0 Hz, CH_2_), 7.33 (d, 2H, *J* 9.0 Hz, 2CH), 6.80 (d, 2H, *J* 9.0 Hz, 2CH), 7.40 (d, 1H, *J* 16 Hz, CH), 6.10 (d, 1H, *J* 16 Hz, CH); ^13^C-NMR (125 MHz, CD_3_OD, *δ*) 159.4 (C-2), 135.1 (C-3), 179.3 (C-4), 162.9 (C-5), 100.4 (C-6), 167.1 (C-7), 95.3 (C-8), 158.7 (C-9), 105.3 (C-10), 122.7 (C-1′), 132.4 (C-2′, C-6′), 117.0 (C-3′, C-5′), 161.6 (C-4′), 104.2 (C-1″), 75.6 (C-2″), 78.3 (C-3″), 71.7 (C-4″), 75.6 (C-5″), 64.4 (C-6″), 127.1 (C-1‴), 130.9 (C-2‴, C-6‴), 116.3 (C-3‴, C-5‴), 161.3 (C-4‴), 146.6 (C-7‴), 114.8 (C-8‴), 168.8 (C-9‴). The ^1^H- and ^13^C-NMR spectral data are consistent with published data (Teles et al., 2015b).

Wissadulin (**6**): IR (ν, KBr, cm^−1^) 3,452, 2,925, 1,628, 1,384, 1,253, 1,183, 830; ^1^H-NMR (500 MHz, CD_3_OD, *δ*) 6.72 (s, 1H, CH), 7.96 (d, 2H, *J* 9.0 Hz, 2CH), 7.11 (d, 2H, *J* 9.0 Hz, 2CH), 3.89 (s, 3H, OCH_3_); ^13^C-NMR (125 MHz, CD_3_OD, *δ*) 166.4 (C-2), 104.2 (C-3), 106.4 (C-10), 124.6 (C-8), 129.5 (C-2′, C-6′), 115.7 (C-3′, C-5′), 164.5 (C-4′), 56.1(4′-OCH_3_); HRMS (FTMS + pESI) *m/z*, observed: 363.0165; C_16_H_12_O_8_S [M]^-^ required: 363.0180. The ^1^H- and ^13^C-NMR spectral data are consistent with published data (Teles et al., 2015b).

7.4′-di-O-metil-8-O-sulfate flavone (**7**): IR (ν, KBr, cm^−1^) 3,476, 3,092, 1,654, 1,604, 1,246, 1,065, 995; ^1^H-NMR (500 MHz, CD_3_OD, *δ*) 6.67 (s, 1H, CH), 6.52 (d, 1H, *J* 2.0 Hz, CH), 8.21 (d, 2H, *J* 9.0 Hz, 2CH), 7.08 (d, 2H, *J* 9.0 Hz, 2CH), 3.95 (s, 3H, OCH_3_), 3.89 (s, 3H, OCH_3_); ^13^C-NMR (125 MHz, CD_3_OD, *δ*) 166.6 (C-2), 103.8 (C-3), 184.3 (C-4), 159.6 (C-5), 96.9 (C-6), 160.9 (C-7), 123.4 (C-8), 151.4 (C-9), 105.5 (C-10), 124.7 (C-1′), 130.2 (C-2′, C-6′), 115.5 (C-3′, C-5′), 164.5 (C-4′), 56.0 (4′- OCH_3_), 57.0 (7- OCH_3_); HRMS (FTMS + pESI) *m/z*, observed: 393.0262; C_17_H_14_O_9_S [M]^-^ required: 393.0285. The ^1^H-and ^13^C-NMR spectral data are consistent with published data (Teles et al., 2015b).

Yannin (**8**): IR (ν, KBr, cm^−1^) 3,445, 1,655, 1,609, 1,371, 1,251, 1,051; ^1^H-NMR (500 MHz, CD_3_OD, *δ*) 6.63 (s, 1H, CH), 6.30 (d, 1H, *J* 2.0 Hz, CH), 8.04 (d, 2H, *J* 9.0 Hz, 2CH), 6.93 (d, 2H, *J* 9.0 Hz, 2CH); ^13^C-NMR (125MHz, CD_3_OD, *δ*) 166.4 (C-2), 103.3 (C-3), 183.9 (C-4), 157.7 (C-5), 100.6 (C-6), 159.2 (C-7), 122.1 (C-8), 151.5 (C-9), 105.3 (C-10), 123.1 (C-1′), 129.9 (C-2′, C-6′), 116.8 (C-3′, C-5′), 162.7 (C-4’). HRMS (FTMS + pESI) *m/z*, observed: 364.9962; C_15_H_10_O_9_S [M]^-^ required: 364.9972. The ^1^H- and ^13^C-NMR spectral data are consistent with published data (Teles et al., 2015b).

Beltraonin (**9a**): Full NMR data are presented in Table 2. The ^1^H- and ^13^C-NMR spectral data are consistent with published data (Teles et al., 2015b).

7-*O*-sulfate isoscutellarein (paniculatumin) (**9b**): Full NMR data are presented at Table 2.

Condadin (**10**): (ν, KBr, cm^−1^) 3,445, 1,655, 1,609, 1,371, 1,251, 1,051; ^1^H-NMR (500 MHz, CD_3_OD, *δ*) 6.63 (s, 1H, CH), 6.52 (s, 1H, CH), 7.66 (d, 1H, *J* 2.0 Hz), 7.08 (d, 1H, *J* 9.0 Hz), 7.75 (dd, 1H, *J* 2.0 and 9.0 Hz); ^13^C-NMR (125 MHz, CD_3_OD, *δ*) 166.7 (C-2), 103.5 (C-3), 184.0 (C-4), 159.6 (C-5), 96.7 (C-6), 160.8 (C-7), 123.0 (C-8), 151.5 (C-9), 105.1 (C-10), 124.8 (C-1′), 114.2 (C-2′), 147.9 (C-3′), 152.8 (C-4′), 112.6 (C-5′), 120.8 (C-6′), 56.8 (4′- OCH_3_), 56.2 (7- OCH_3_). HRMS (FTMS + pESI) *m/z*, observed: 409.0230 [M-H]^-^; C_17_H_14_O_10_S [M]^-^ required: 410.0308. The ^1^H- and ^13^C-NMR spectral data are consistent with published data (Fernandes et al., 2018).

#### 3.1.1 Prediction Model

Analyzing the *Ae. aegypti* model, the internal cross validation and the external test demonstrated similar statistical performance, with accuracy higher than 81%, and showing to be a model with great performance. The Table 3 summarizes the statistical rates of the RF model.

**TABLE 3:** Summary of parameters corresponding to the results obtained in all models.

	Specificity	Sensitivity	Accuracy	PPV	NPV
External Test	0.83	0.82	0.82	0.83	0.81
Internal Cross Validation	0.87	0.81	0.85	0.84	0.84

With these results it was possible to calculate the Matthews correlation coefficient (MCC) for generation evaluation of the *Ae. aegypti* model. The MCC correlations observed and predictive binary classifications, resulted in a value between −1 and +1, where +1 is a perfect prediction, and −1 indicates a complete disagreement between prediction and observation. For the external test the value obtained was 0.70 and 0.72 for the internal cross validation, revealing a good prediction, and robustness of the model.

**FIGURE 2:**
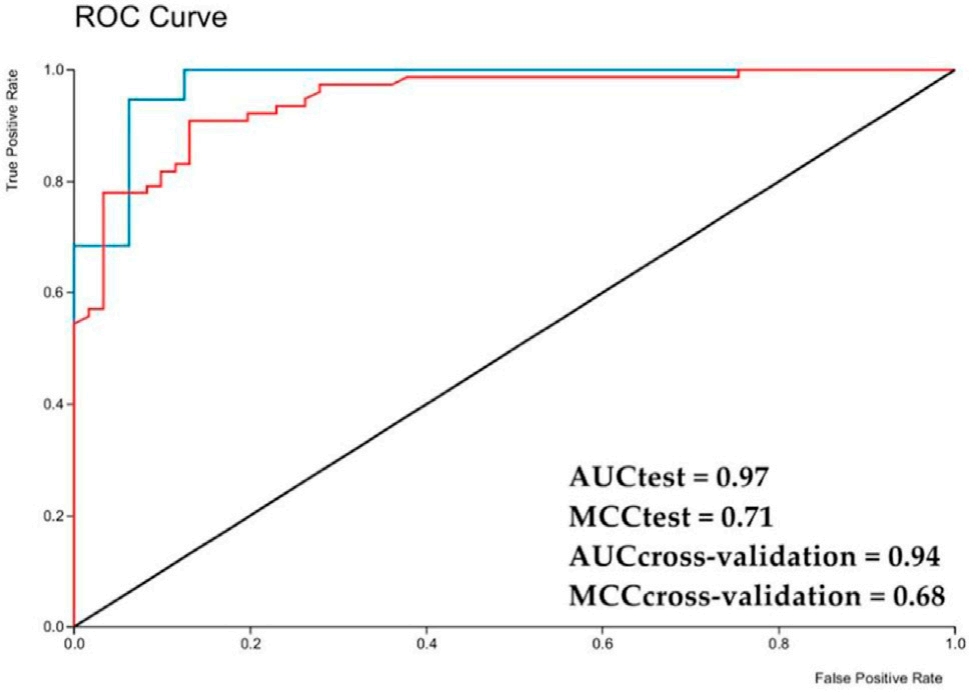
ROC chart with area under a curve for the *Aedes aegypti* model test set obtained with Random Forest. AUC—area under the curve; red line—Internal cross validation; blue line—External test.

The graphic of ROC curve that analyze the model performance was generated for the external test set and obtained an area under the curve of 0.93, and 0.91 for the internal cross validation (Figure 2). Considering that a perfect model has an area under the curve equal to 1, it is possible to state that the model can perform a high classification rate for this RF method.

**FIGURE 3:**
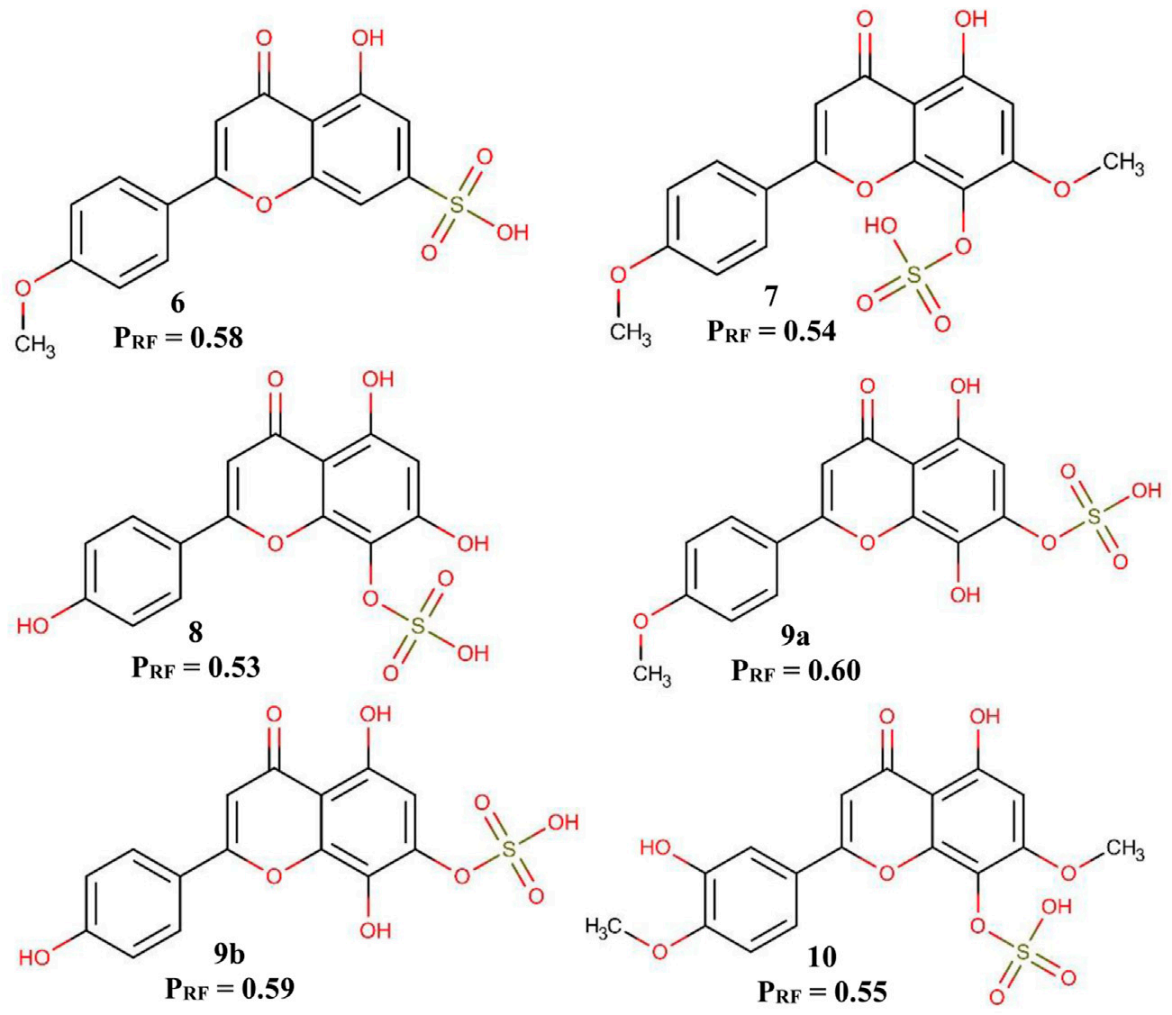
Predicted activity of flavonoids in the *Aedes aegypti* model. PRF—Probability of being active in the RF model.

The *Ae. aegypti* model was used in order to triage the sulfates flavonoids isolated from *S. paniculatum* for the identification of possible bioactive molecules against *Ae. aegypti* larvae. The six flavonoids tested showed a potential activity of more than 53% (Figure 3).

It is possible to observe in Figure 3 that the position of the sulfate group influences the probability of potentially active of the flavonoids. When the sulfate group is in position 7, the probability of active potential is greater than when it is positioned in position 8. Another fact that we can observe is that the substitution of a methoxyl in position 4′ also positively influences the probability of active potential flavonoids, a fact best observed in flavonoids 9a and 9b.

#### 3.1.2 Molecular Docking

The six sulfates flavonoids isolated from *S. paniculatum* were tested with the two *Ae. aegypti* target proteins (1YIY and 1PZ4) in order to screen the compounds with the highest potential larvicidal activity, thus a virtual screening based on the receptor structure. Then, calculations were made to obtain the molecules with the best probabilities of active potential against *Ae. aegypti* larvae. These calculations were carried out using the following formula:

Prob=EMTEM, if EMT<EL

Where, E_MT_ is the energy of the test molecule, E_M_ is the lowest energy obtained from the molecules tested and E_L_ is the energy of the ligand obtained from the crystallography of the test protein, the fraction is conditioned to the energy of the test molecule being smaller than the energy of the ligand, that is, and only molecules which have obtained binding energy lower than the binding energy of the crystallographic binder will be considered potentially active (Acevedo et al., 2018; Barros et al., 2019).

Molecular docking was validated using the redocking technique, in which the inhibitor ligand of each protein obtained the same pose after the docking was performed, and thus validating the docking performed in the study. The energy offered by Moldock is not determinant to influence the validation of the docking, because in the literature the methodology used was another one, and the crystallography.

Flavonoid 6 obtained the best probability of interaction at the active site of the 1PZ4 protein but did not obtain a probability of interaction at the active site of the 1YIY protein. All other flavonoids were likely to fit and interact at the active site of the two studied proteins. These results can be seen in Table 4.

**TABLE 4:** Summary of the results of the molecular docking of flavonoids for proteins 1PZ4 and 1YIY, probability of the RF model, and combined probability of the two virtual screening techniques.

Molecule	Prob 1PZ4	Prob 1YIY	P_RF_	Prob_Comb_ 1PZ4	Prob_Comb_ 1YIY	Multitarget
**6**	1	0	0.58	0.71	0.36	
**7**	0.80	0.93	0.54	0.69	0.74	X
**8**	0.83	1	0.53	0.67	0.73	X
**9a**	0.96	0.84	0.60	0.70	0.66	X
**9b**	0.93	0.86	0.59	0.75	0.62	X
**10**	0.97	0.99	0.55	0.65	0.75	X

Prob—Probability of interacting at the protein’s active site; PRF—Probability of the RF model; Prob_Comb_—Combined probability of the two virtual screening techniques, docking and RF model.

Calculations were also made to select multitarget molecules through the docking, combined with the virtual screening model, that is, to select molecules that are potentially active for the two enzymes, and which were also predicted to be active in the created virtual screening model. For these calculations, the following formula was used:

ProbComb= (Prob+(1+EspCross)x PRF2+EspCross , If ProbComb >0,5

Where, Prob is the potential probability, Esp_Cross_ is the value of the specificity in the cross-validation of the virtual screening model, and P_RF_ is the probability of each flavonoids being active in the RF model. And this combined probability is conditioned, that is, only molecules with values above 0.5 will be considered potentially active (Acevedo et al., 2018; Barros et al., 2019). Already eliminating also, the two molecules that had results considered not reliable by the model. The results are presented in Table 4.

Only flavonoid **6** was not multitarget since it was not likely to interact at the active site of the 1YIY protein. Also analyzing the results of the combined probability between the molecular docking and the RF model, we can see that the flavonoids that have the sulfate group in position 7 were shown to be more potentially active in both the model and in the 1PZ4 protein, a protein that is present in the larvae intestine. *Ae. aegypti* mosquito suggests that the sulfate in this position favors the fitting of the flavonoid in the active site of the protein.

While flavonoids that have the sulfate group in position 8 were more likely to have an active potential in 1YIY, a protein present in the head of the *Ae. aegypti* adult mosquito, and thus it is suggested that in this position the flavonoid presents a better fit into the active site of the protein.

#### 3.1.3 Simulations of Molecular Dynamics

**FIGURE 4:**
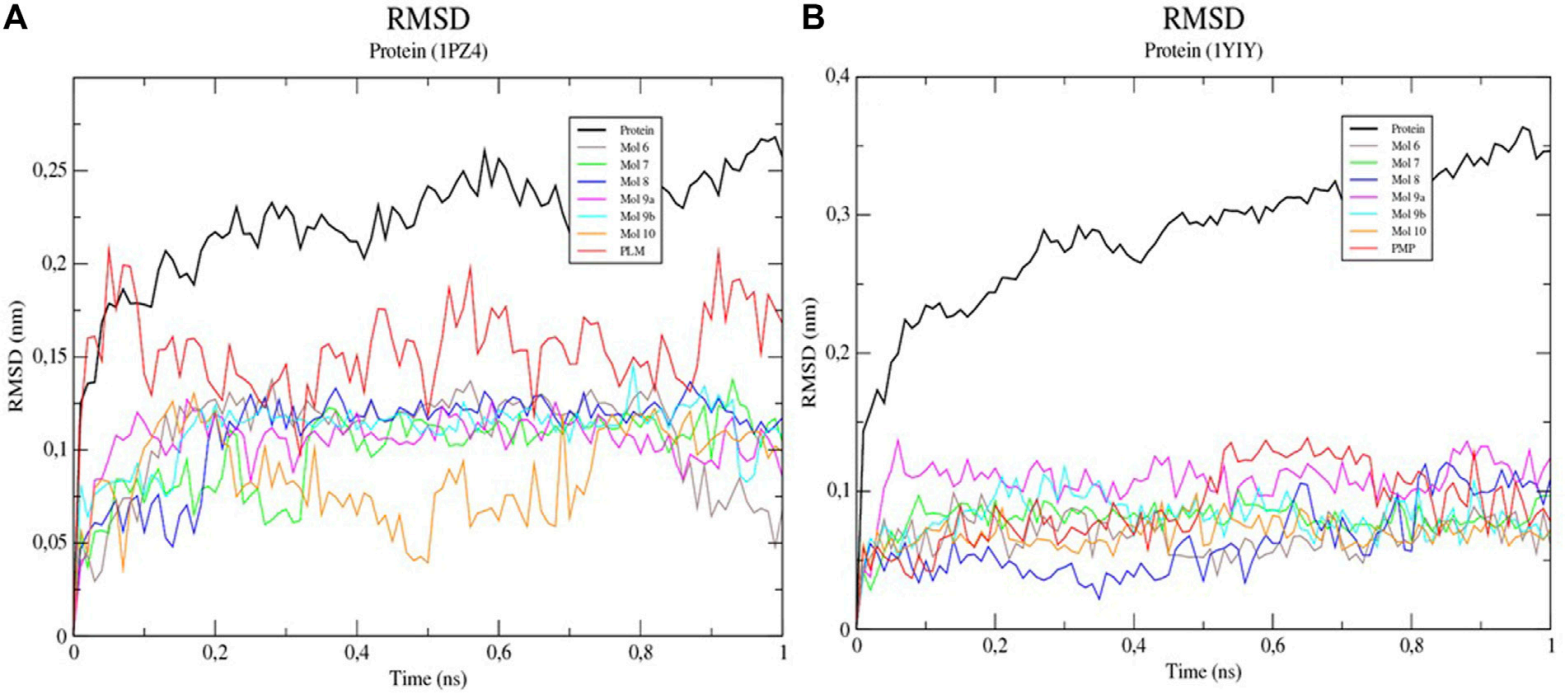
Root-mean-square fluctuation (RMSD) values for the Cα atoms of enzymes **(A)** 1PZ4 and **(B)** 1YIY, complexed with sulfated flavonoids and the PDB PLM ligand.

**FIGURE 5:**
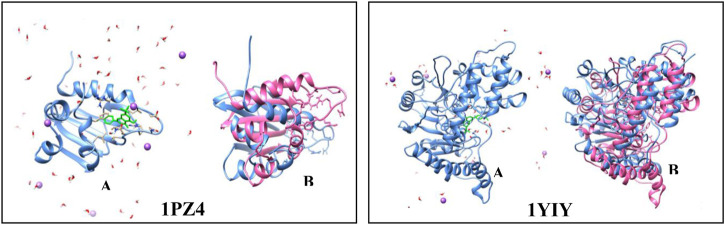
Simulation of molecular dynamics. **(A)** protein (blue) complexed to the sulfated flavonoid binder (green) in the presence of water (red and white) and ions (purple). **(B)** protein before the simulation (blue) and after the simulation in 100 ps (pink).

To characterize the interactions and to evaluate the interaction energies between the proteins and the ligands, simulations of molecular dynamics (MD) were performed. The analysis of the mean square deviation (RMSD) of the sterol protein (PDB ID 1PZ4) showed that the enzyme reached conformations ranging from 1,5 to 2,4 nm in size in 1 ns. While Kinurenine aminotransferase (PDB ID 1YIY) protein showed a RMSD with conformations ranging from 1.5 to 3.5 nm in 1 ns. The results show that the protein Kinurenine aminotransferase is more unstable, requiring greater mobility and interaction with its ligands (Figures 4, 5).

The mean square root fluctuations (RMSF) of the individual residues were also calculated. It was verified that residues 1, 23, 25, 105, 106, and 110 of the Sterol -sulfated flavonoid complexes (Mol 6, 7, 8, 9a, 9b, and 10) presented higher fluctuation values, indicating the key residues in the conformational change of the protein structure (Table 5). Only the first residue of the Sterol-PLM complex (PDB binder) showed a high fluctuation value. Kinurenin aminotransferase complexed to flavonoids also showed several residues with high RMSF values (above 0.3); corresponding to residues 12, 13, 14, 15, 16, 16, 17, 18, 22, 64, 65, 75, 159, 160, 262, 308, 357, 423, 427, 428, and 429. While complexed Kinurenine to the PDB binder (PMP) showed few high fluctuations, occurring in residues 12, 13, 14, 73, 308, and 428. This indicates that flavonoids contribute more to the flexibility of the protein, being able to interact with a greater number of amino acids.

**TABLE 5:** Coulombic and Lennard-Jones interaction energy values.

Ligand	1PZ4		1YIY	
	Coul	LJ	Coul	LJ
**6**	−387.051	−167.457	−590.73	−109.956
**7**	−109.351	−175.751	−305.6	−163.669
**8**	−232.076	−151.949	−326.509	−103.611
**9a**	−37.2133	−182.566	−174.612	−139.341
**9b**	−197.358	−174.19	−327.629	−146.516
**10**	−83.6227	−144.315	−415.19	−141.211
**PLM**	−97.4024	−148.239	—	—
**PMP**	—	—	−593.026	−54.0718

**FIGURE 6:**
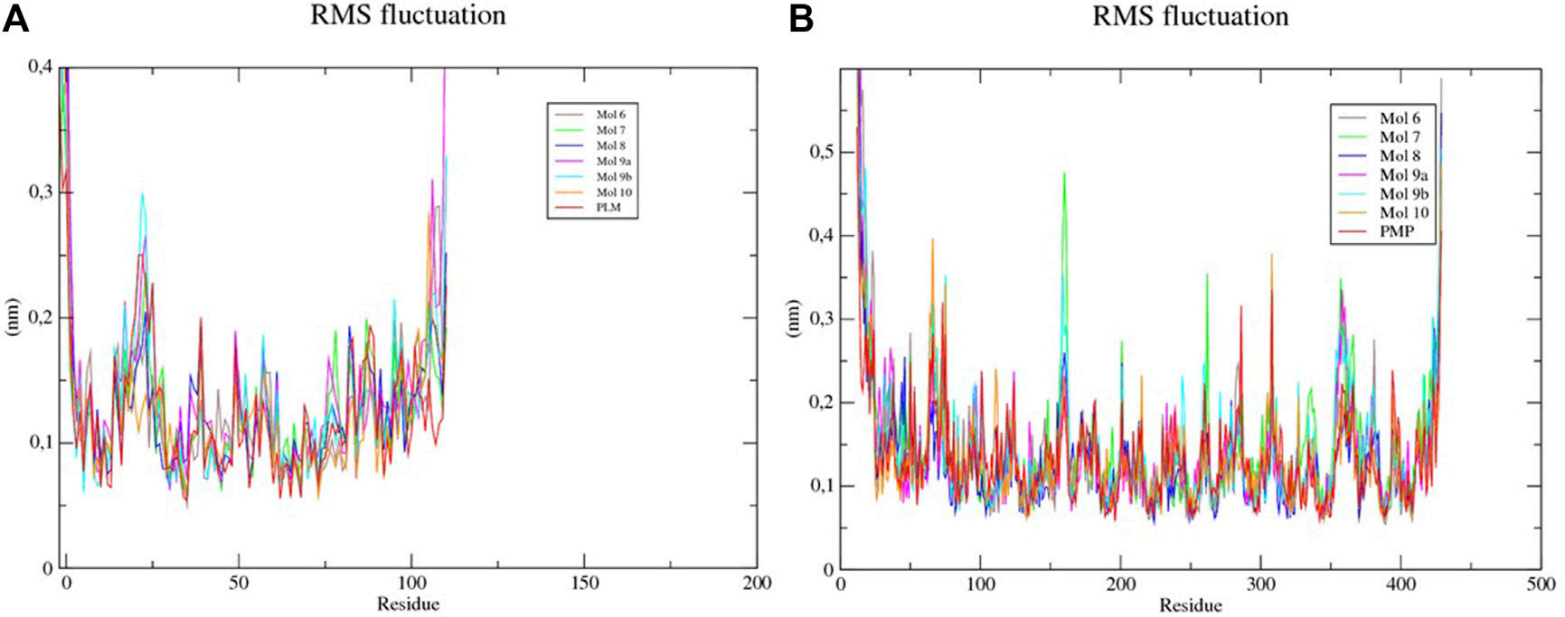
RMSF of each amino acid of the **(A)** sterol-sulfated flavonoids, **(B)** Kinurenine aminotransferase-sulfated flavonoids and -PLM complexes.

The Coulombic and Lennard-Jones interaction energies of the protein-ligand complexes were calculated and the flavonoid linkers (6, 7, 8, and 9b) were shown to have a higher interaction stability with the sterol enzyme when compared to the ligand PDB PLM (Figure 6). As for Kinurenin, all flavonoid binders showed greater interaction stability of Lennard-Jones than PDB PMP ligand (Table 5).

Although the RMSD analysis of the sterol-PLM complex is better when compared to the flavonoids, we can suggest that through RMSF and interaction energy calculations, flavonoids interact better with the two proteins because they allow greater flexibility and interaction with amino acids, and interaction stability.

## 4 Discussion

This research presented the isolation of the new sulfated flavonoid: 7-*O*-sulfate isoscutellarein (paniculatumin) (**9b**), along with eleven known compounds, including a fatty acid (**1**), a tyramine derivative (**2**), and flavonoids, contributing to the phytochemical knowledge of Malvaceae *sensu lato* family.

The production of stearic acid (**1**) by *S. paniculatum* has been mentioned recently by Fernandes et al. (2021). The fatty acids are also considered relevant chemotaxonomic markers for Malvaceae species. Some researchers have investigated the fatty acids of Malvaceae species as a useful tool to support the taxonomic study on this family (Silva et al., 2010). Based on that, fatty acids analysis has demonstrated the close taxonomic relation among the genera *Sida*, *Sidastrum,* and *Wissadula* (Fernandes et al., 2021).

In fact, species *S. paniculatum* was for a long time considered as a plant belonging to the genus *Sida,* known as *Sida paniculata* L., later reclassified by Fryxell (Cavalcante et al., 2010). Previous studies on *Sidastrum* and *Sida* species also pointed phytochemical similarities and close taxonomic relationship between these two genera (Aguilar et al., 2003; Cavalcante, et al., 2010; Gomes et al., 2011, 2015; Teles Y. C. F. et al., 2015).

Several substances identified from *S. paniculatum* have also been reported from *Sida* species, for example the identified compound N-*trans*-feruloyltyramine (**2**) has been previously isolated from the specie *Sida acuta* (Jang et al., 2003) and have shown protective effect against beta-amyloid induced neurotoxicity (Thangnipon et al., 2012).

In our study, the flavonoids were the most predominant class of identified compounds. Our findings are in accordance to the literature that reports Malvaceae family as a prolific source of phenolic and flavonoid compounds (Oliveira et al., 2012; Teles YC. et al., 2015; Vadivel et al., 2016).

We reported herein the isolation of the flavonoids acacetin (**3**), apigenin (**4**), and tiliroside (**5**). These compounds have been previously reported from Malvaceae species such as *Sidastrum micranthum* (Gomes et al., 2011), *Sida galheirensis* (Silva et al., 2006), *Sida tuberculata* (Vadivel, et al., 2016), and *Wissadula periplocifolia* (Teles et al., 2015b). Among the flavonoids identified, tiliroside (**5**) is referred to as the most prevailing flavonoid produced by Malvaceae species (Caridade et al., 2018). Several biological activities have been demonstrated to tiliroside (**5**), such as antioxidant, vasorelaxant, modulator of bacterial resistance, and anti-inflammatory and larvicidal activity against *Ae. aegypti* (Costa et al., 2007; Schinella et al., 2007; Falcão-Silva et al., 2009; Silva et al., 2013; Fernandes et al., 2019).

The phytochemical study on the polar fractions of *S. paniculatum* resulted in the isolation of the sulfated flavonoids wissadulin (**6**), 7,4′-di-*O*-metil-8-*O*-sulfate isoscutellarein (**7**), yannin (**8**), beltraonin (**9a**), the new compound paniculatumin (**9b),** and condadine (**10**).

Structures of flavonoids with *O*-sulfated substituents attached to the 2-phenyl-benzyl-γ-pyrone nucleus are considered the most uncommon flavonoids derivates found in natural products (Barron et al., 1988). The first report of the isolation of a sulfated flavonoid (isorhamnetin 3-*O*-sulfate) was published in 1937. After that, studies have pointed few species from the families Asteraceae (Compositae), Bixaceae, Malvaceae, Dilleniaceae, Umbelliferae, and Verbenaceae as producers of sulfated flavonoid (Teles et al., 2018).

Considering the family Malvaceae *sensu lato*, previous studies led to the identification of sulfated flavonoids from *Wissadula periplocifolia*, *Sidastrum micranthum,* Malva sylvestris and *Helicteres velutina* (Buchholz et al., 2007; Teles et al., 2015b; Fernandes et al., 2018; Teles et al., 2018). This is the first report of the occurrence of sulfated flavonoids from *S. paniculatum.*

The compounds wissadulin (**6**), 7,4′-di-*O*-metil-8-*O*-sulfate isoscutellarein (**7**), yannin (**8**), and beltraonin (**9a**) have been originally isolated from *Wissadula periplocifolia.* In fact, phytochemical investigations on *W. periplocifolia* suggested very similar metabolites production for *Wissadula* and *Sidastrum* species (Teles et al., 2014; Teles et al., 2015 Y. C. F.). It includes the production of steroids, hopane and 3,4-*seco* triterpenes, similar fatty acids composition, phenolic compounds, and N-*trans*-feruloyl derivatives and sulfated flavonoids (Cavalcante et al., 2010; Teles et al., 2014, Teles et al., 2015 Y. C. F., 2015b, Teles et al., 2015 YC.; Fernandes et al., 2021). Interestingly, *Wissadula* species used to be classified as *Sida,* as previously mentioned for *S. paniculatum*, indicating again the closeness among these three genera, and as indicated by Fernandes et al. (2021).

The compound **10** (condadine) have been previously reported from *H. velutina* along with other sulfated structures (Fernandes et al., 2018). *H. velutina* is a plant used traditionally by indigenous from Brazil as repellent and insecticidal. In this sense, recent studies have confirmed that *H. velutina* extract and its sulfated flavonoids would be related to the larvicidal and insecticidal activities against *Ae. aegypti,* demonstrating the relevance of the sulfate group (OSO_3_H) for the activity against *Ae. aegypti* (Fernandes et al., 2018; Fernandes et al., 2019; Fernandes et al., 2020).

In this perspective, we submitted the identified sulfated flavonoids to a ligand-based and structure-based virtual screening techniques that were combined to evaluate the sulfated flavonoids against *Ae. aegypti*. The obtained results indicated that when the O-sulfate group is bearing the position 7, as in the compounds wisssadulin, beltraonin, and paniculatumin, the structures were potentially active in both the RF model, and for the 1PZ4 protein. On the other hand, flavonoids with the O-sulfate group bearing position 8 (7,4′-di-*O*-methyl-8-*O*-sulfate flavone, yannin, and condadin) were showed to be more likely to bind to the 1YIY protein. Our findings indicated that *S. paniculatum* is a promising source of sulfated flavonoids with the potential to be used against *Ae. aegypti.*

## Data Availability

The original contributions presented in the study are included in the article/Supplementary Material, further inquiries can be directed to the corresponding author.
