# Supplementary material for: Sidastrum paniculatum (L.) Fryxell (Malvaceae): A Promising Source of Bioactive Sulfated Flavonoids Against Aedes aegypti L
Source: Front Pharmacol. 2022 Jan 7;12:760156. doi: 10.3389/fphar.2021.760156 (PMC8782119; doi:10.3389/fphar.2021.760156)
Supplement: Supplementary file 1 [file DataSheet1.docx]

***Sidastrum paniculatum* (L.) Fryxell (Malvaceae): a promising source of bioactive sulfated flavonoids against *Aedes aegypti* L.**

**Sany D. G. Marques^1,2^, Diégina A. Fernandes^1,2^, Yanna C. F. Teles^3^, Renata. P. B. Menezes^1^, Mayara S. Maia^1^, Marcus T. Scotti^1,4^, Maria F. Agra^5^, Tania M. S. Silva^6^, and Maria de Fátima Vanderlei de Souza^1,2*^**

^1^ Post graduation Program in Bioactive Natural and Synthetic Products, Federal University of Paraíba, João Pessoa, PB, Brazil

^2^Laboratory of Phytochemistry Prof. Dr. Raimundo Braz Filho, Department of Pharmaceutical Sciences, Health Sciences Center, Federal University of Paraíba, João Pessoa, Brazil

^3^Department of Chemistry and Physics, Agrarian Sciences Center, Federal University of Paraíba, Areia, Brazil

^4^Department of Chemistry, Exact and Nature Sciences Center, Federal University of Paraíba, João Pessoa, Brazil

^5^Deparment of Biotechnology, Biotechnology Center, Federal University of Paraíba, João Pessoa, Brazil

^6^Department of Molecular Sciences, Rural Federal University of Pernambuco, Recife, Brazil

Supplementary Material


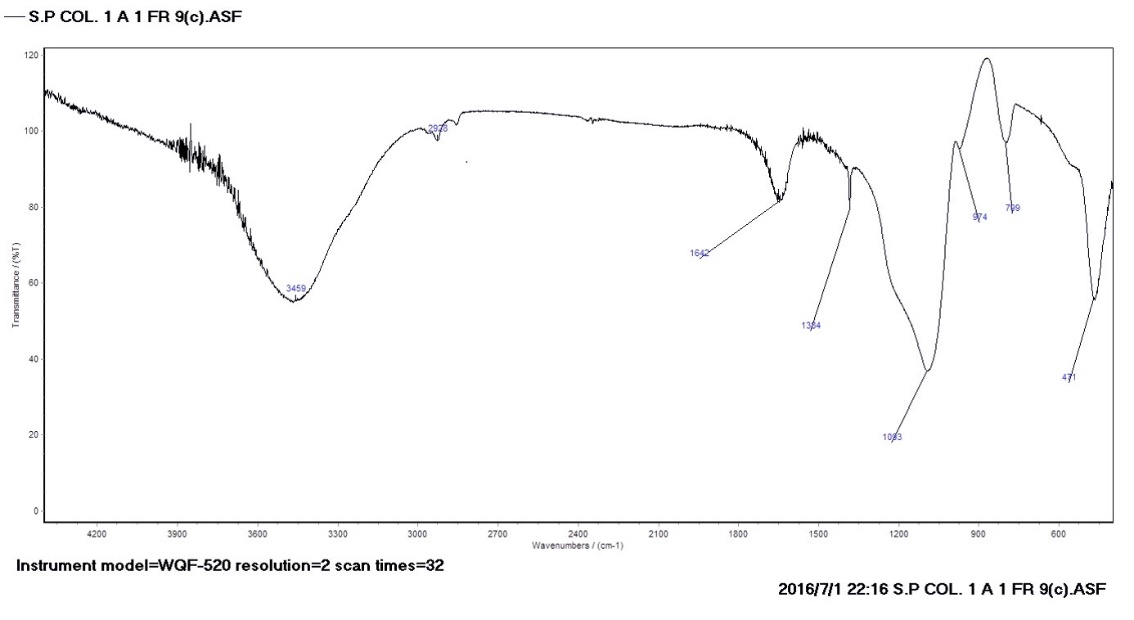


**Figure S1**. FTIR (KBr) spectrum of Compound **1**.


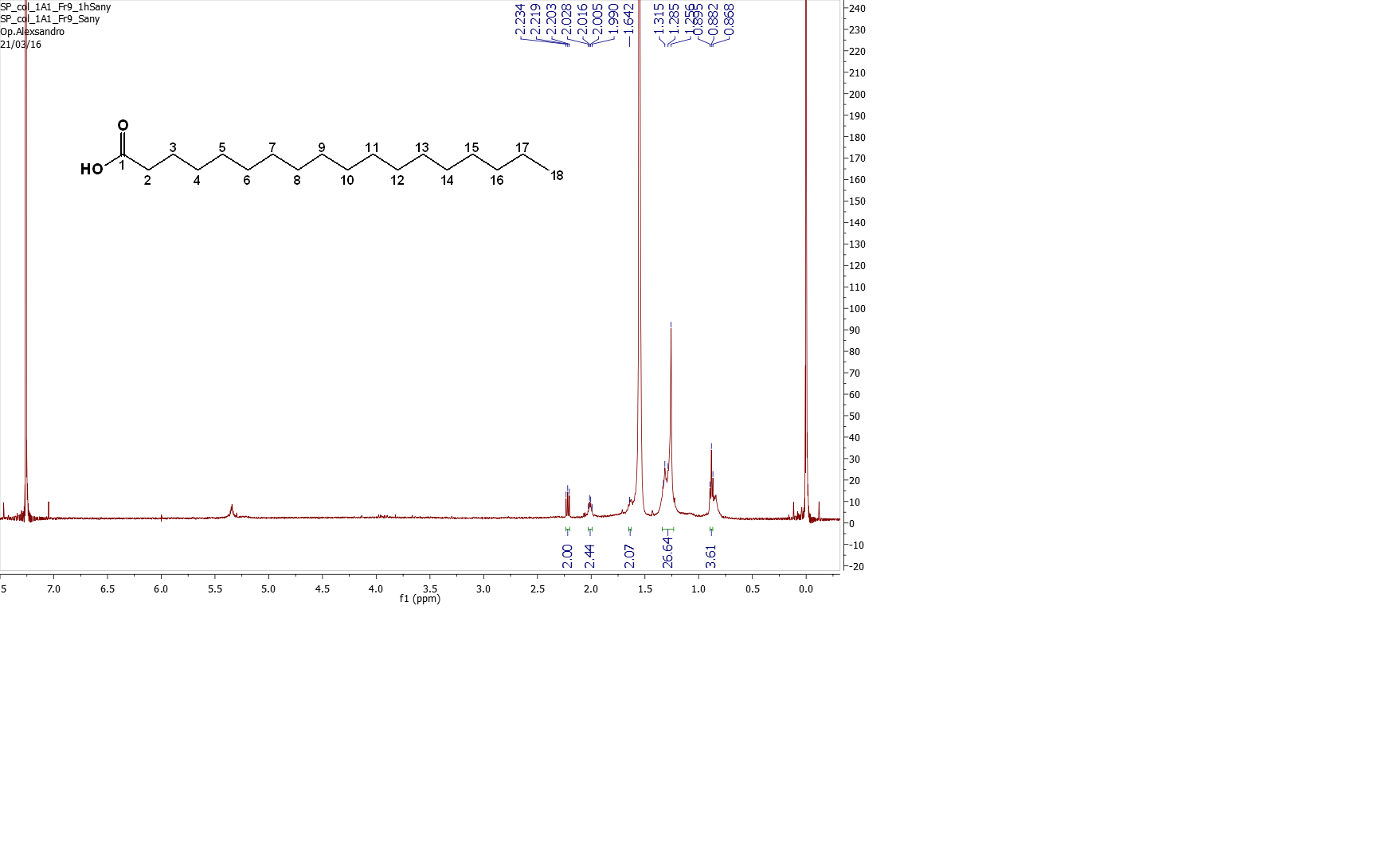


**Figure S2**. ^1^H NMR spectrum (500 MHz, CDCl_3_) of Compound **1.**


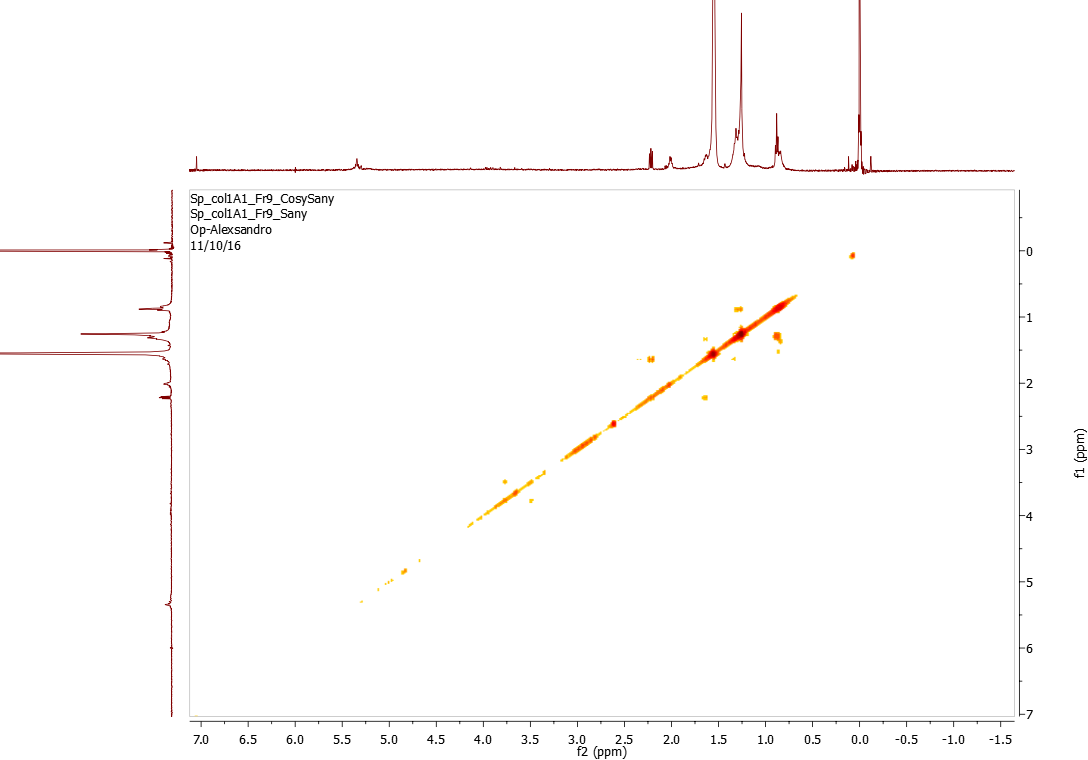


**Figure S3**. COSY spectrum (^1^H NMR: 500 MHz, CDCl_3_) of Compound **1**.


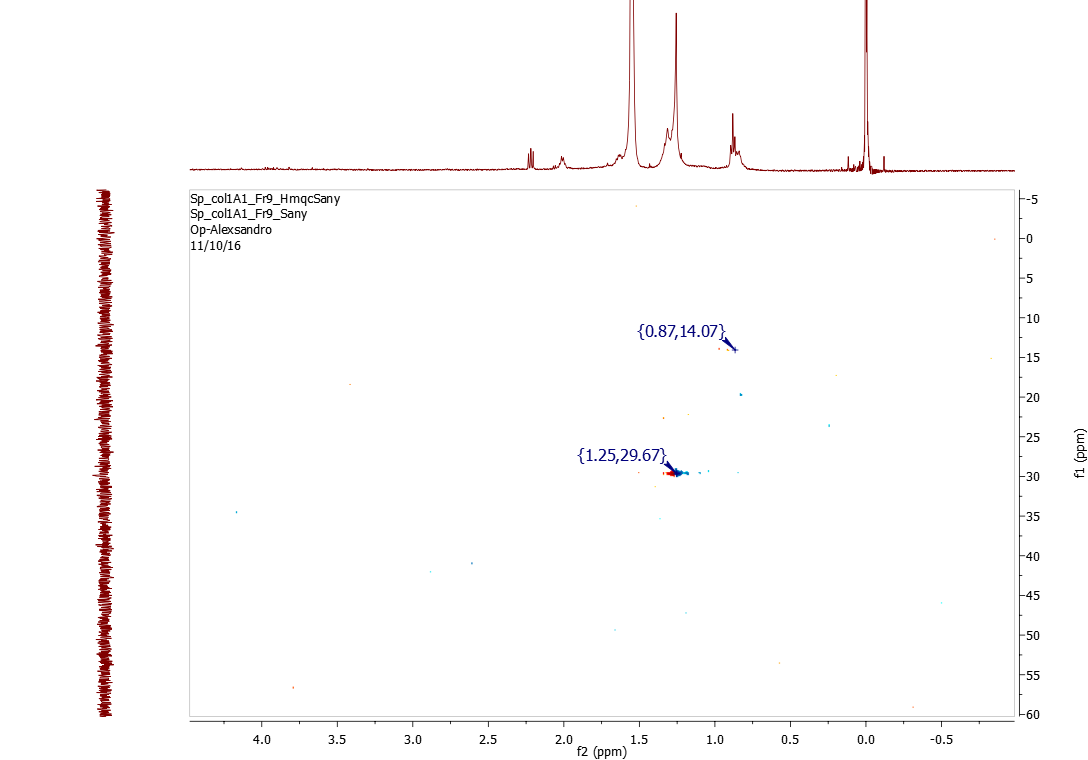


**Figure S4**. HMQC spectrum (^1^H-NMR: 500 MHz, ^13^C-NMR: 125 MHz, CDCl_3_) of Compound **1**.


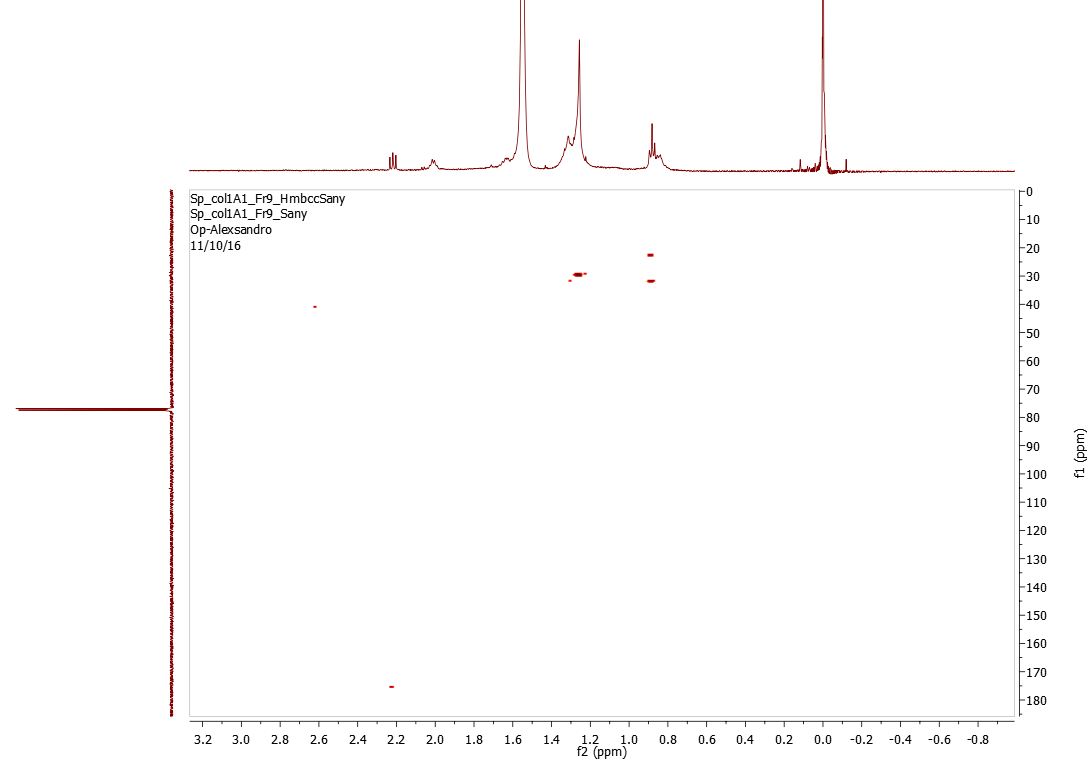


**Figure S5**. HMBC spectrum (^1^H-NMR: 500 MHz, ^13^C-NMR: 125 MHz, CDCl_3_) of Compound **1**.


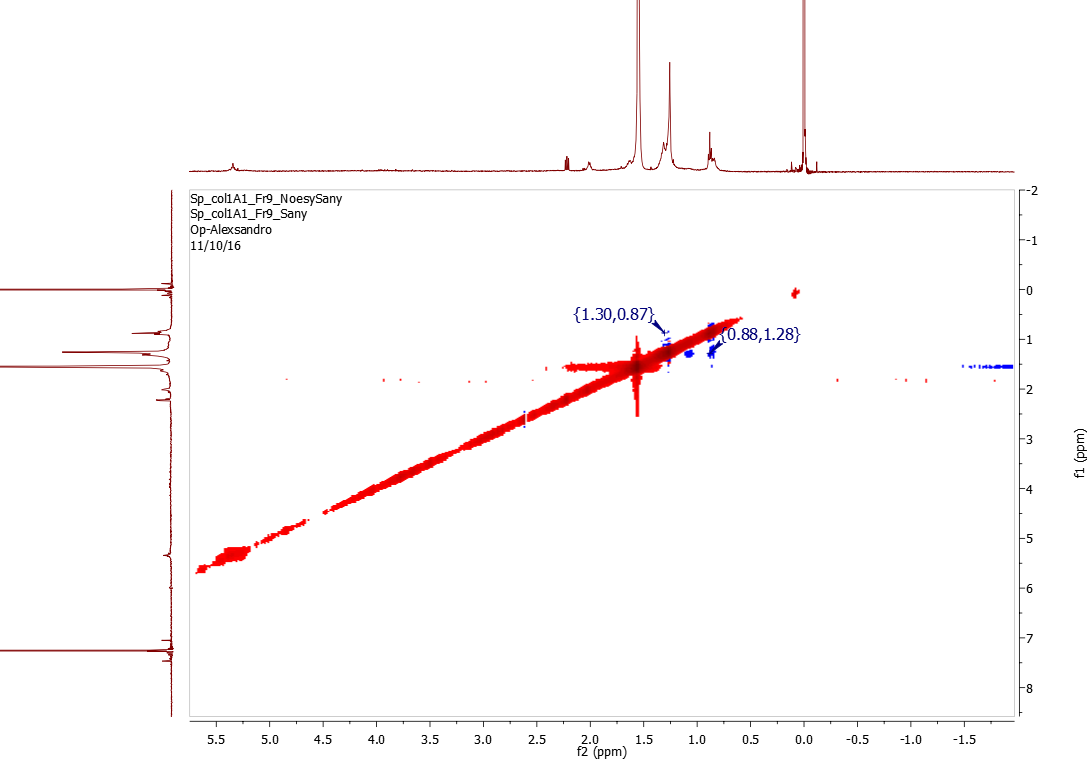


**Figure S6**. NOESY spectrum (^1^H-NMR: 500 MHz, CDCl_3_) of Compound **1**.


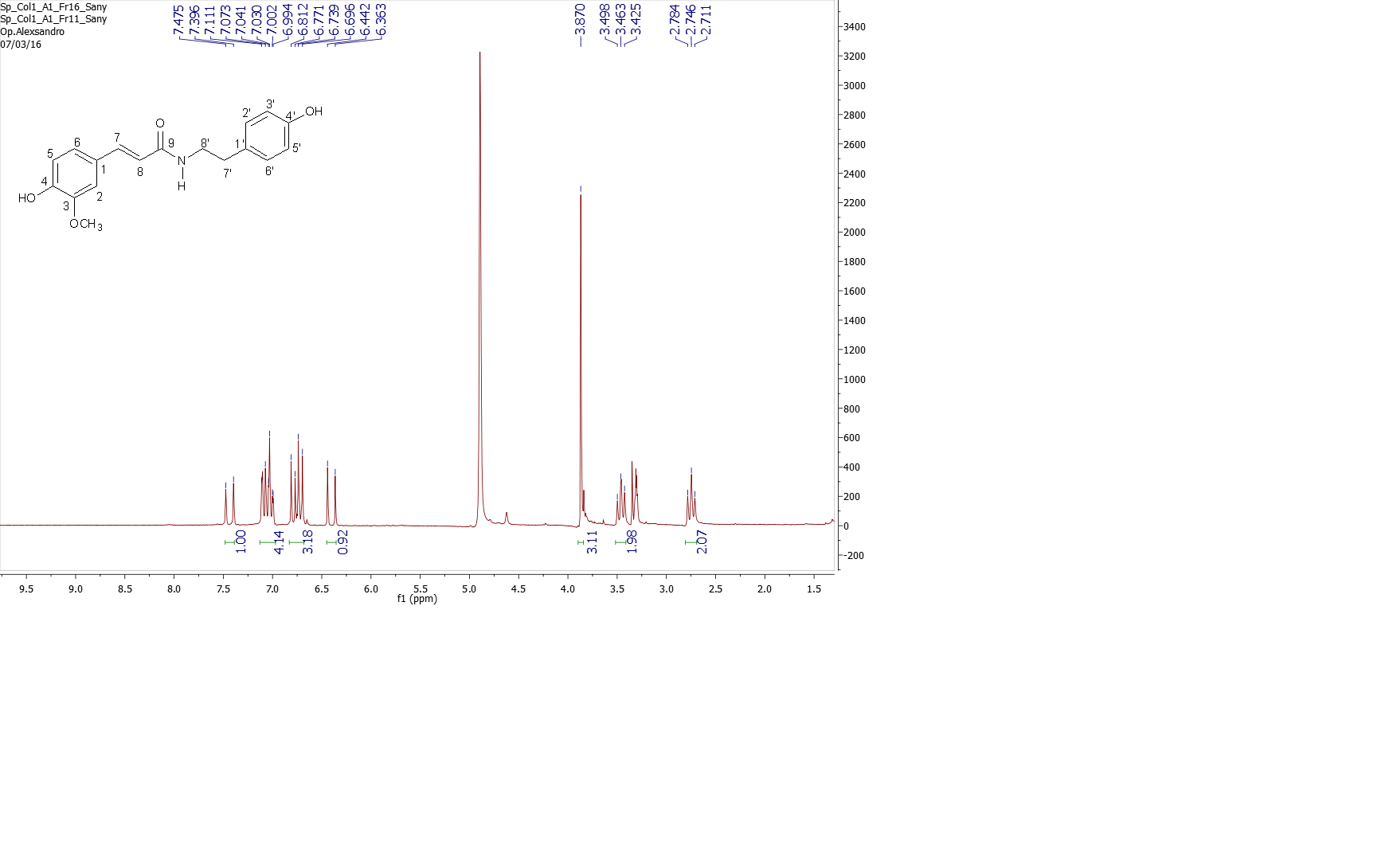


**Figure S7**. ^1^H NMR spectrum (200 MHz, CD_3_OD) of Compound **2**.


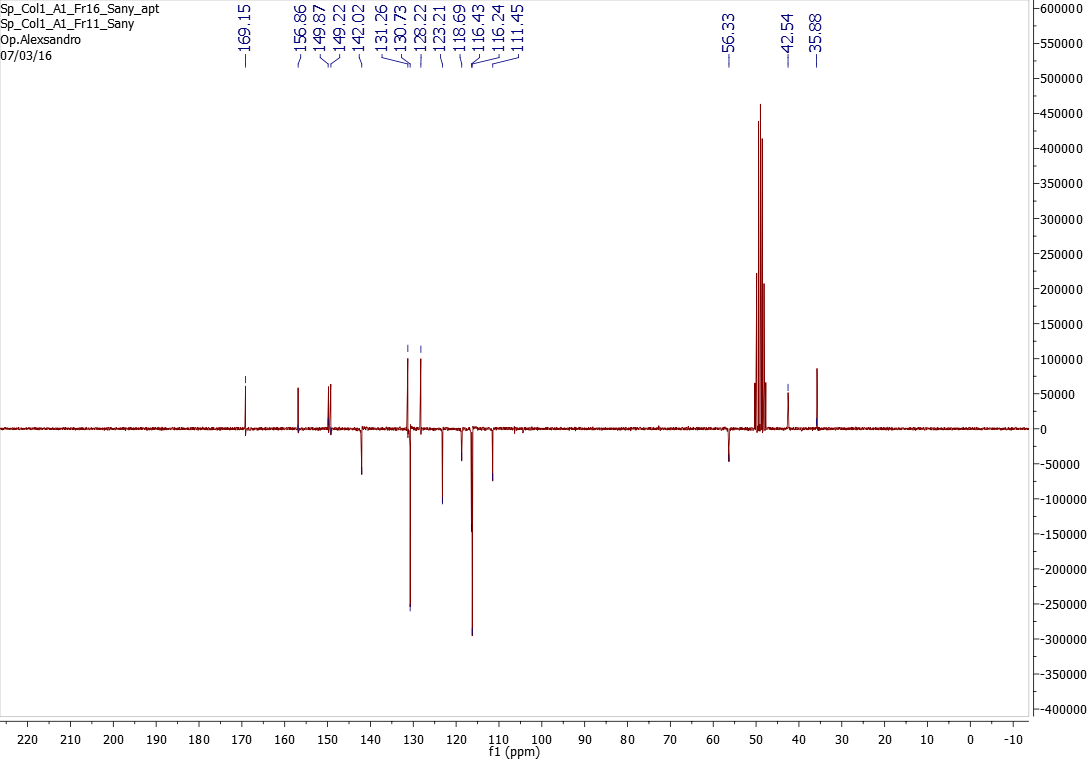


**Figure S8**. ^1^C NMR spectrum (50 MHz, CD_3_OD) of Compound **2**.


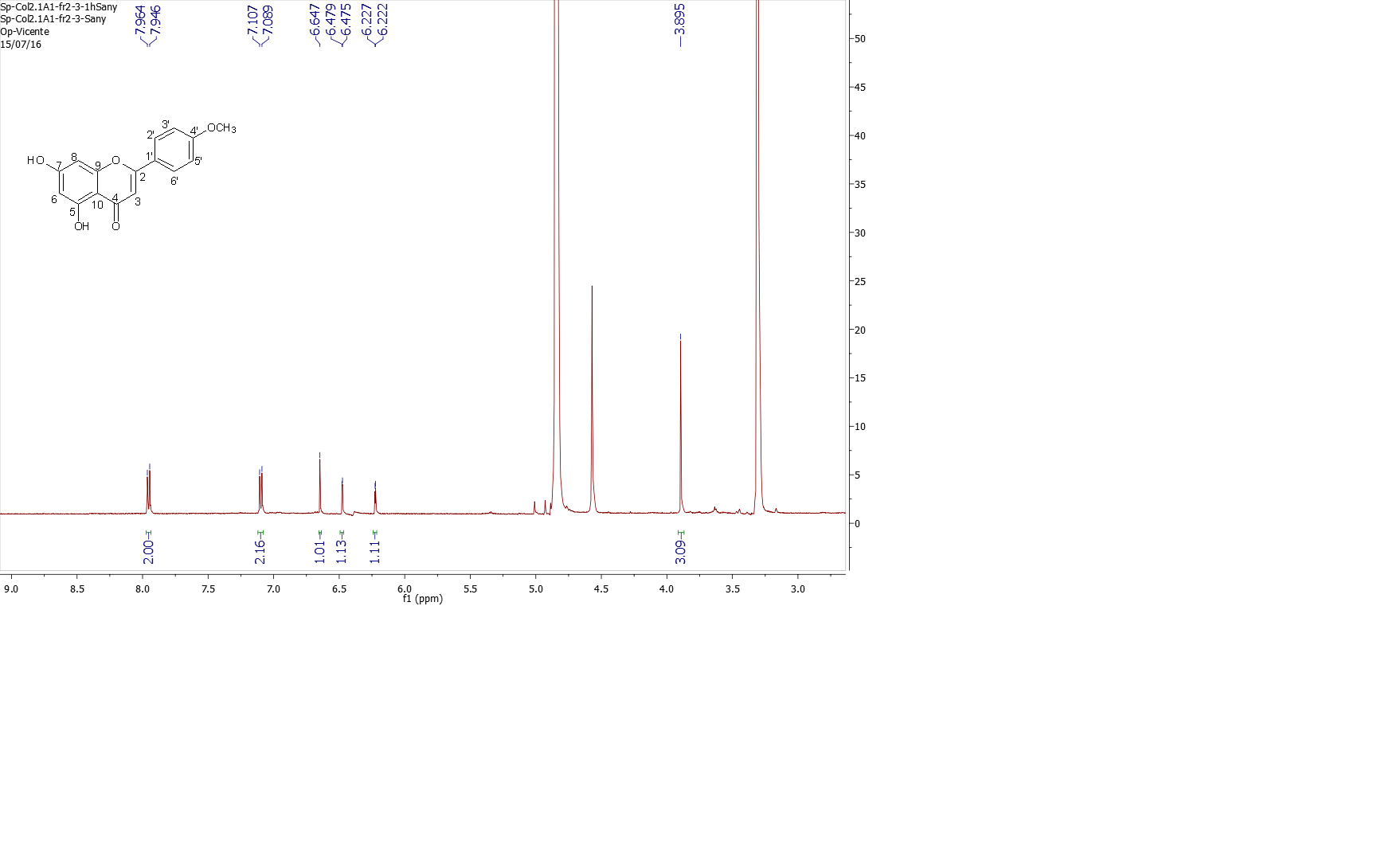


**Figure S9**. ^1^H NMR spectrum (500 MHz, CD_3_OD) of Compound **3**.


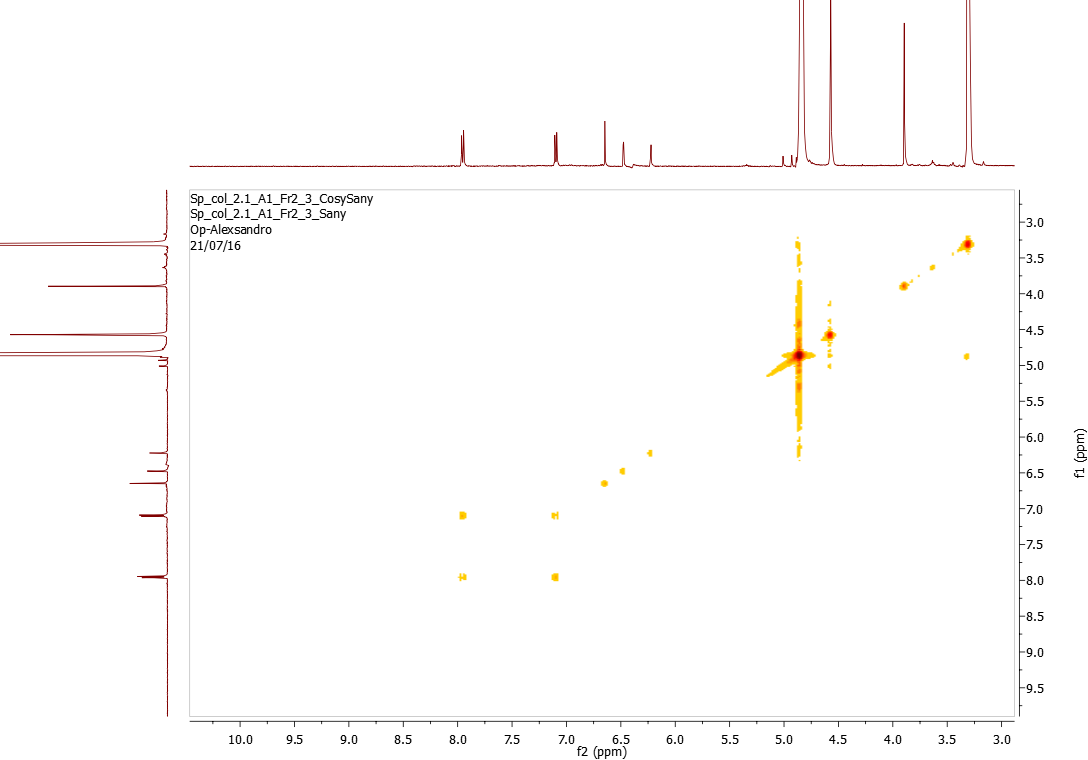


**Figure S10**. COSY spectrum (^1^H NMR: 500 MHz, CD_3_OD) of Compound **3**.


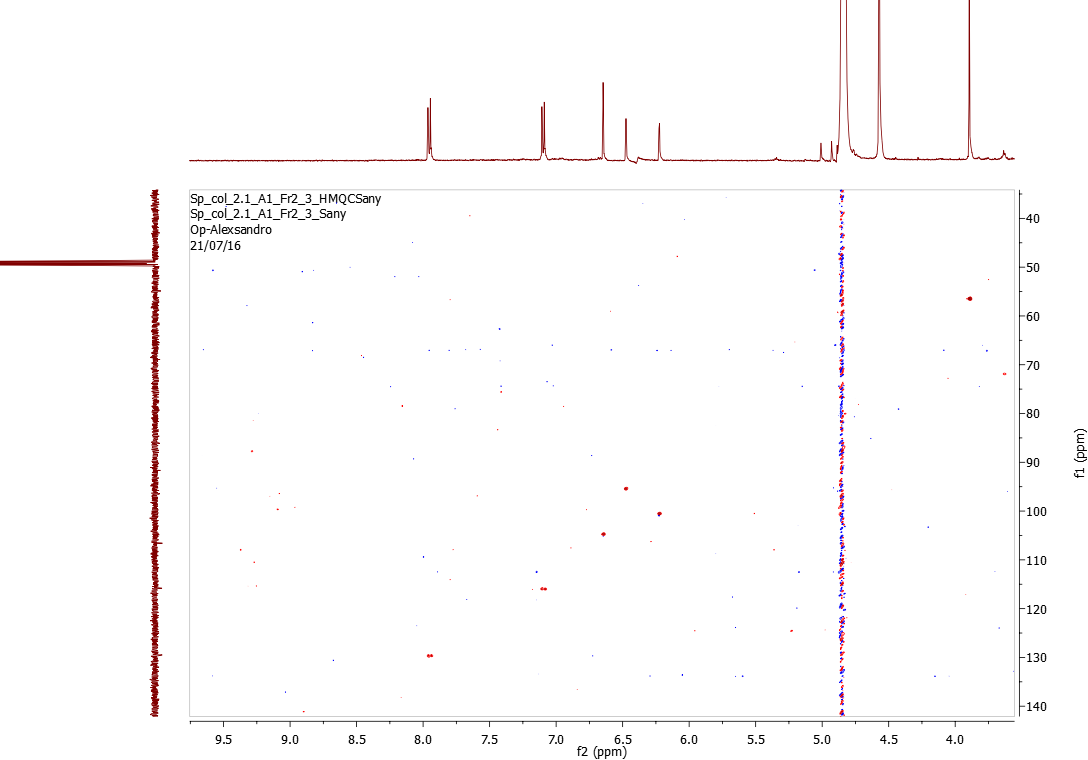


**Figure S11**. HMQC spectrum (^1^H-NMR: 500 MHz, ^13^C-NMR: 125 MHz, CD_3_OD) of Compound **3**.


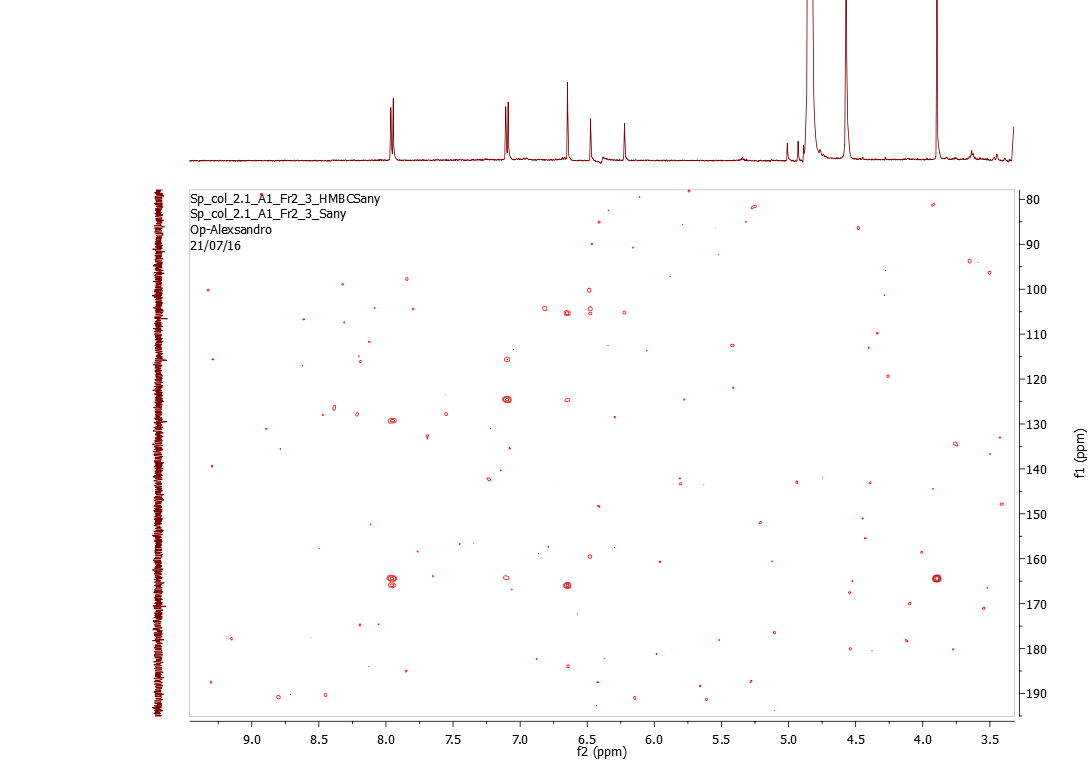


**Figure S12**. HMBC spectrum (^1^H-NMR: 500 MHz, ^13^C-NMR: 125 MHz, CD_3_OD) of Compound **3**.


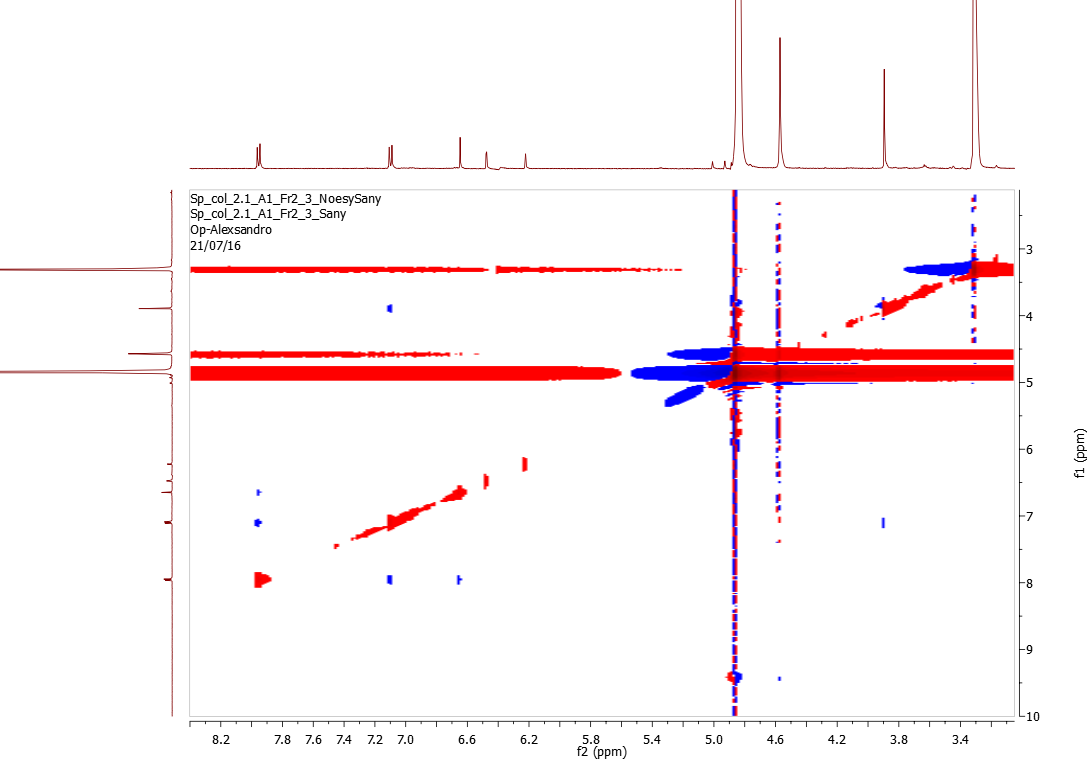


**Figure S13**. NOESY spectrum (^1^H-NMR: 500 MHz, CD_3_OD) of Compound **3**.


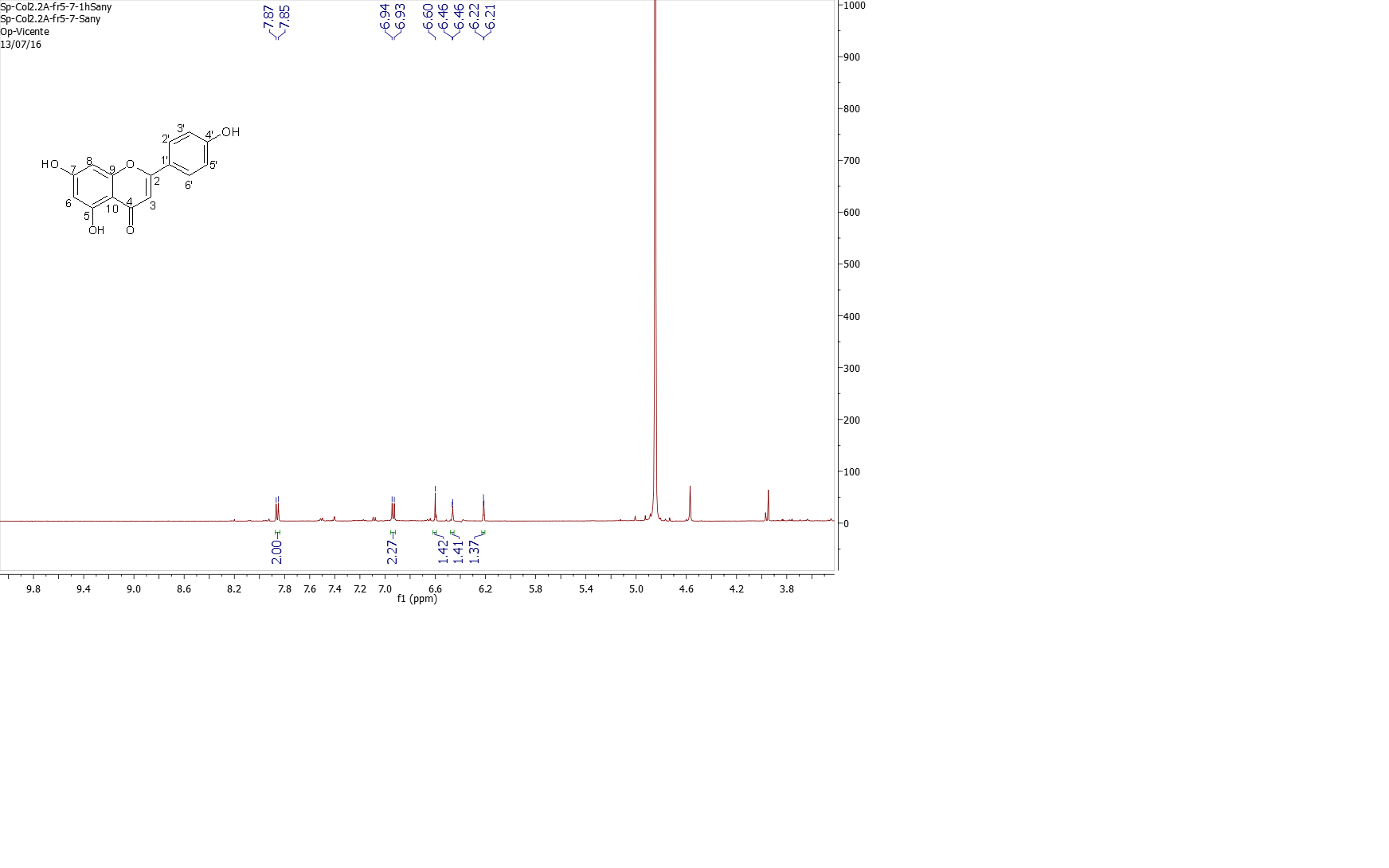


**Figure S14**. ^1^H NMR spectrum (500 MHz, CD_3_OD) of Compound **4**.


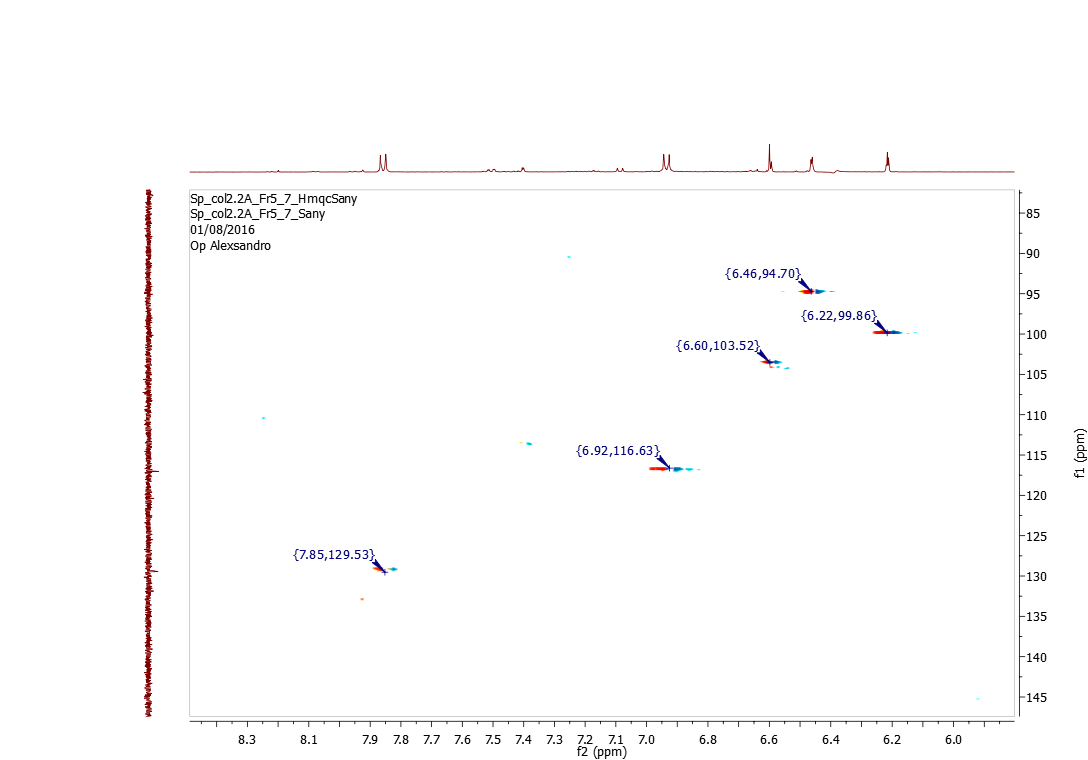


**Figure S15**. HMQC spectrum (^1^H-NMR: 500 MHz, ^13^C-NMR: 125 MHz, CD_3_OD) of Compound **4**.


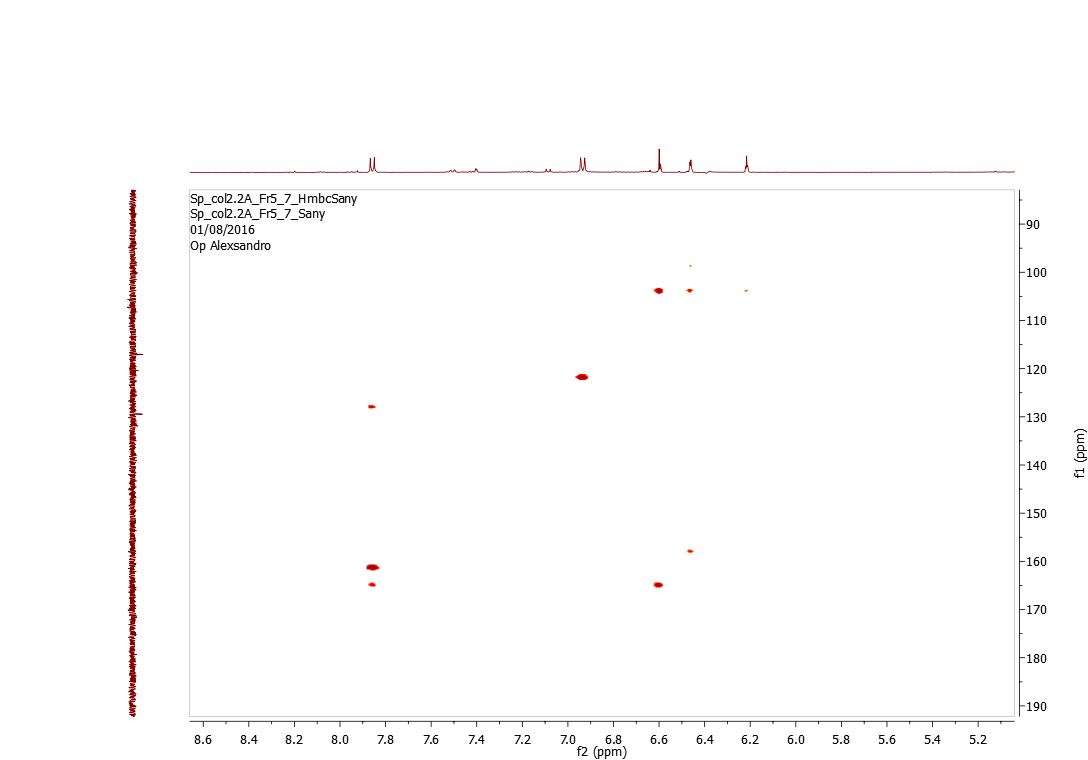


**Figure S16**. HMBC spectrum (^1^H-NMR: 500 MHz, ^13^C-NMR: 125 MHz, CD_3_OD) of Compound **4**.


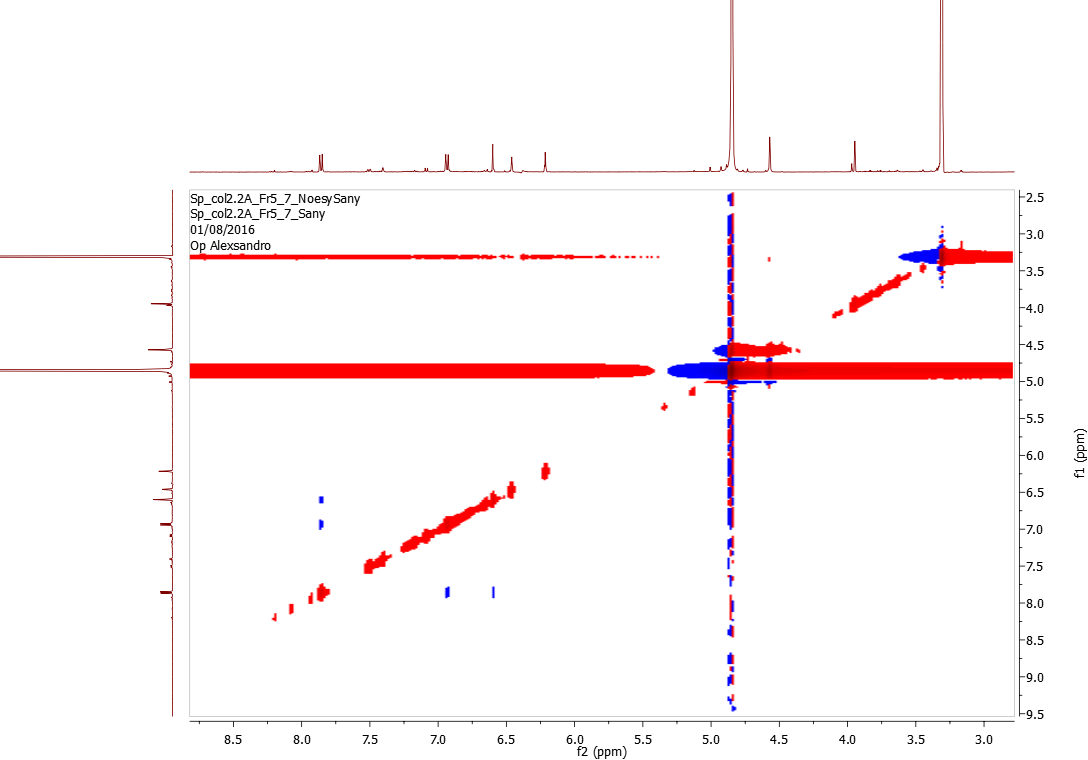


**Figure S17**. NOESY spectrum (^1^H-NMR: 500 MHz, CD_3_OD) of Compound **4**.


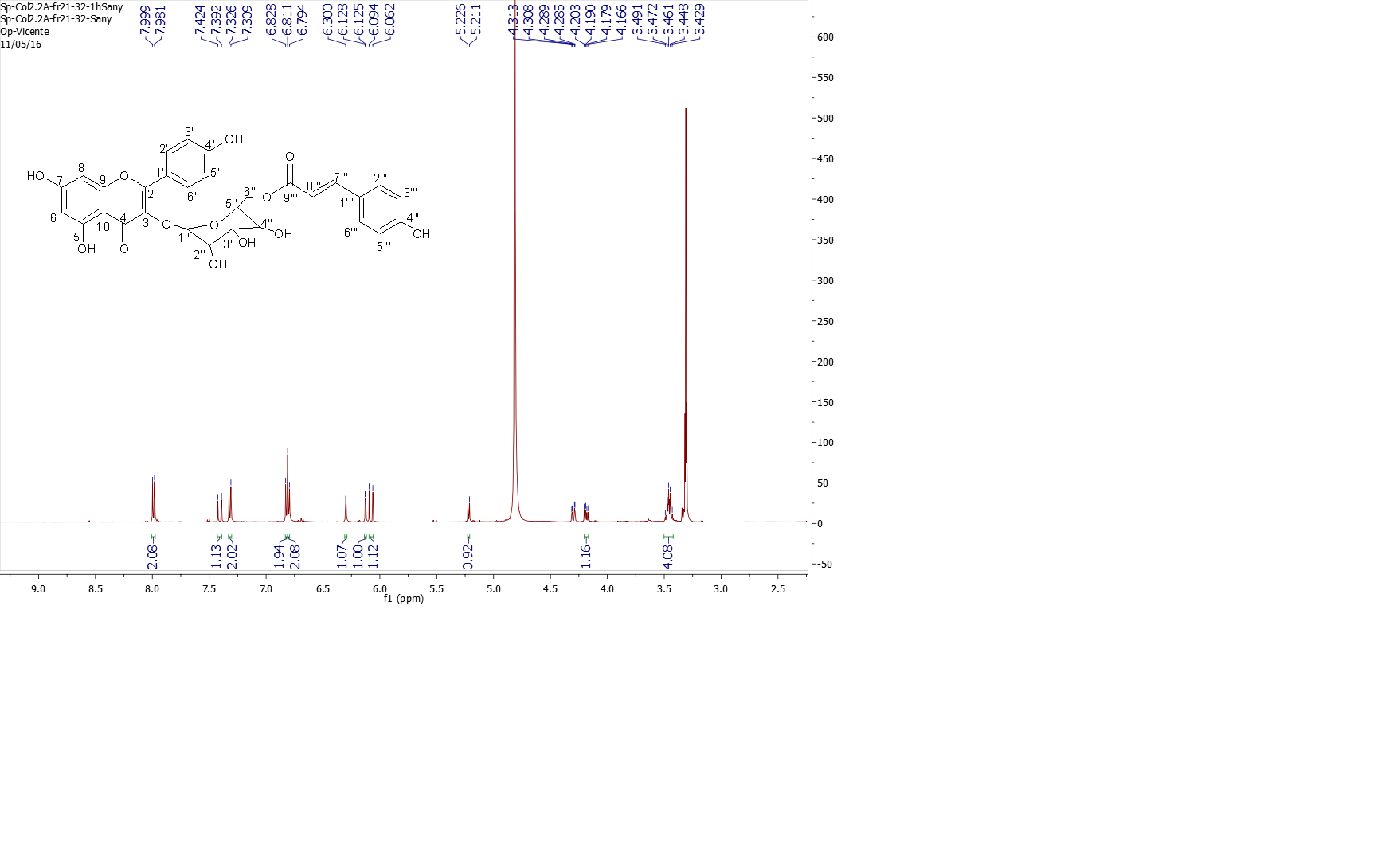


**Figure S18**. ^1^H NMR spectrum (500 MHz, CD_3_OD) of Compound **5**.


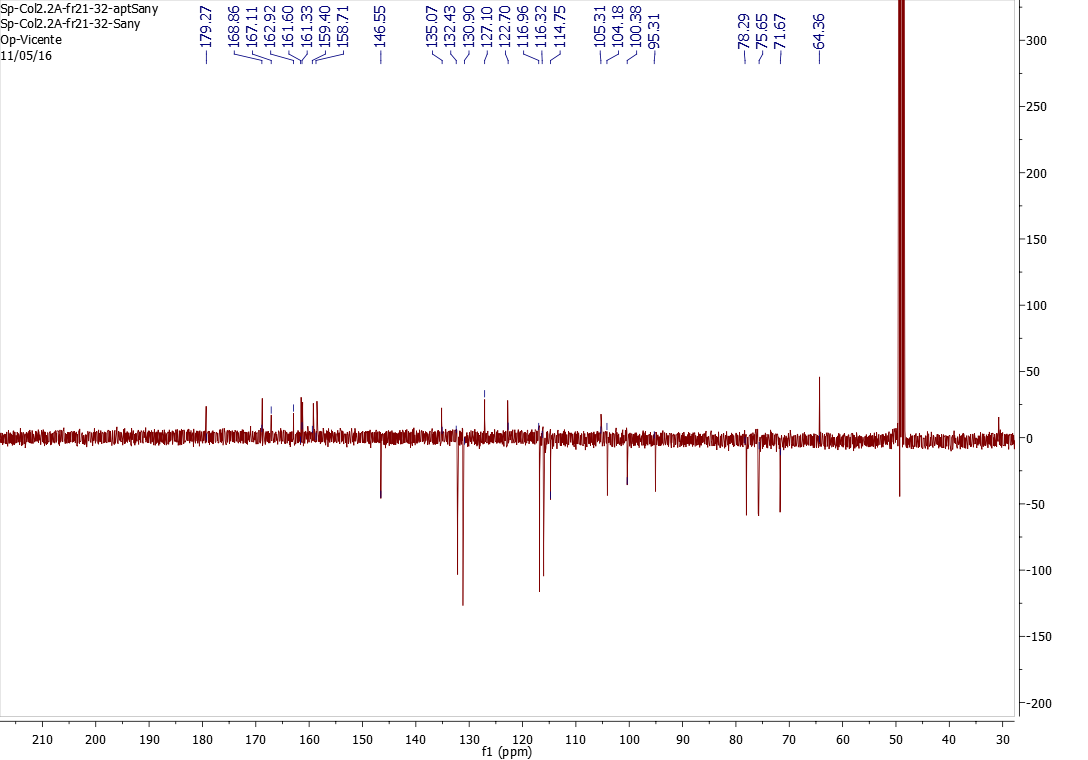


**Figure S19**. ^13^C NMR spectrum (125 MHz, CD_3_OD) of Compound **5**.


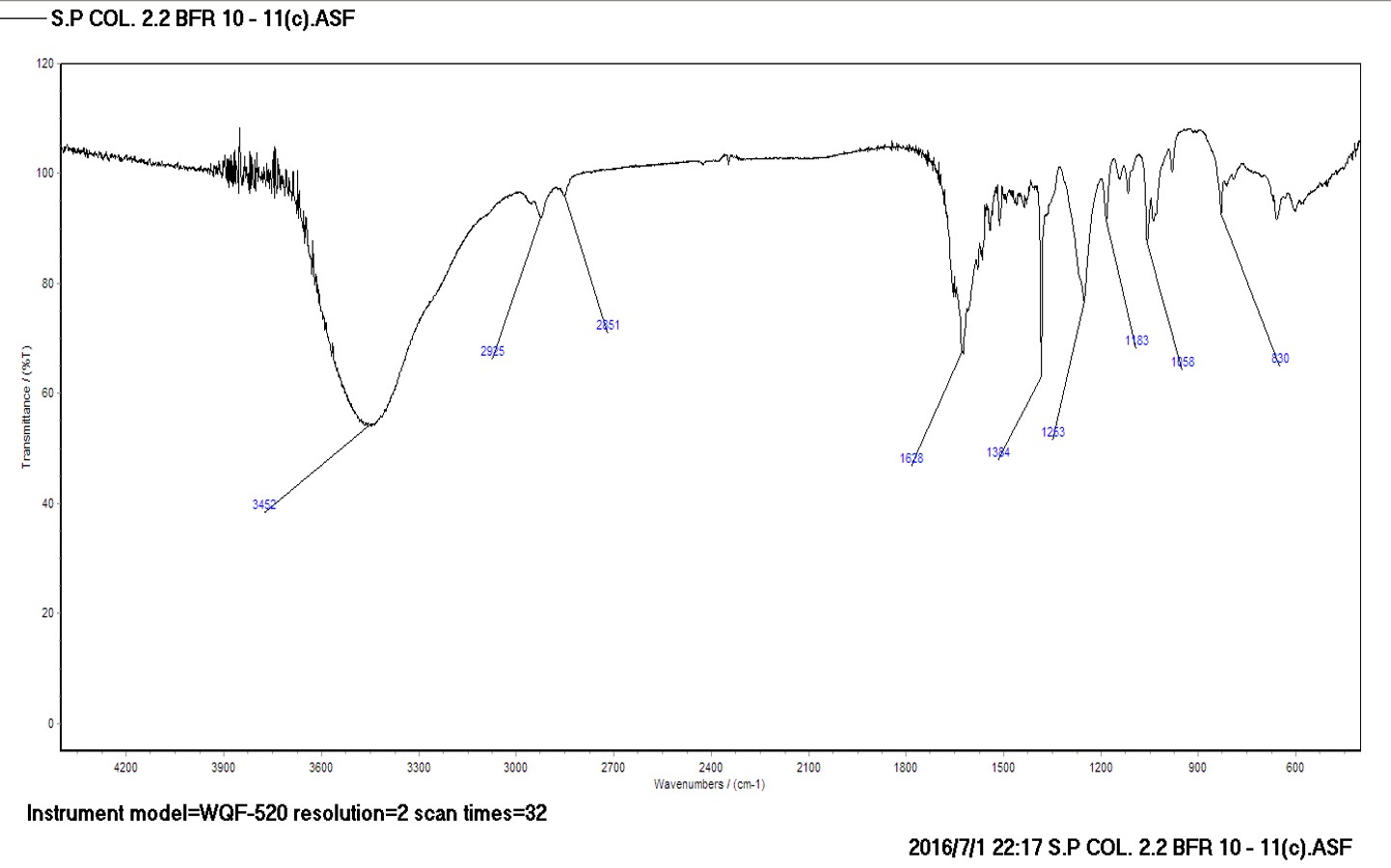


**Figure S20**. FTIR (KBr) spectrum of Compound **6**.


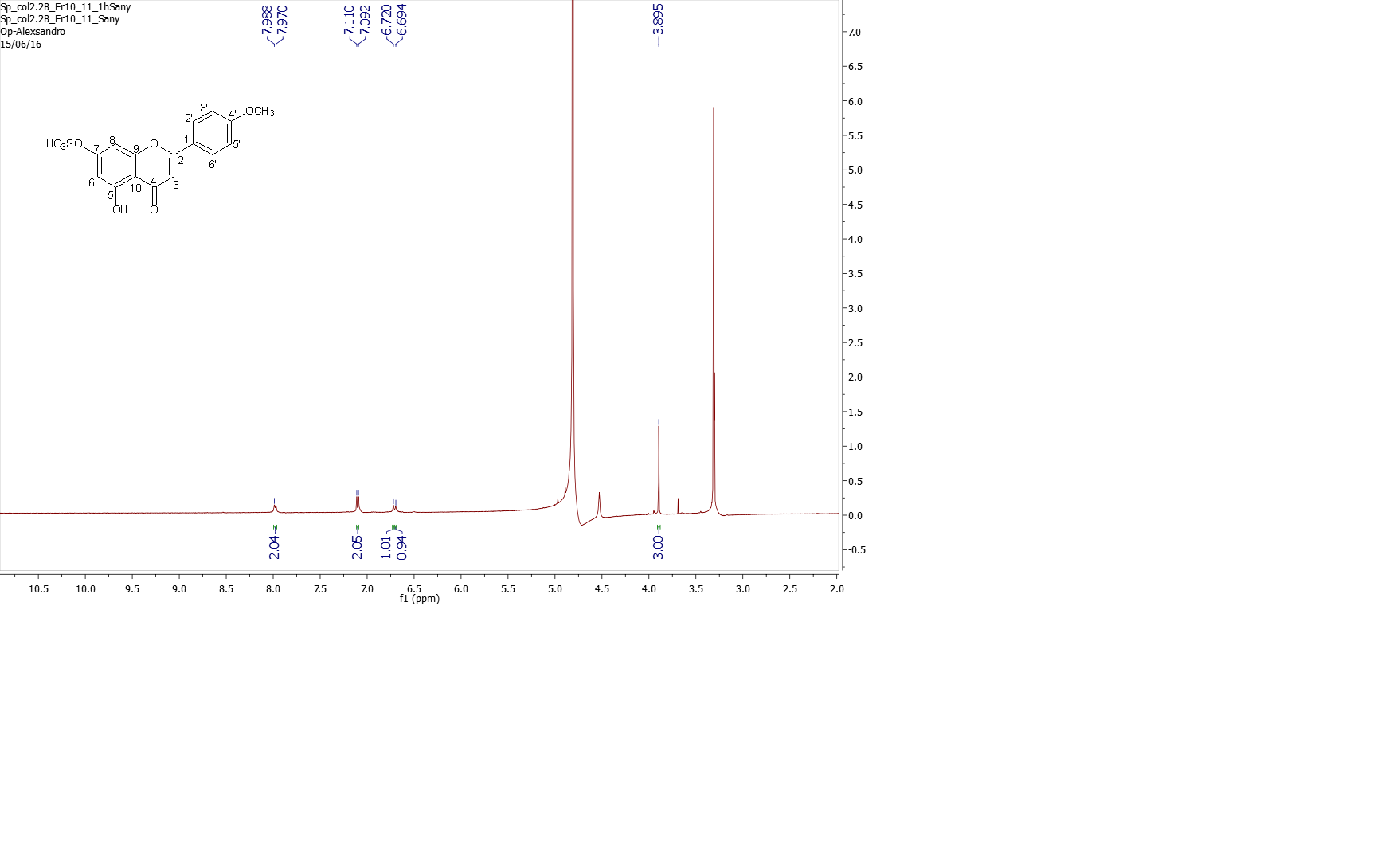


**Figure S21**. ^1^H NMR spectrum (500 MHz, CD_3_OD) of Compound **6**.


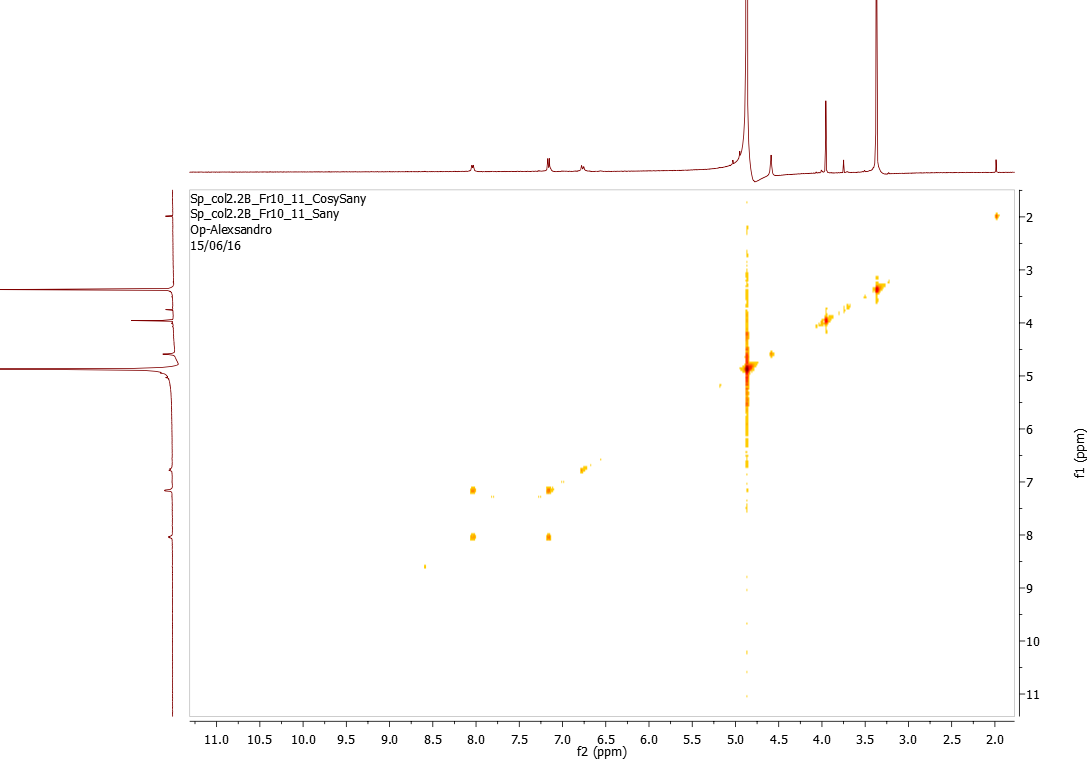


**Figure S22**. COSY spectrum (^1^H NMR: 500 MHz, CD_3_OD) of Compound **6**.


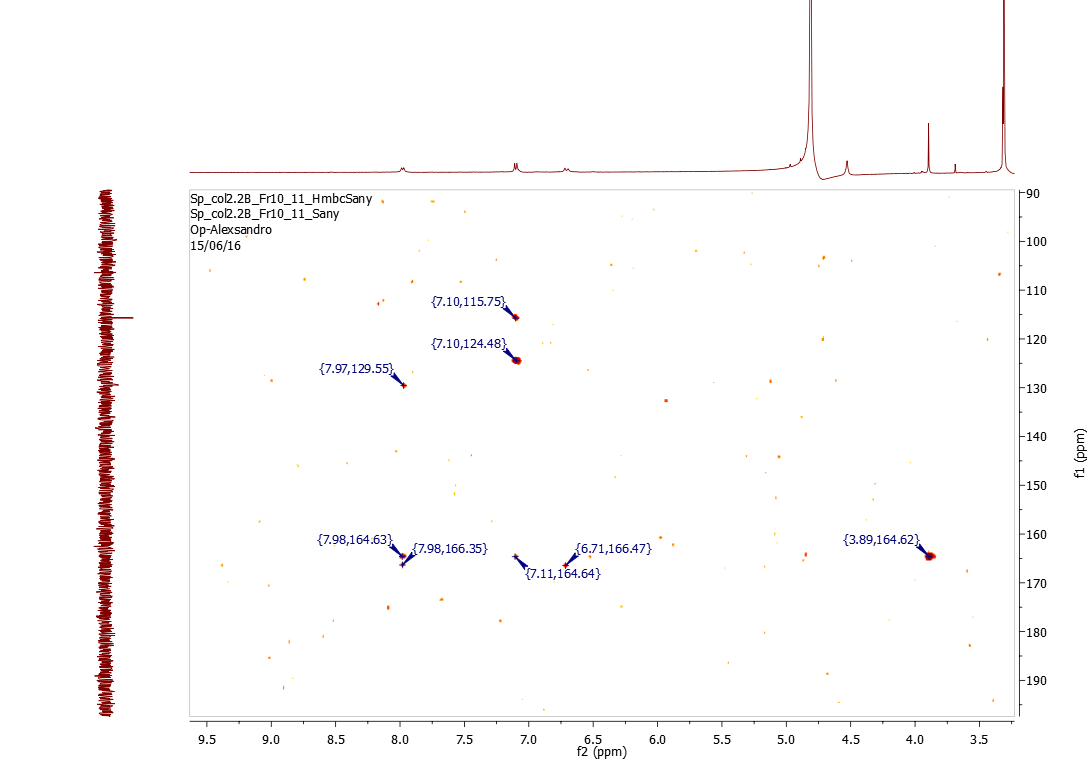


**Figure S23**. HMBC spectrum (^1^H-NMR: 500 MHz, ^13^C-NMR: 125 MHz, CD_3_OD) of Compound **6**.


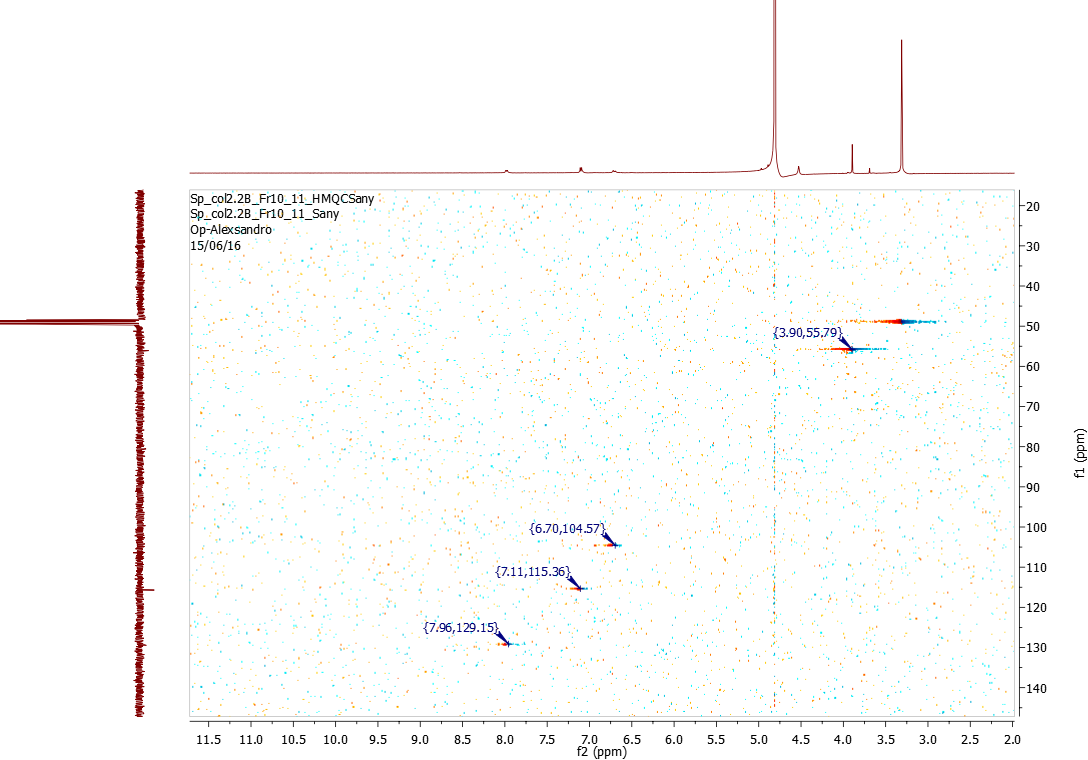


**Figure S24**. HMQC spectrum (^1^H-NMR: 500 MHz, ^13^C-NMR: 125 MHz, CD_3_OD) of Compound **6**.


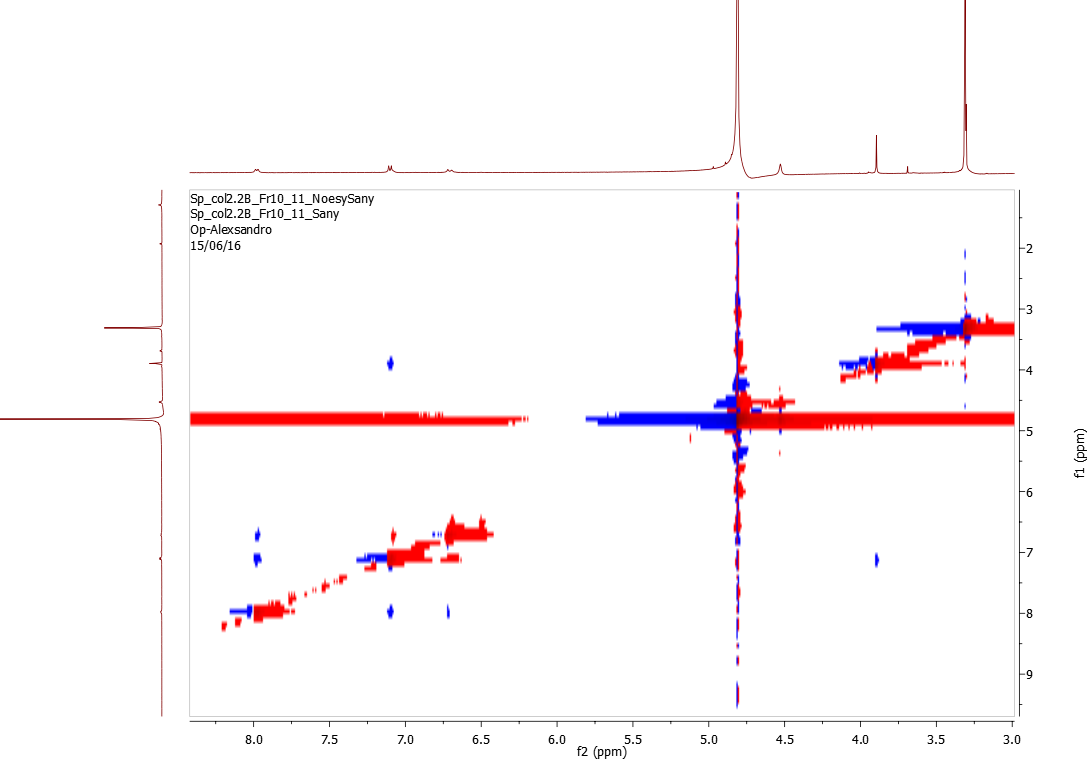


**Figure S25**. NOESY spectrum (^1^H-NMR: 500 MHz, CD_3_OD) of Compound **6**.


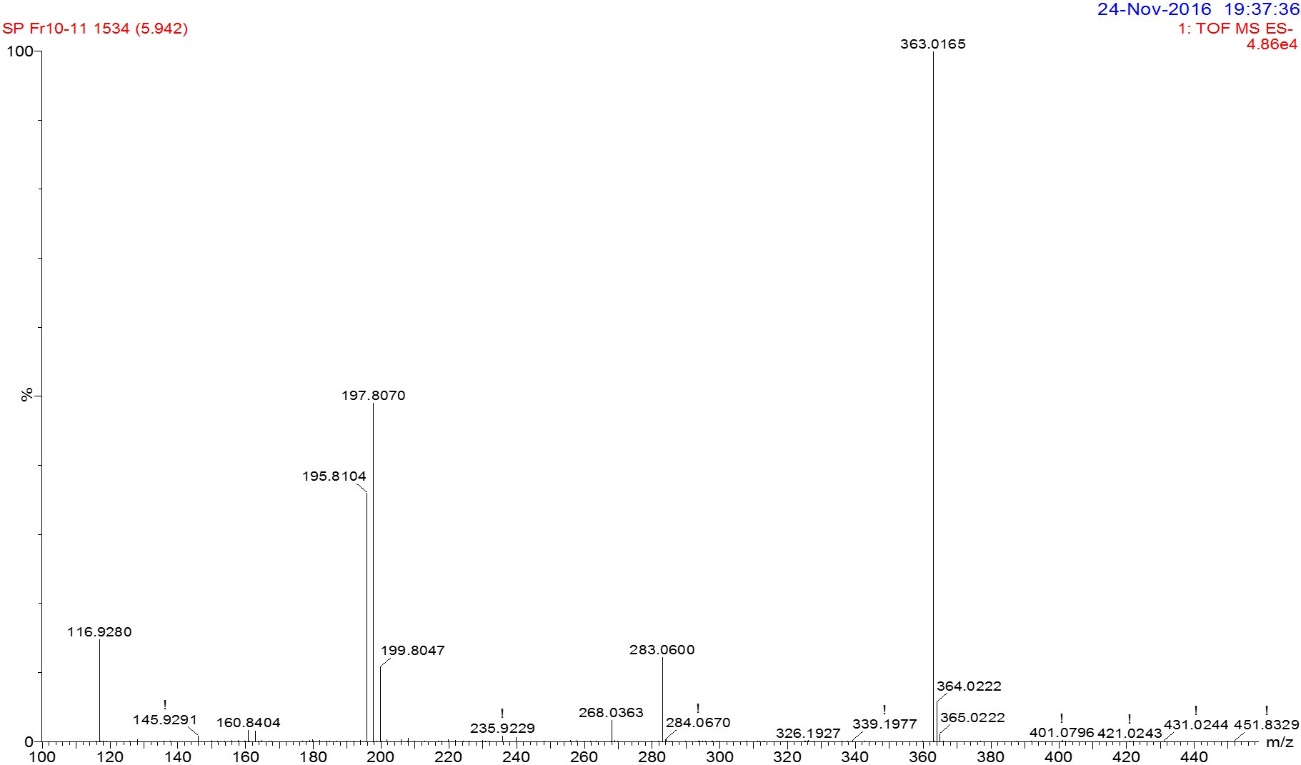


**Figure S26**. Mass spectrum of Compound **6**.


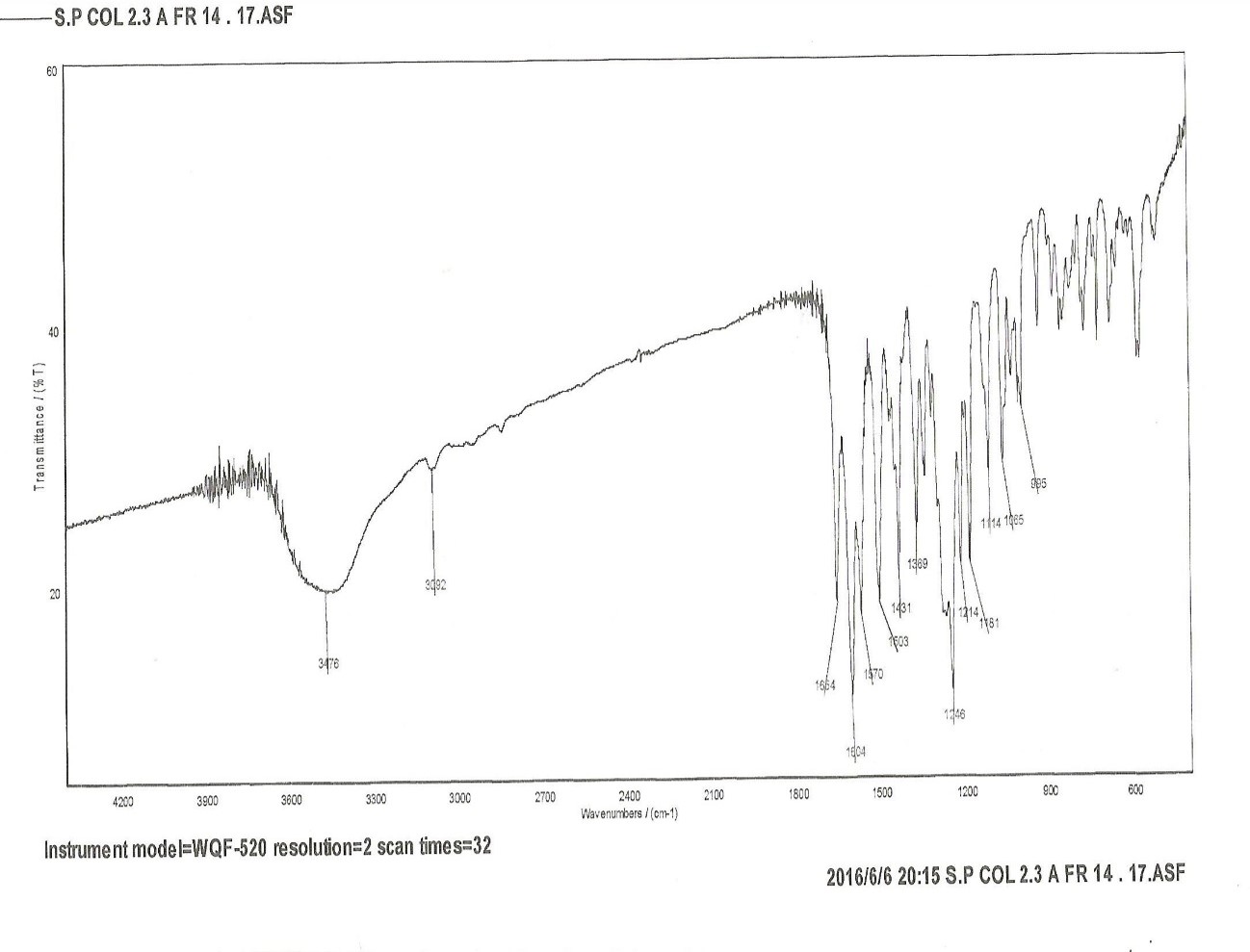


**Figure S27**. FTIR (KBr) spectrum of Compound **7**.


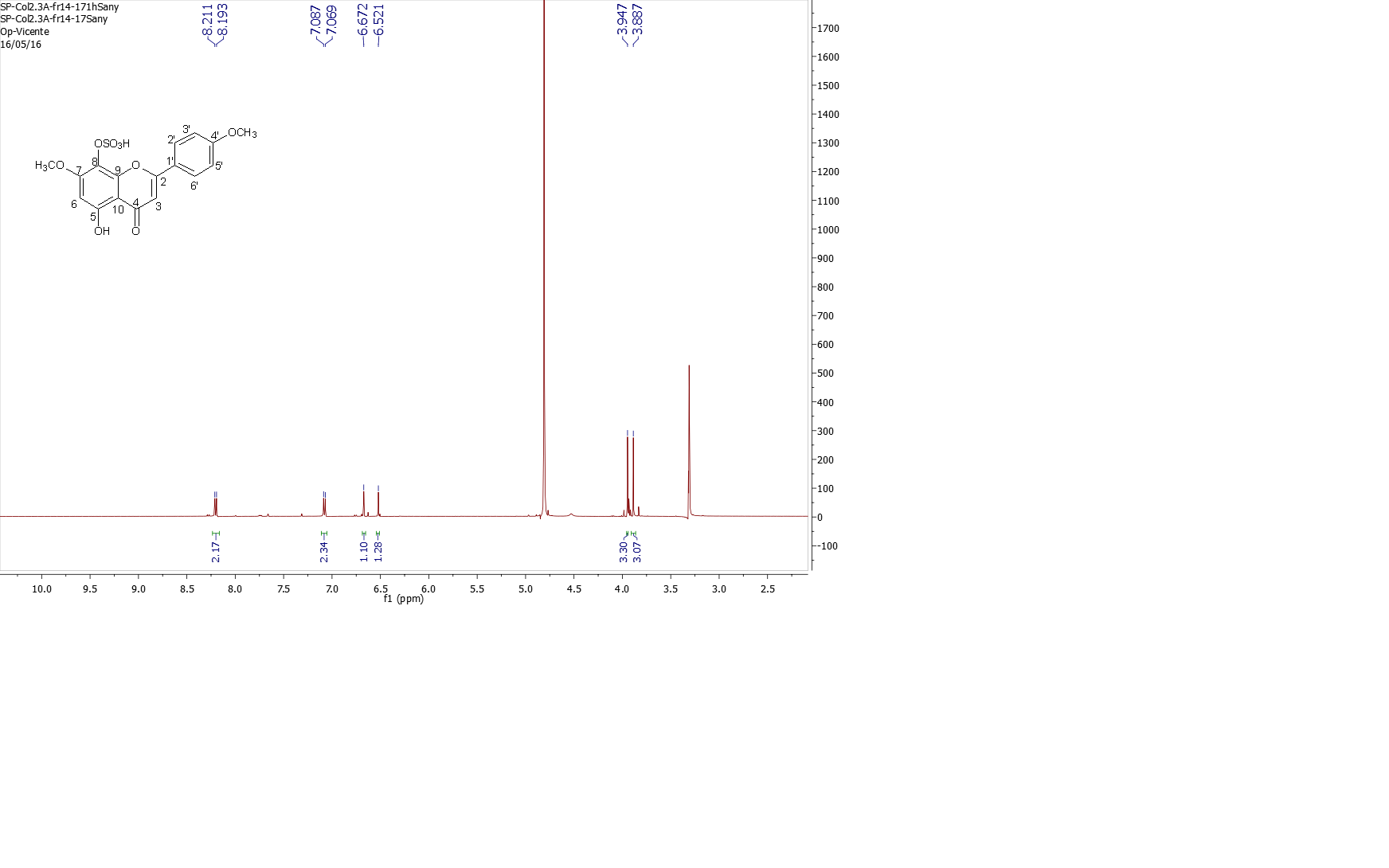


**Figure S28**. ^1^H NMR spectrum (500 MHz, CD_3_OD) of Compound **7**.


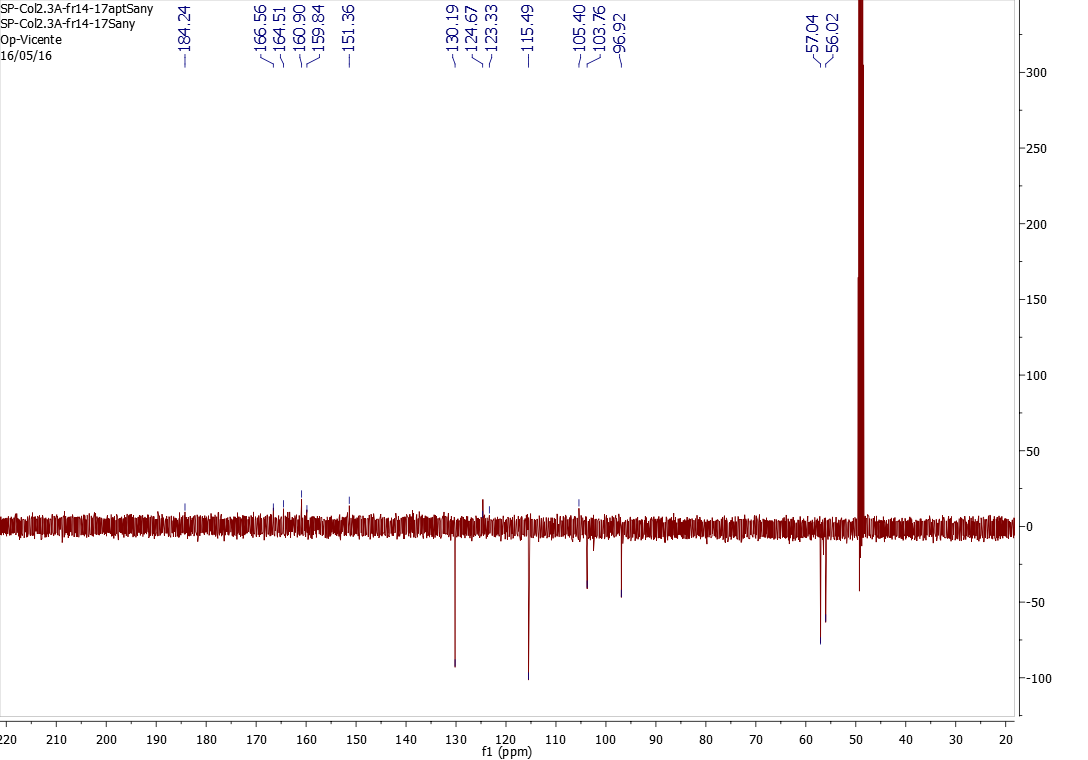


**Figure S29**. ^13^C NMR spectrum (125 MHz, CD_3_OD) of Compound **7**.


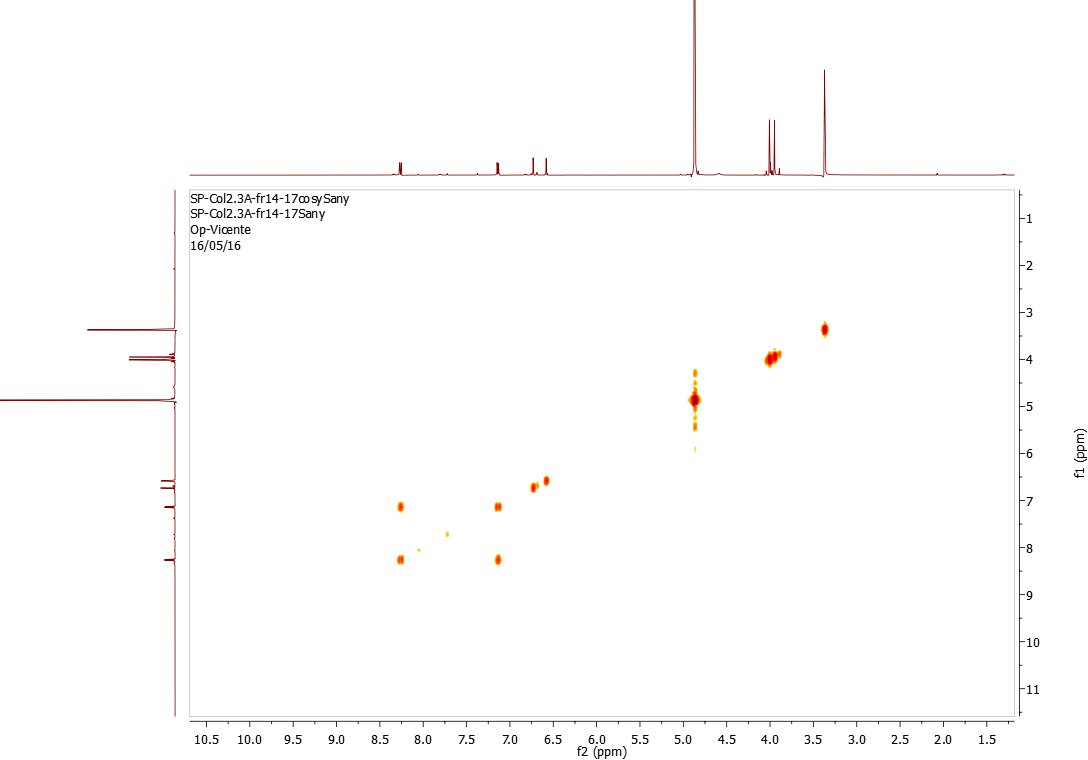


**Figure S30**. COSY spectrum (^1^H NMR: 500 MHz, CD_3_OD) of Compound **7**.


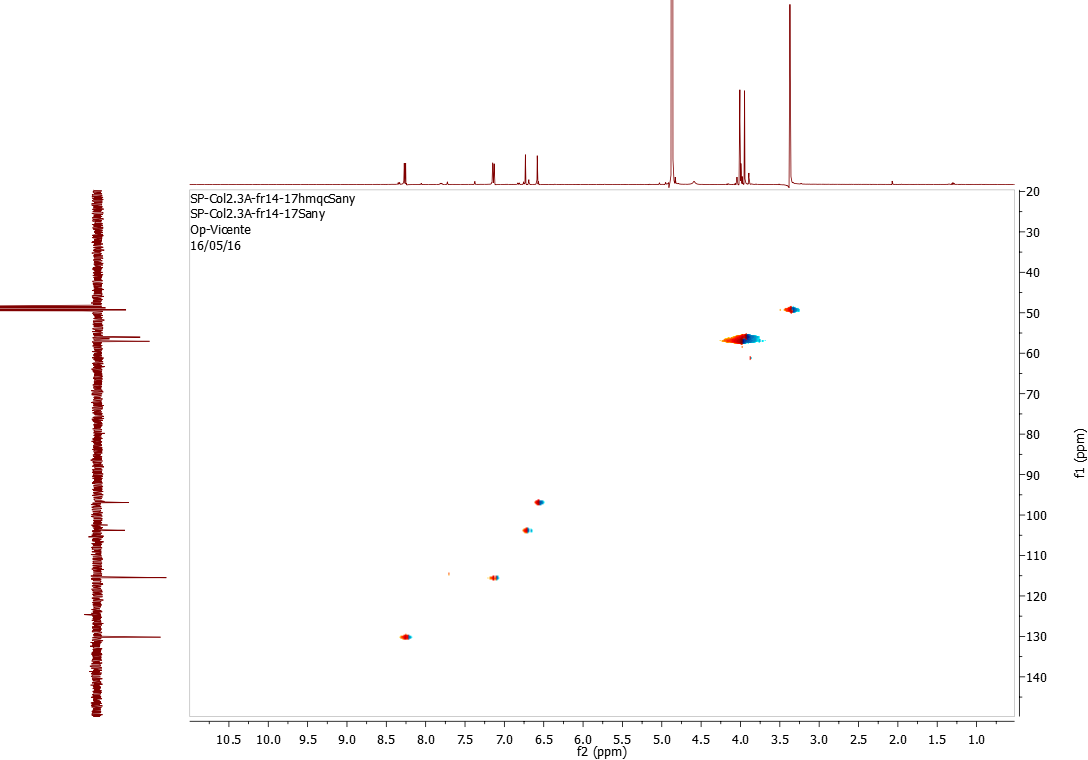


**Figure S31**. HMQC spectrum (^1^H-NMR: 500 MHz, ^13^C-NMR: 125 MHz, CD_3_OD) of Compound **7**.


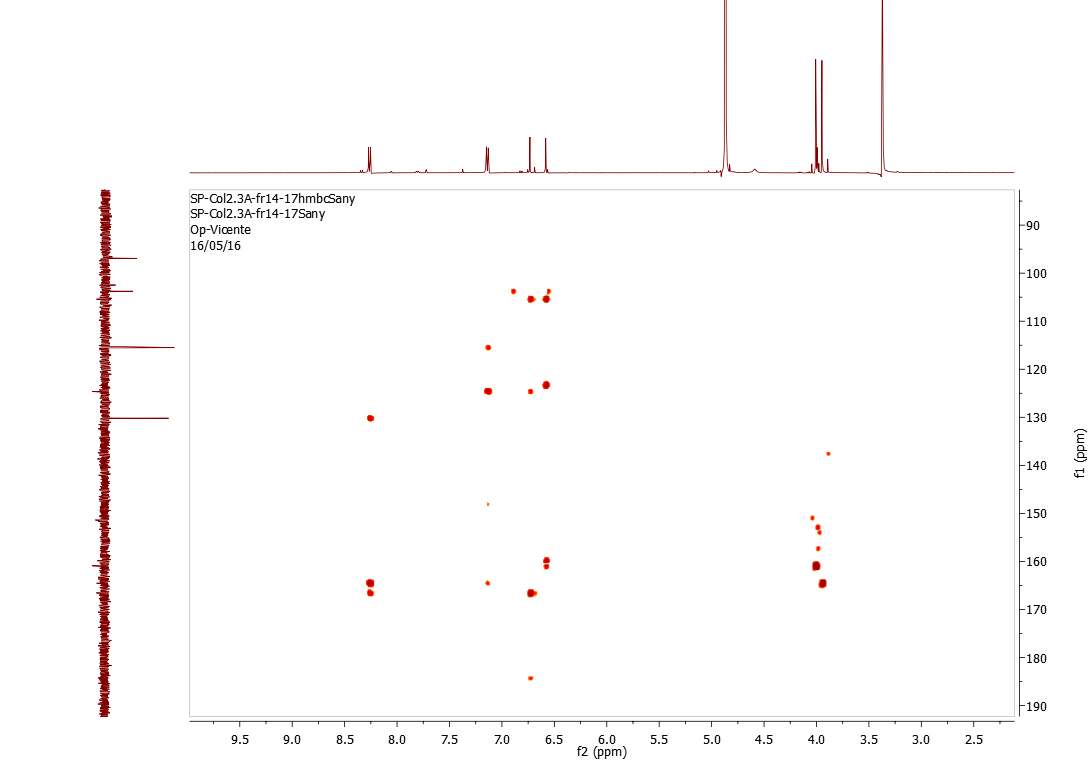


**Figure S32**. HMBC spectrum (^1^H-NMR: 500 MHz, ^13^C-NMR: 125 MHz, CD_3_OD) of Compound **7**.


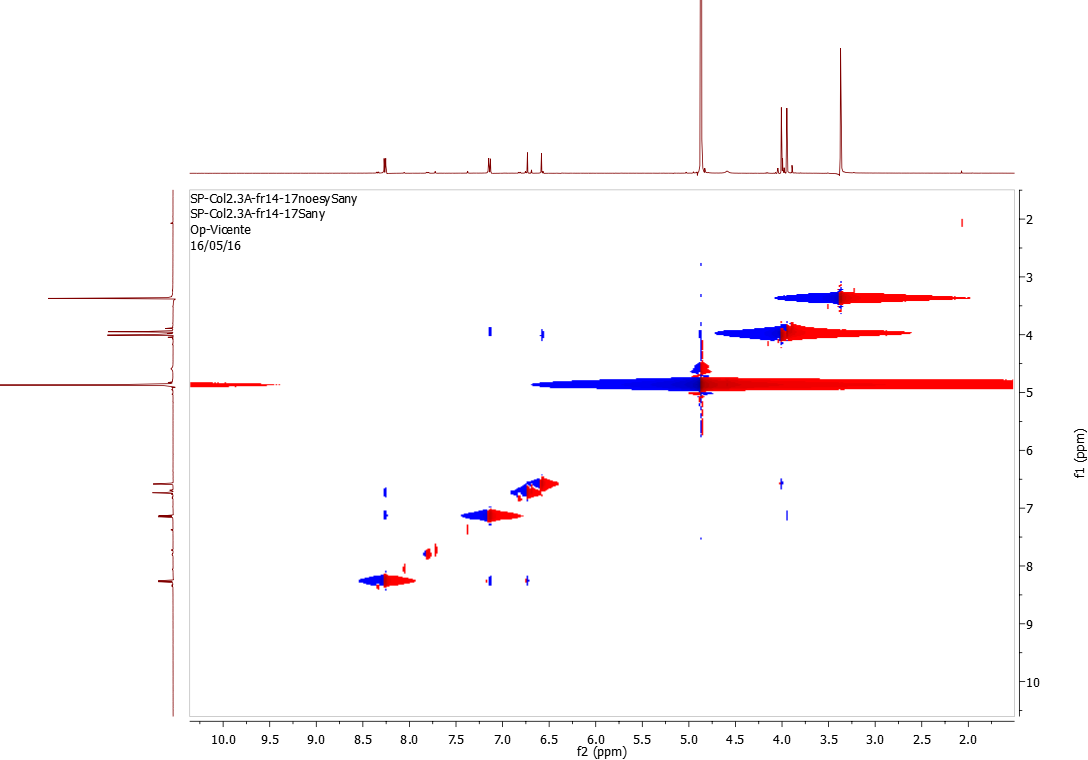


**Figure S33**. NOESY spectrum (^1^H-NMR: 500 MHz, CD_3_OD) of Compound **7**.


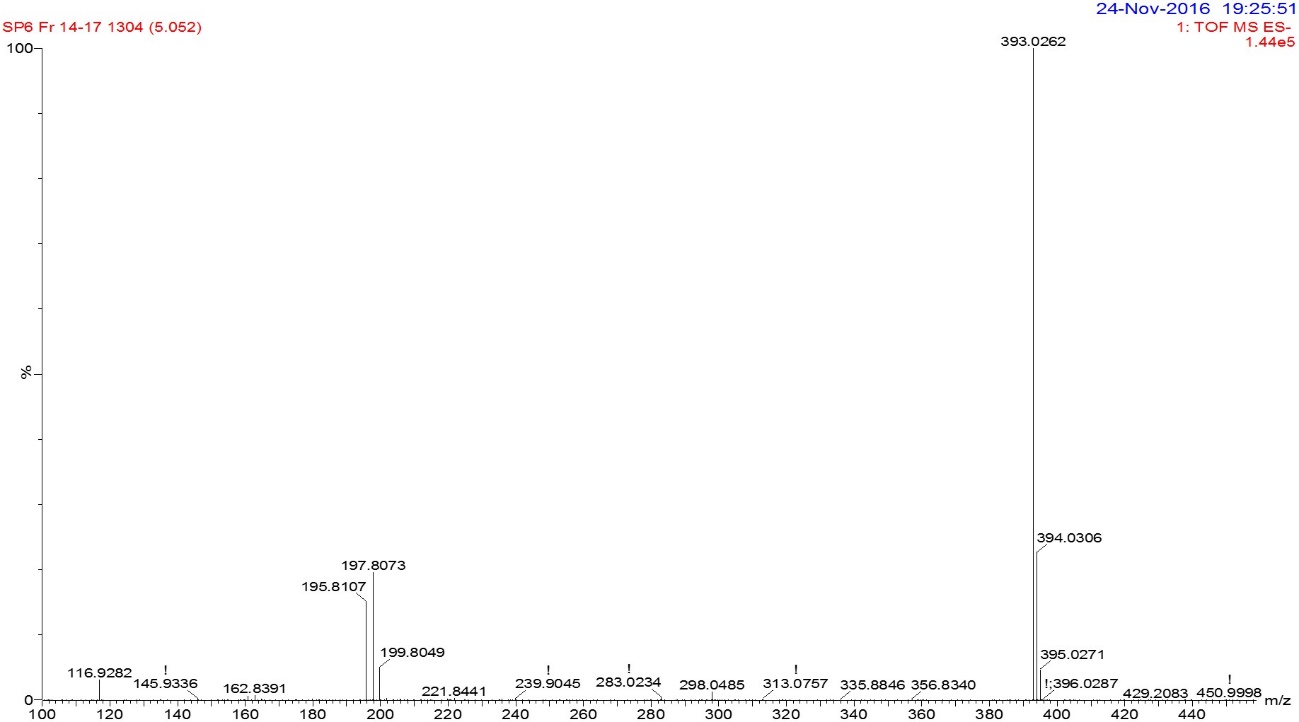


**Figure S34**. Mass spectrum of Compound **7**.


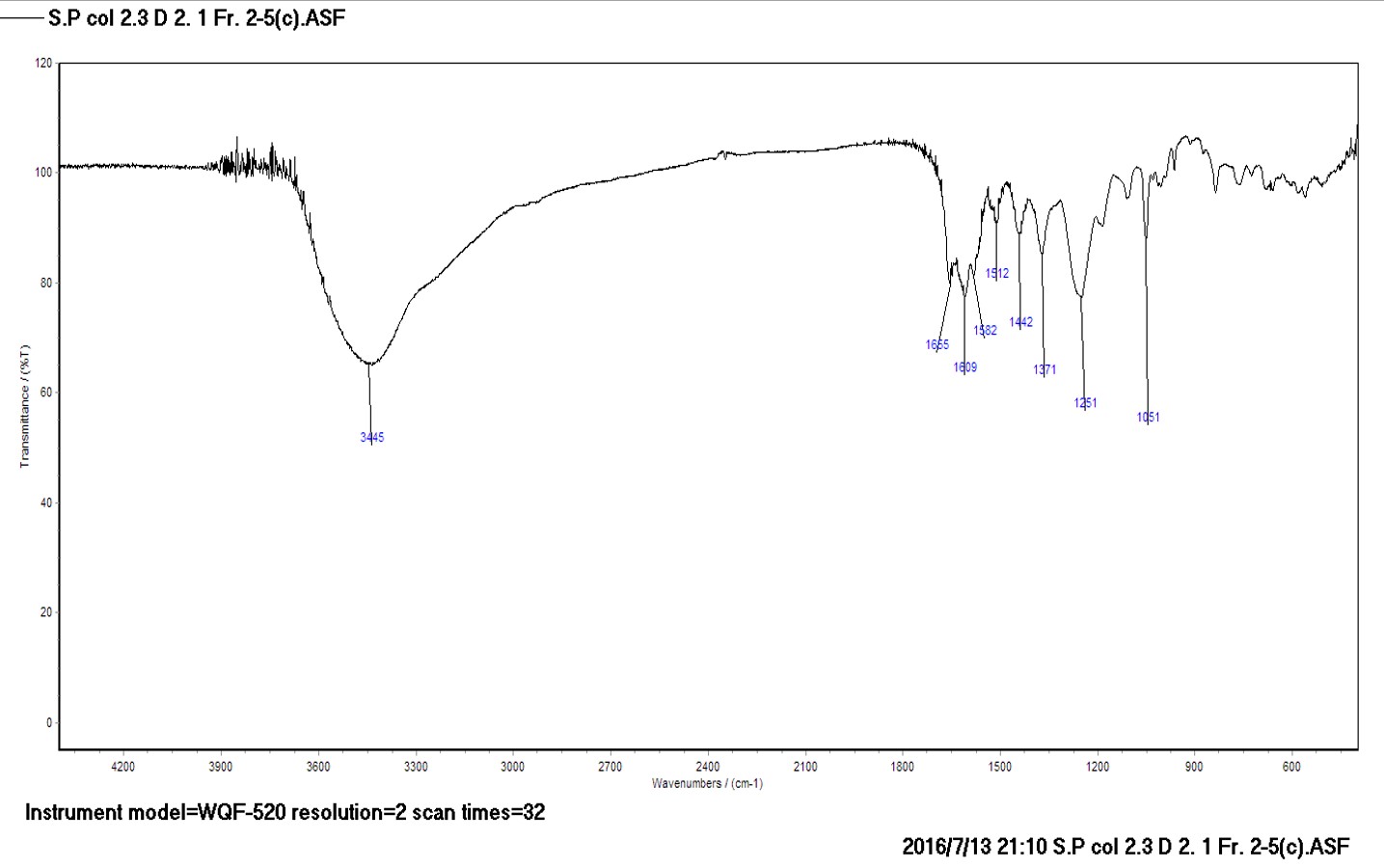


**Figure S35**. FTIR (KBr) spectrum of Compound **8**.


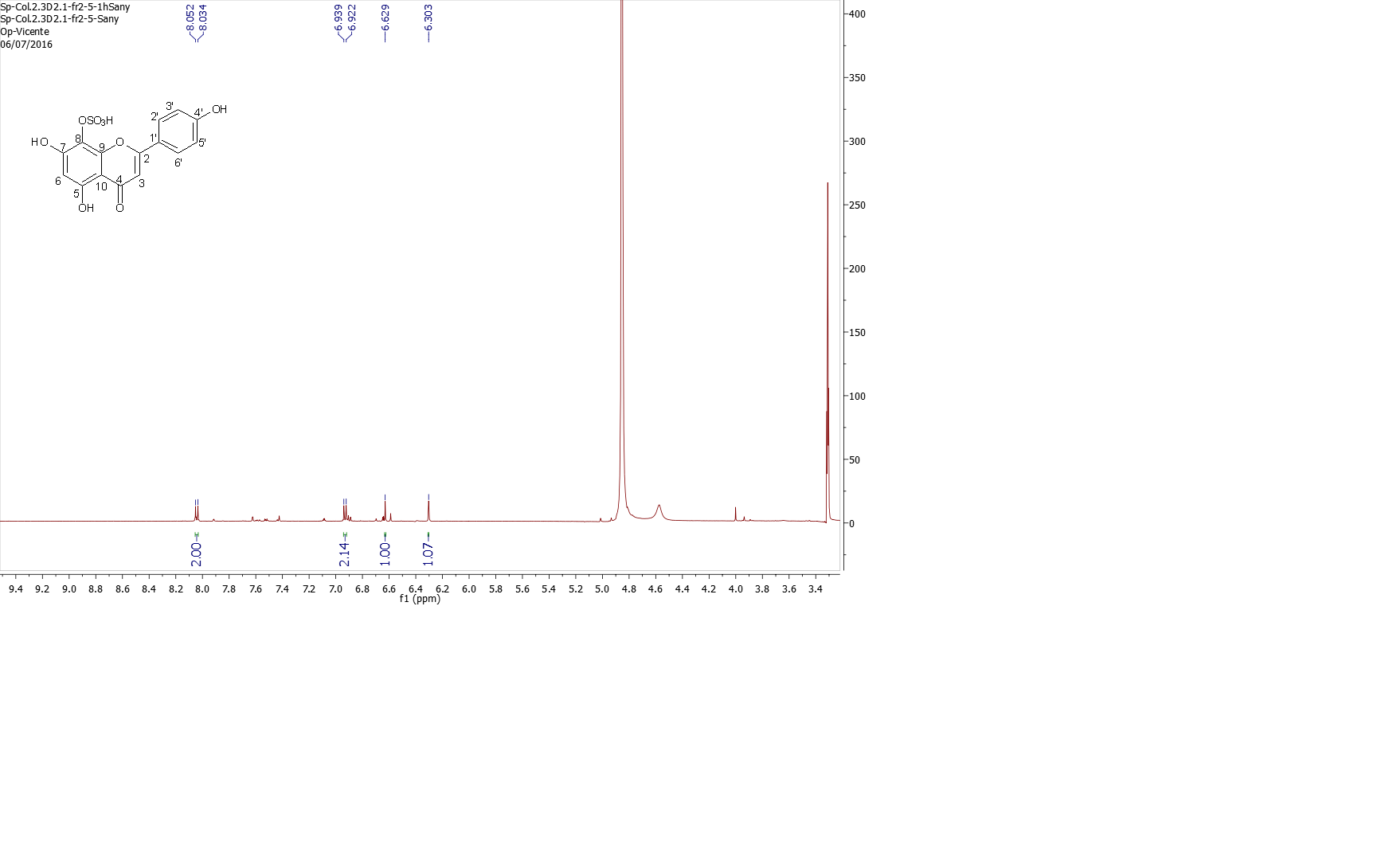


**Figure S36**. ^1^H NMR spectrum (500 MHz, CD_3_OD) of Compound **8**.


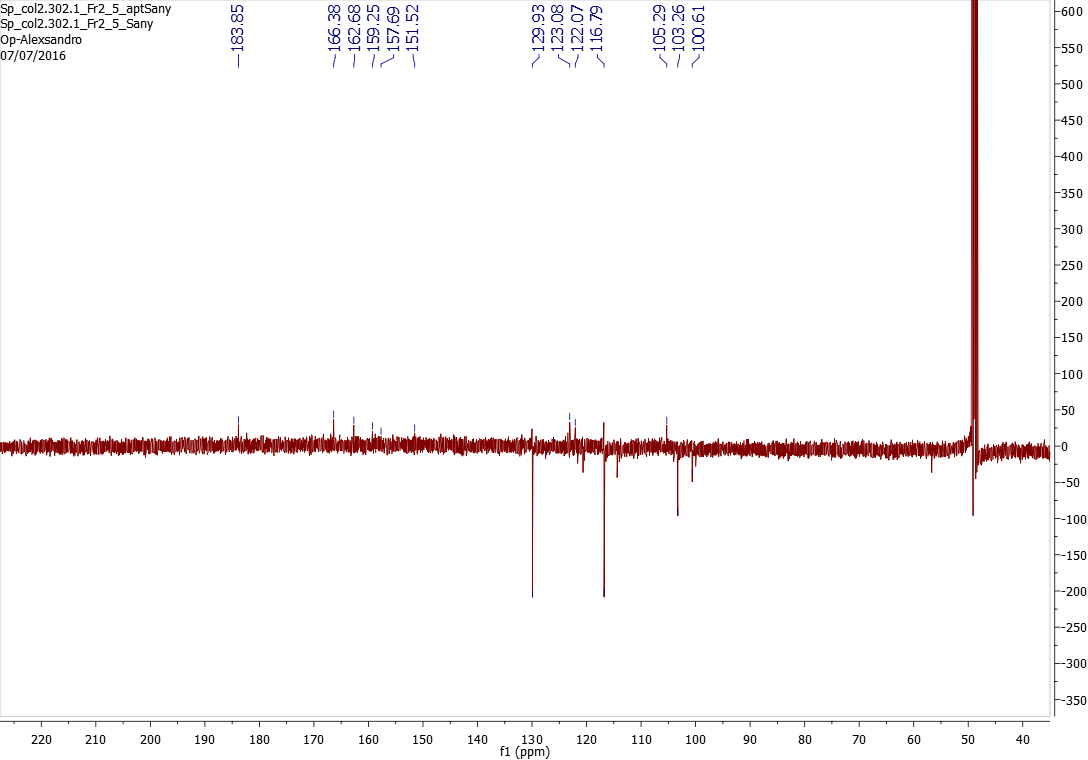


**Figure S37**. ^1^C NMR spectrum (125 MHz, CD_3_OD) of Compound **8**.


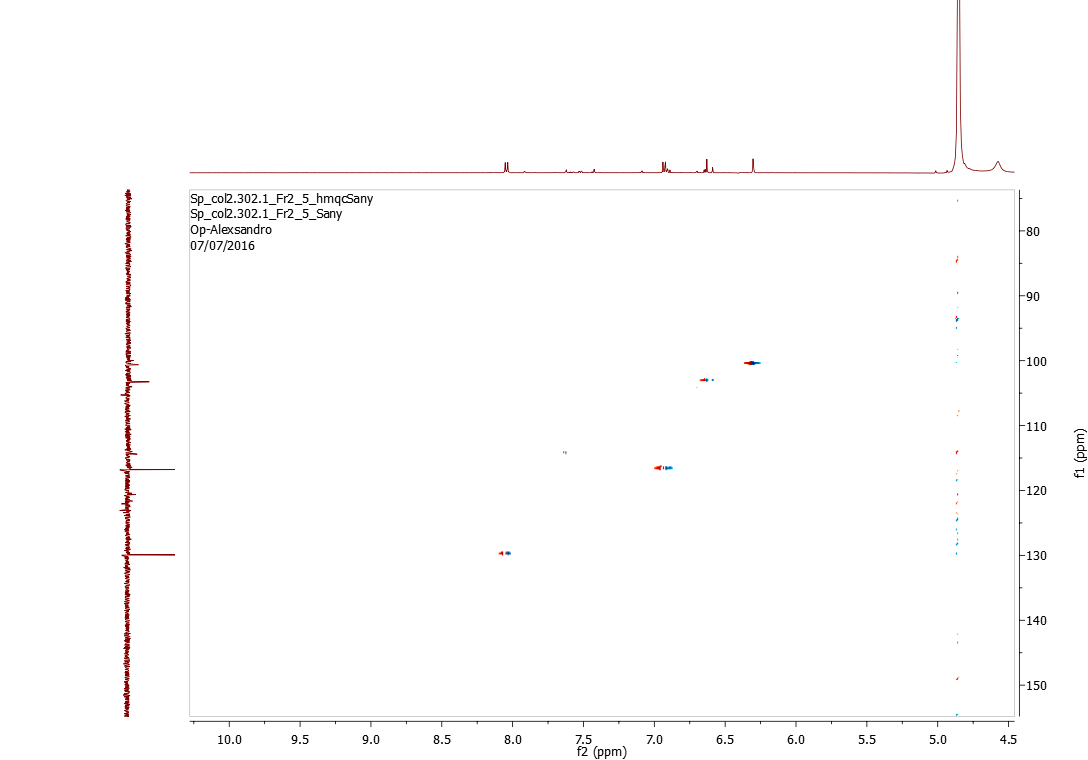


**Figure S38**. HMQC spectrum (^1^H-NMR: 500 MHz, ^13^C-NMR: 125 MHz, CD_3_OD) of Compound **8**.


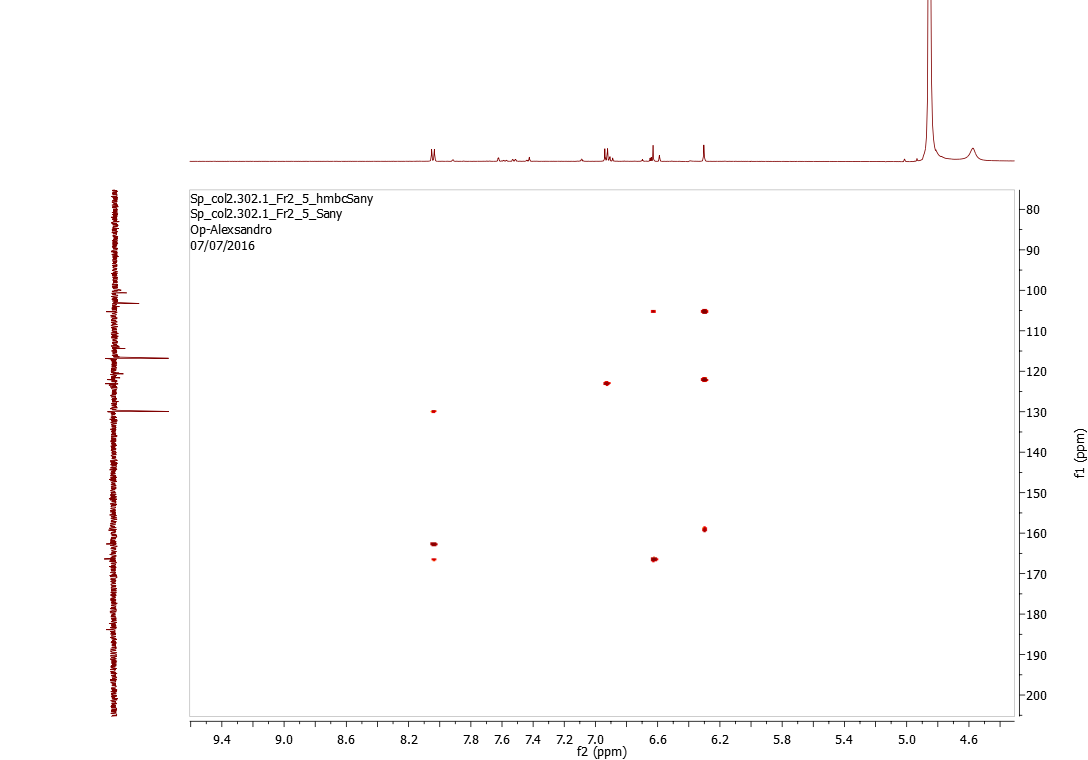


**Figure S39**. HMBC spectrum (^1^H-NMR: 500 MHz, ^13^C-NMR: 125 MHz, CD_3_OD) of Compound **8**.


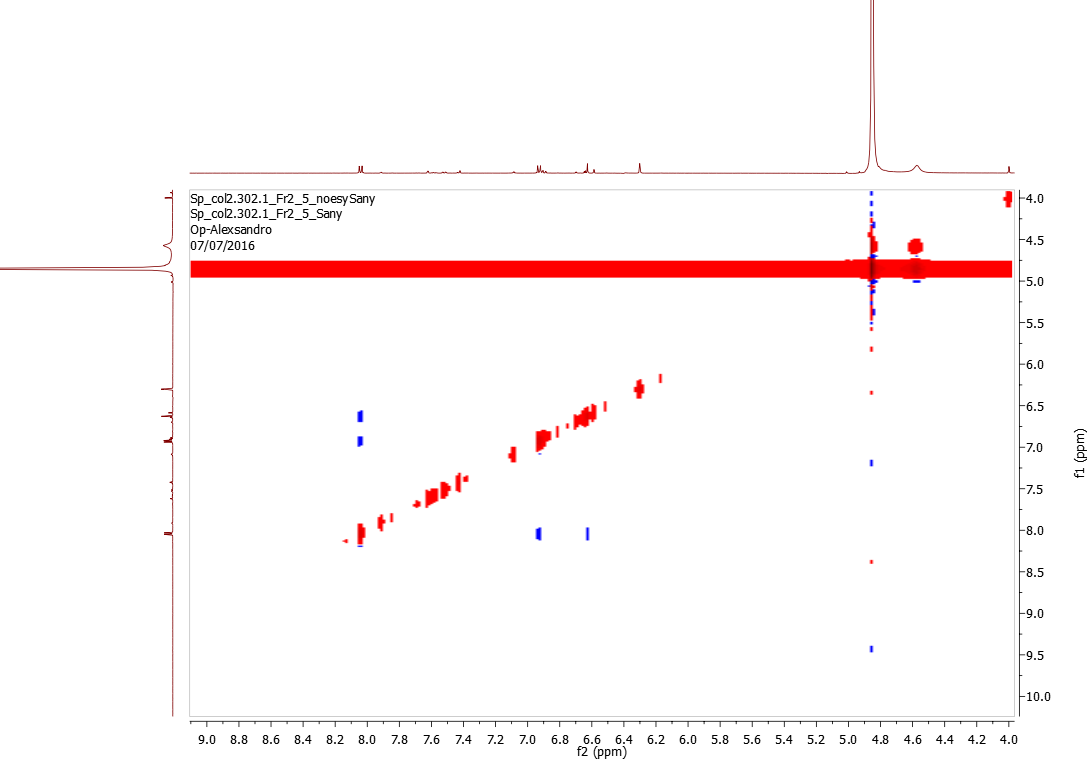


**Figure S40**. NOESY spectrum (^1^H-NMR: 500 MHz, CD_3_OD) of Compound **8**.


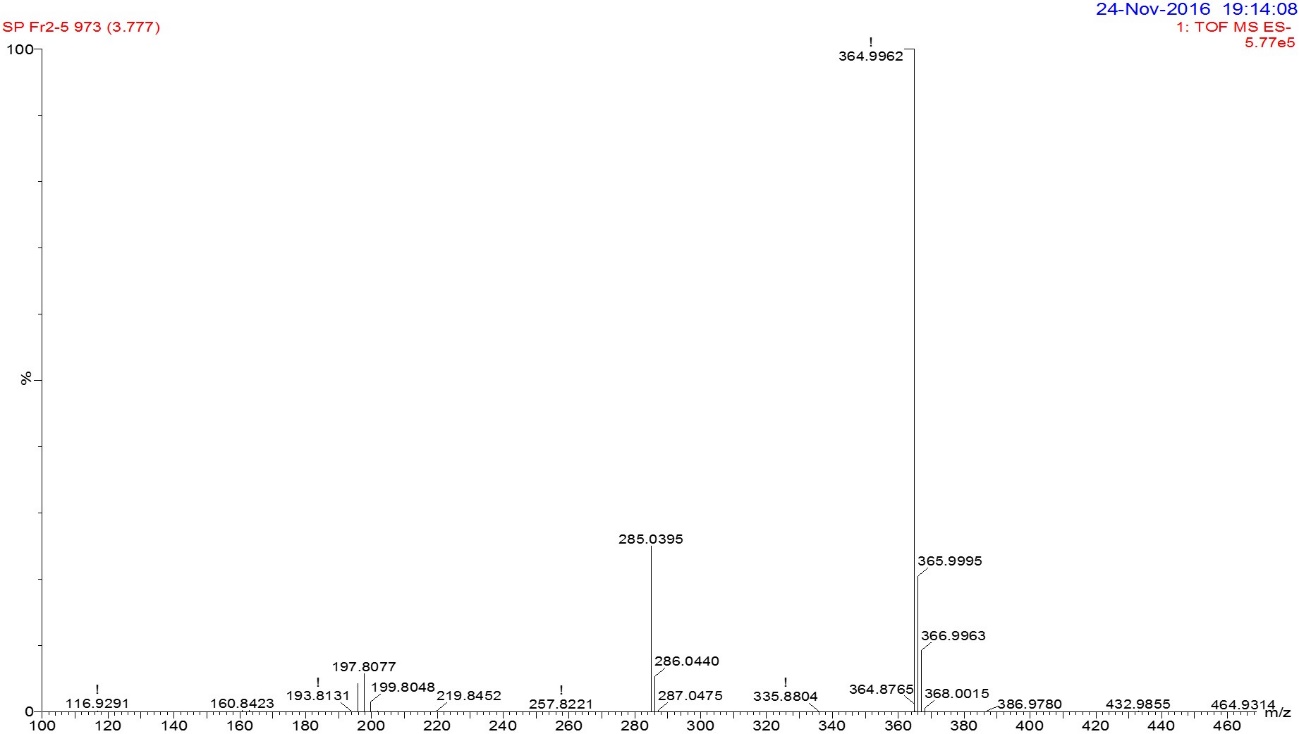


**Figure S41**. Mass spectrum of Compound **8**.


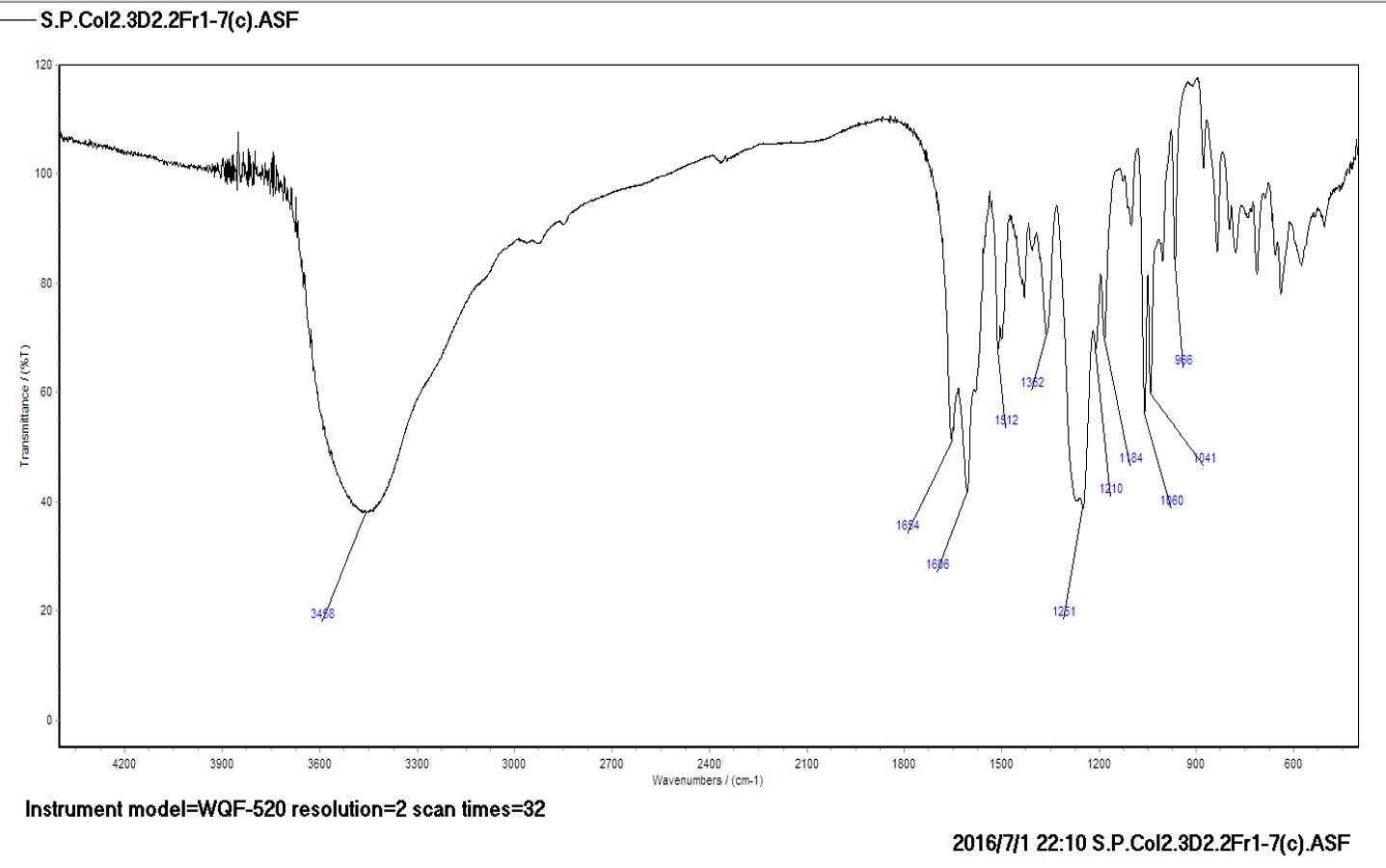


**Figure S42**. FTIR (KBr) spectrum of the Compounds **9a** and **9b**.


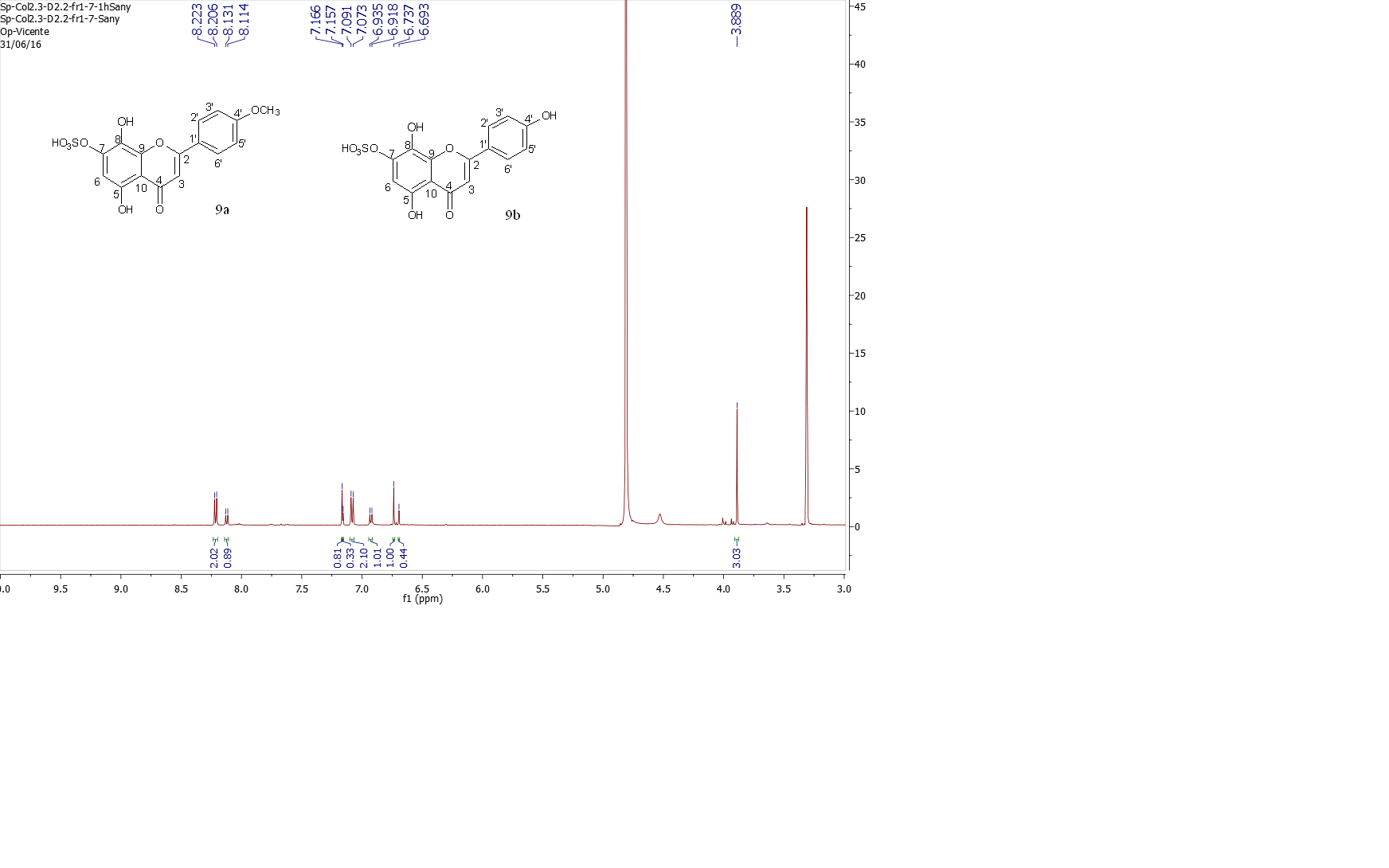


**Figure S43**. ^1^H NMR spectrum (500 MHz, CD_3_OD) of the Compounds **9a** and **9b**.


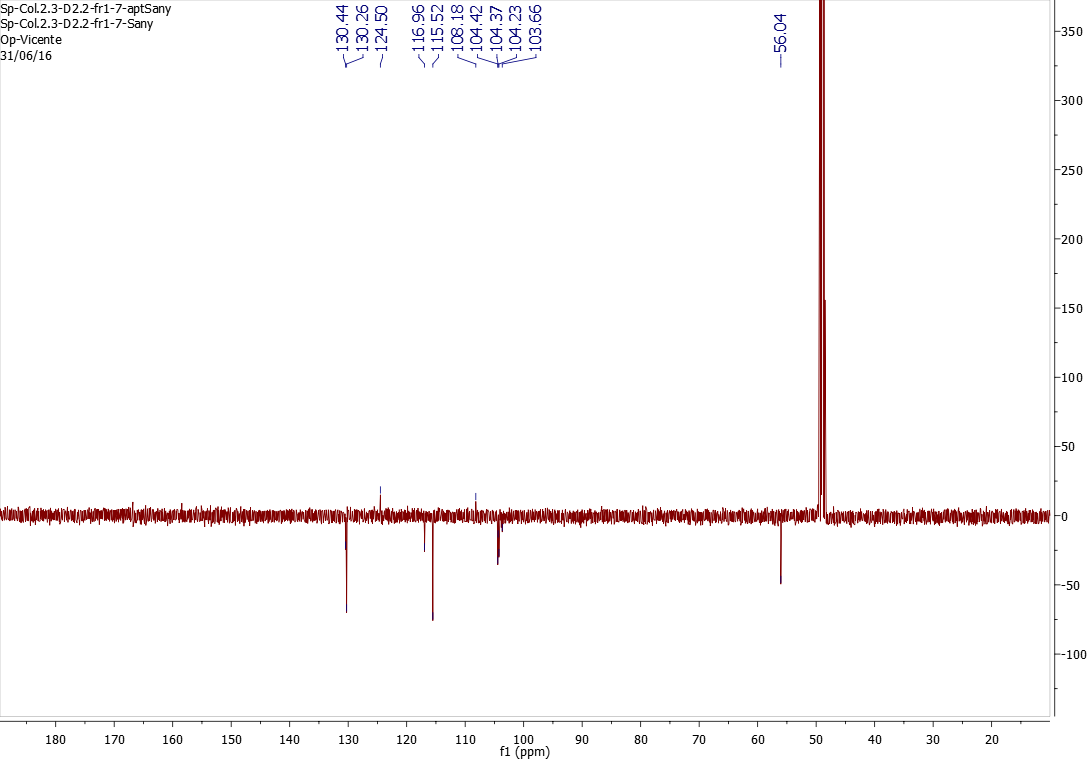


**Figure S44**. ^13^C NMR spectrum (125 MHz, CD_3_OD) of the Compounds **9a** and **9b**.


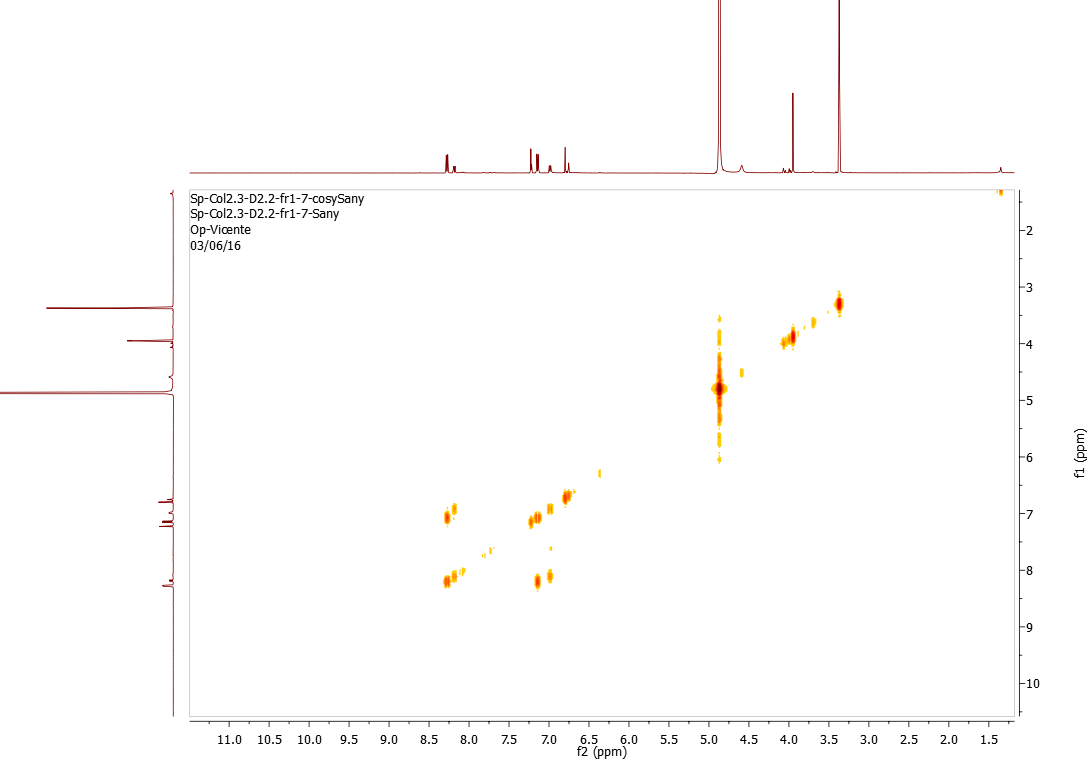


**Figure S45**. COSY spectrum (^1^H NMR: 500 MHz, CD_3_OD) of the Compounds **9a** and **9b**.


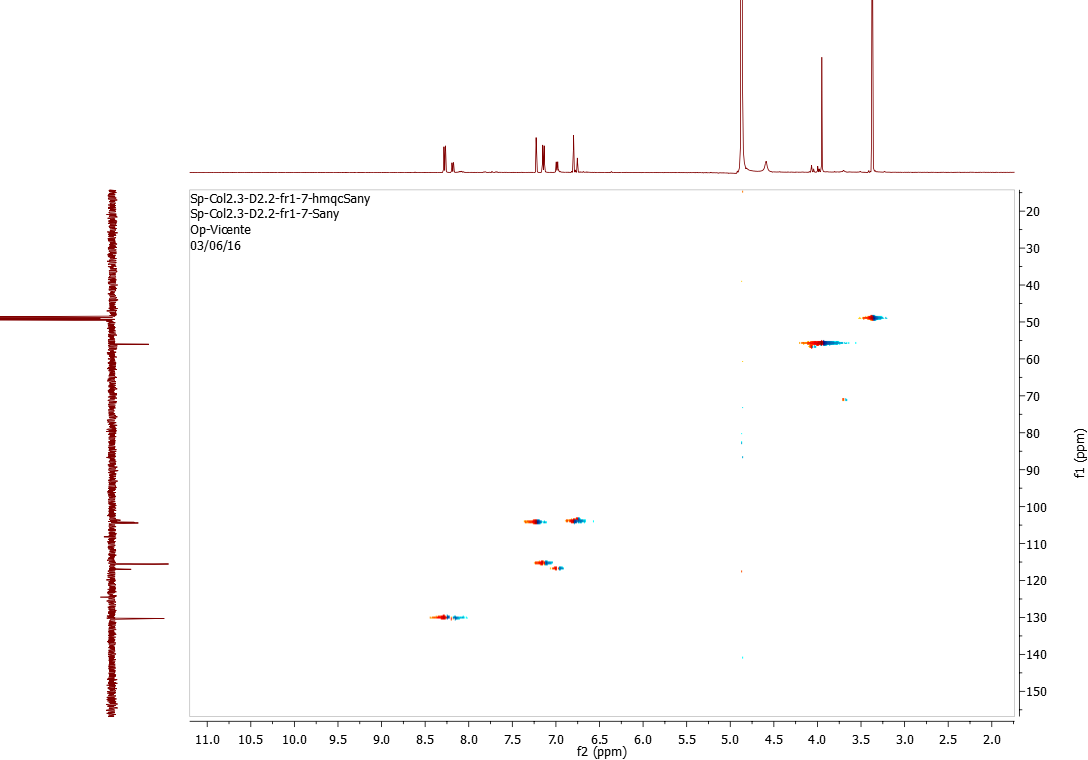


**Figure S46**. HMQC spectrum (^1^H-NMR: 500 MHz, ^13^C-NMR: 125 MHz, CD_3_OD) of the Compounds **9a** and **9b**.


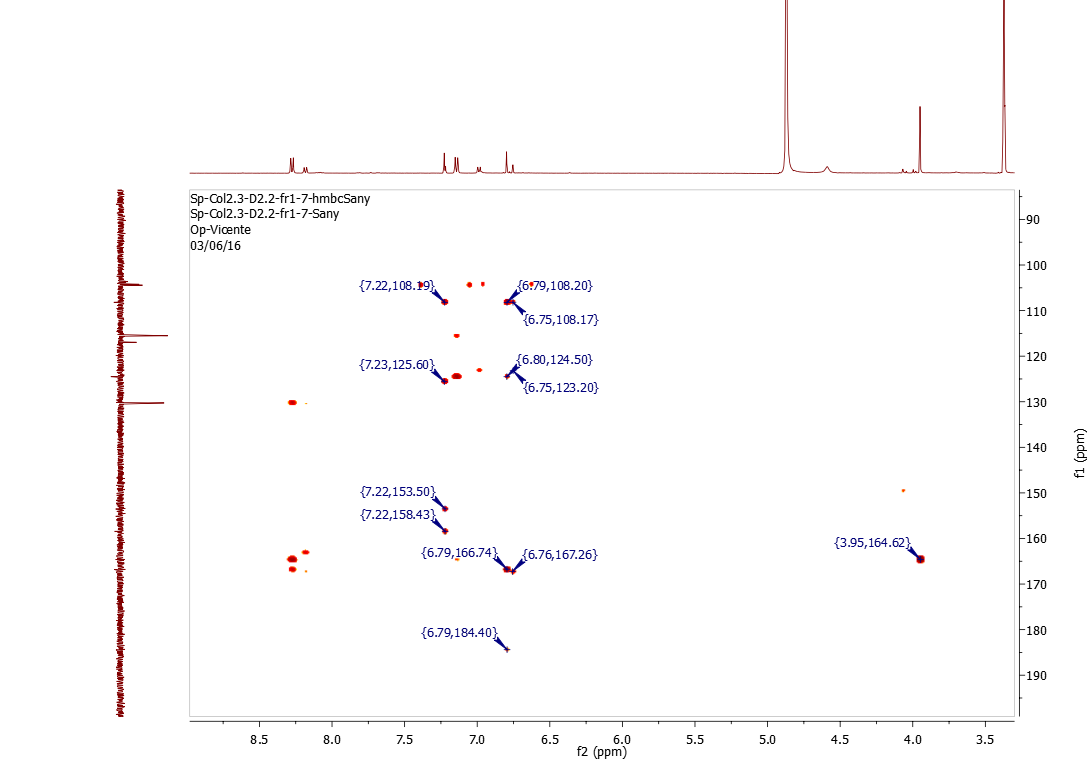


**Figure S47**. HMBC spectrum (^1^H-NMR: 500 MHz, ^13^C-NMR: 125 MHz, CD_3_OD) of the Compounds **9a** and **9b**.


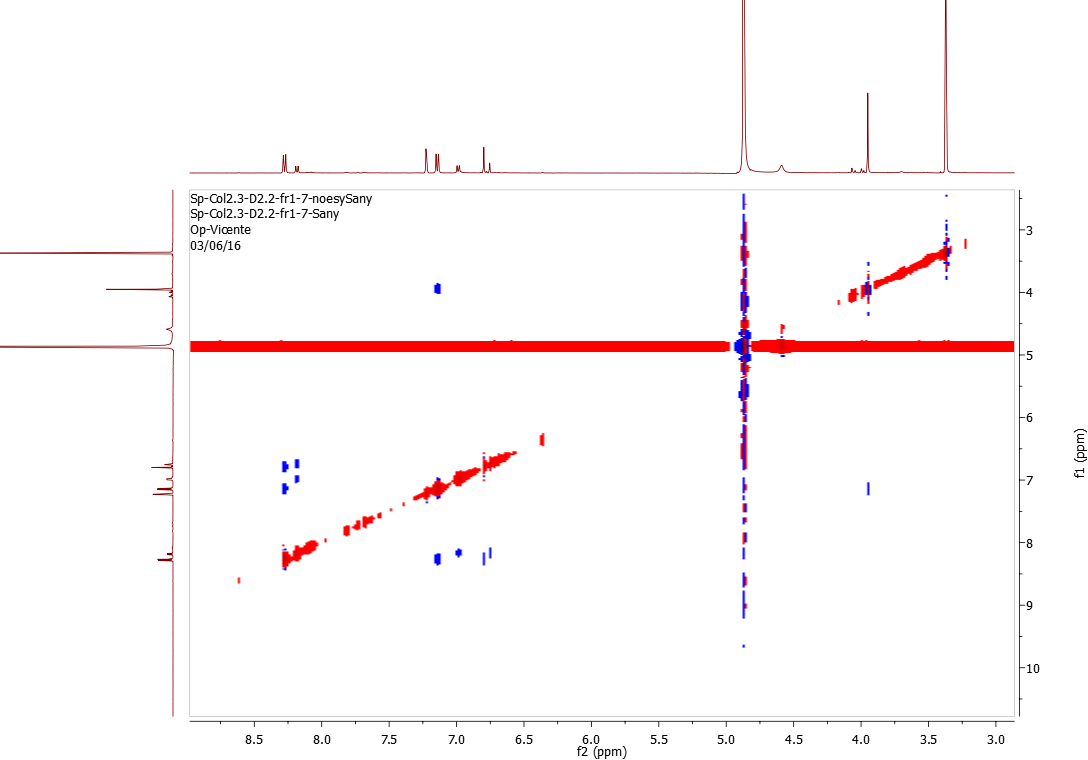


**Figure S48**. NOESY spectrum (^1^H-NMR: 500 MHz, CD_3_OD) of the Compounds **9a** and **9b**.


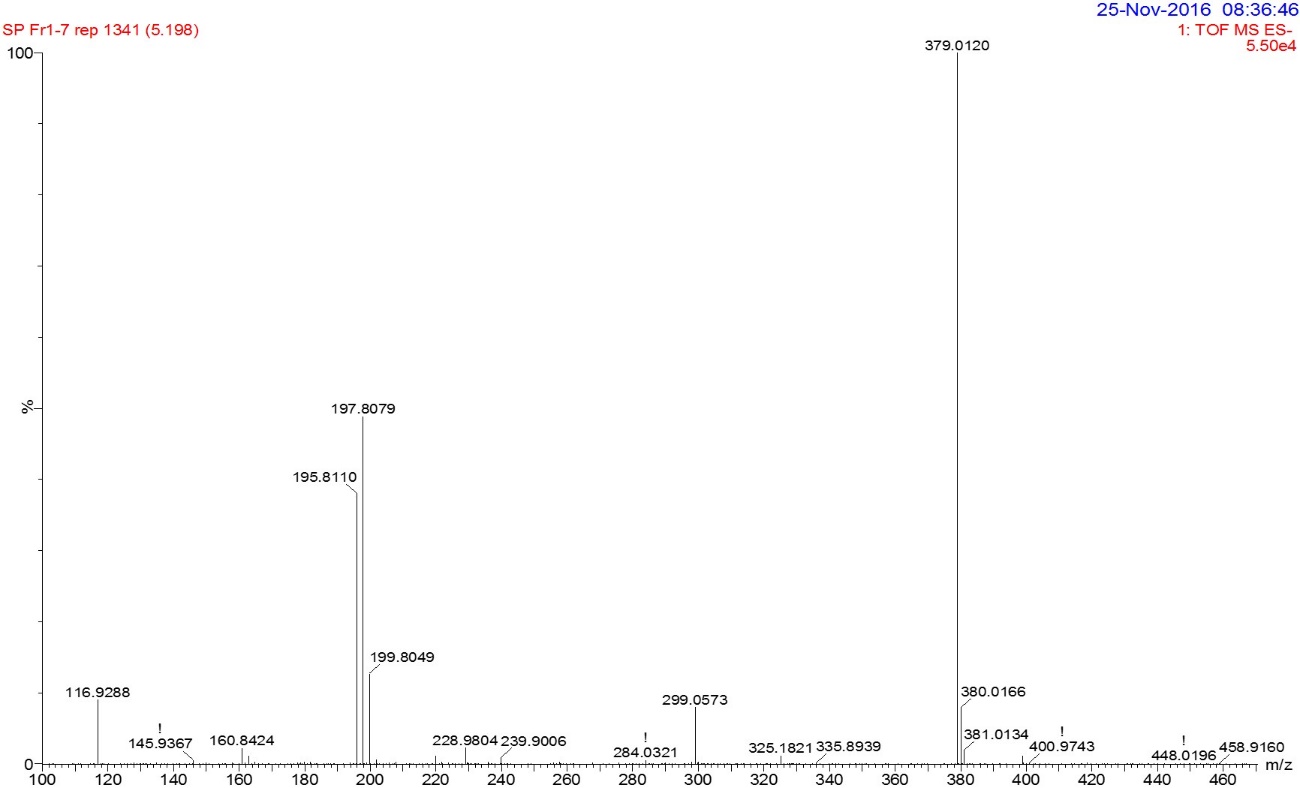


**Figure S49**. Mass spectrum of Compound **9a**.

**Figure S50**. Mass spectrum of Compound **9b**.


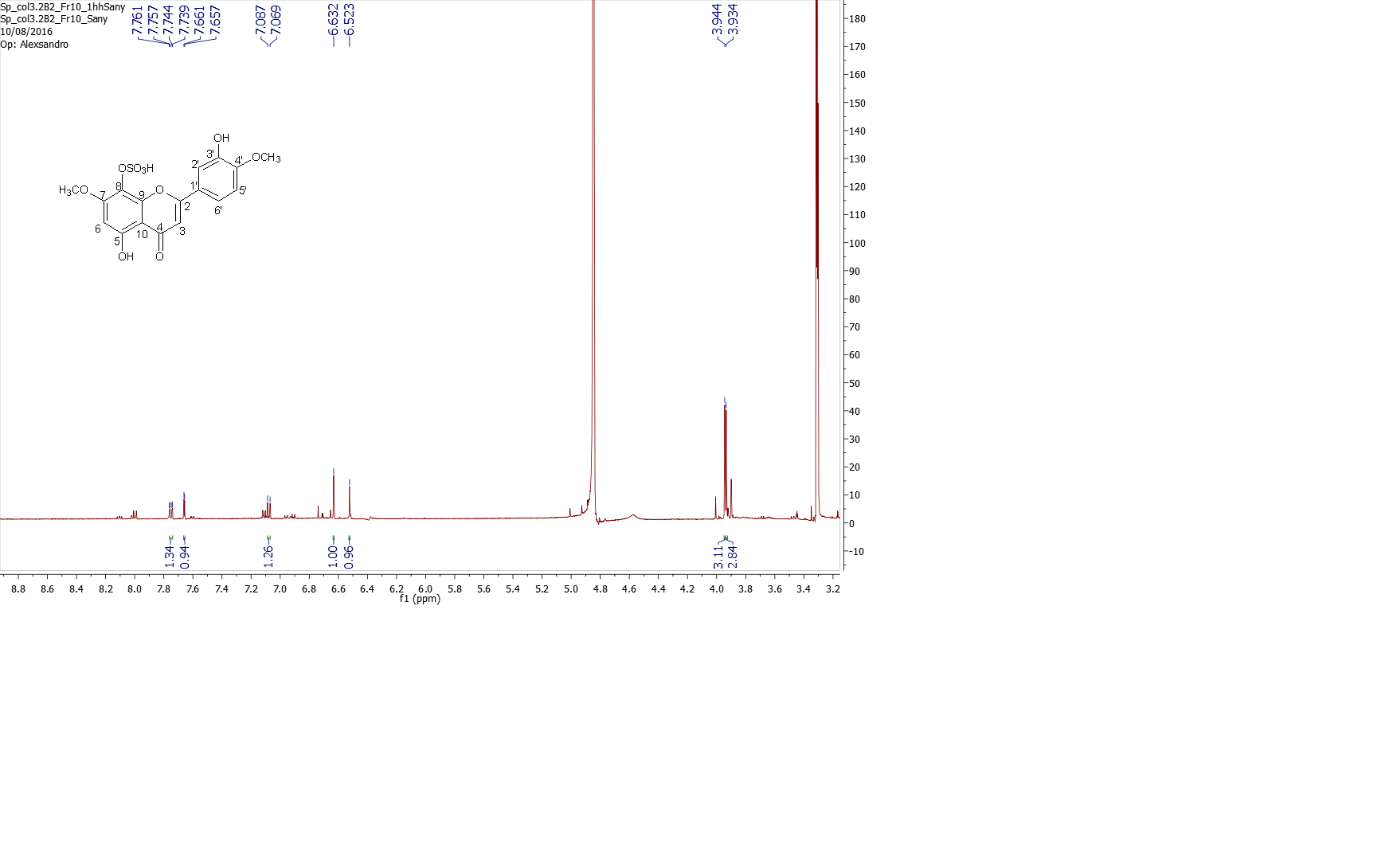


**Figure S51**. ^1^H NMR spectrum (500 MHz, CD_3_OD) of Compound **10**.


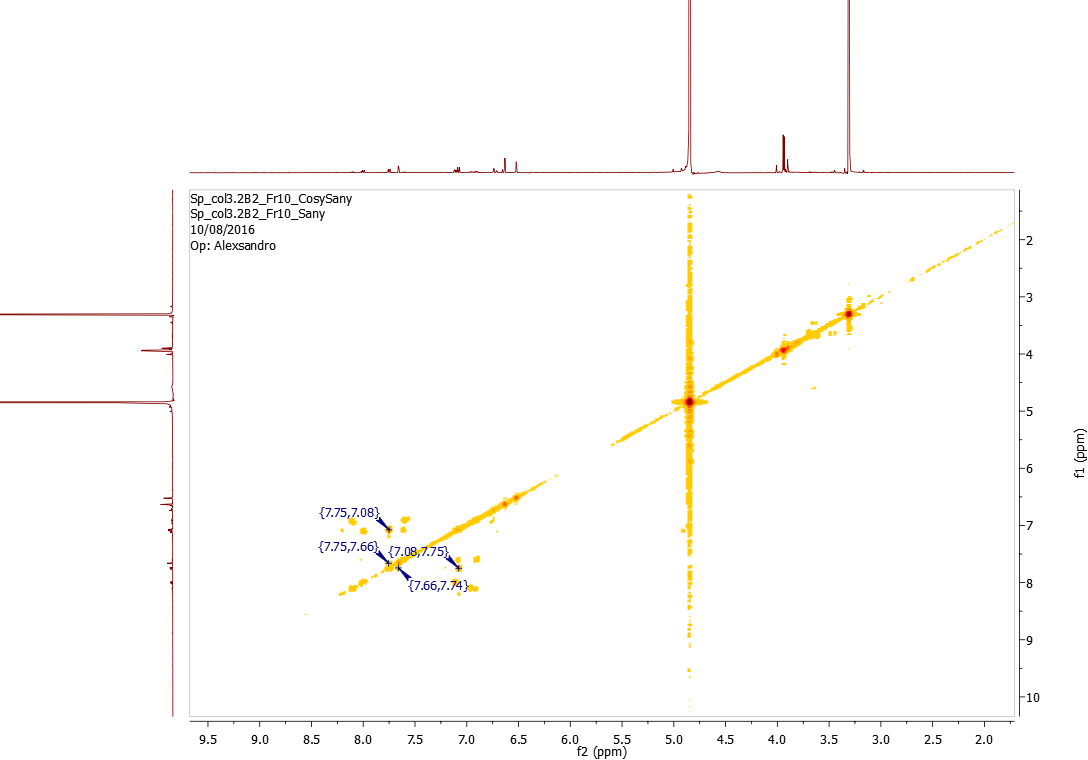


**Figure S52**. COSY spectrum (^1^H NMR: 500 MHz, CD_3_OD) of Compound **10**.


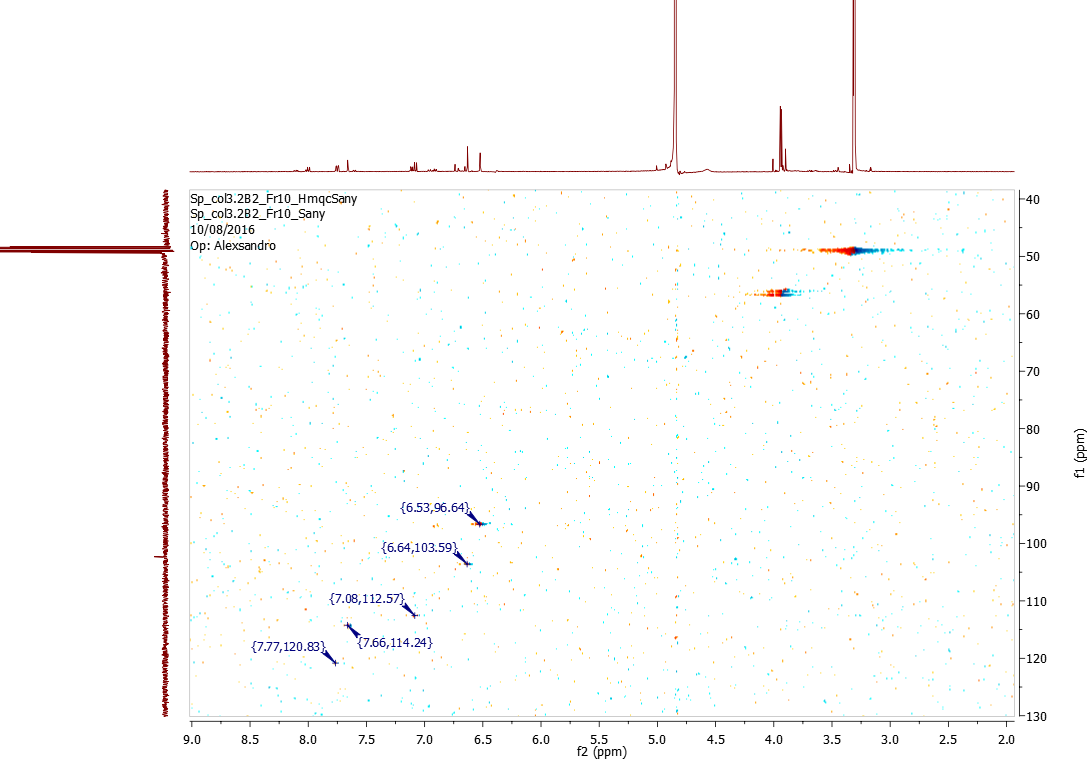


**Figure S53**. HMQC spectrum (^1^H-NMR: 500 MHz, ^13^C-NMR: 125 MHz, CD_3_OD) of Compound **10**.


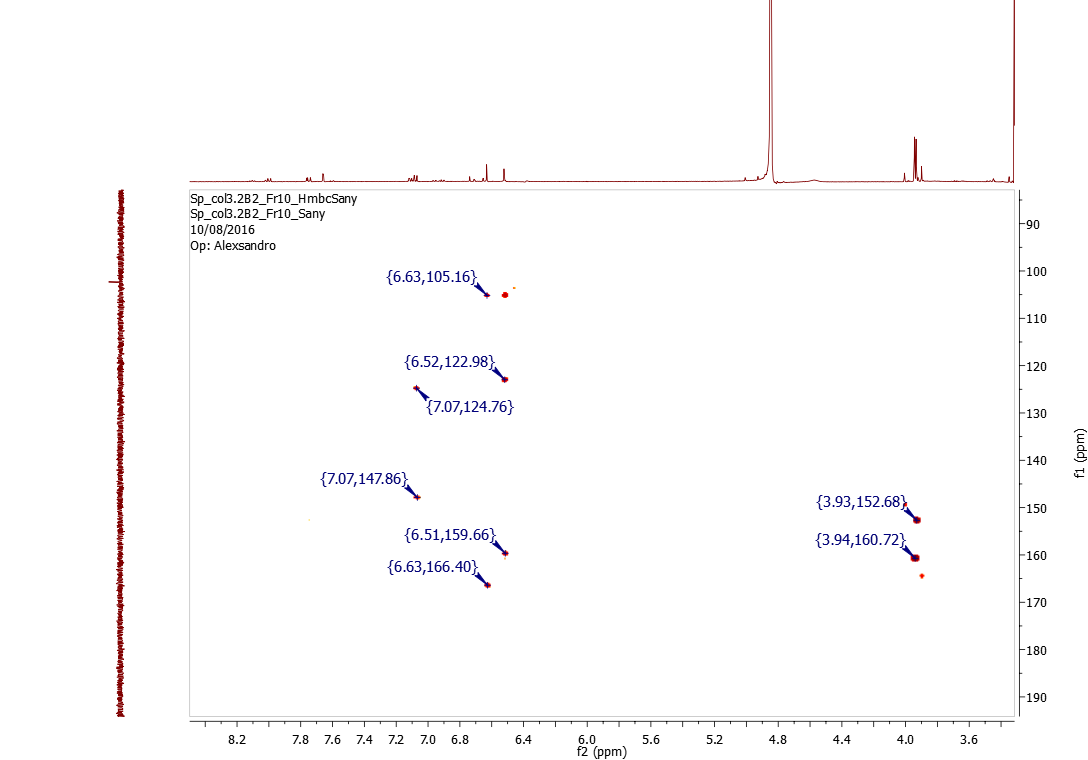


**Figure S54**. HMBC spectrum (^1^H-NMR: 500 MHz, ^13^C-NMR: 125 MHz, CD_3_OD) of Compound **10**.


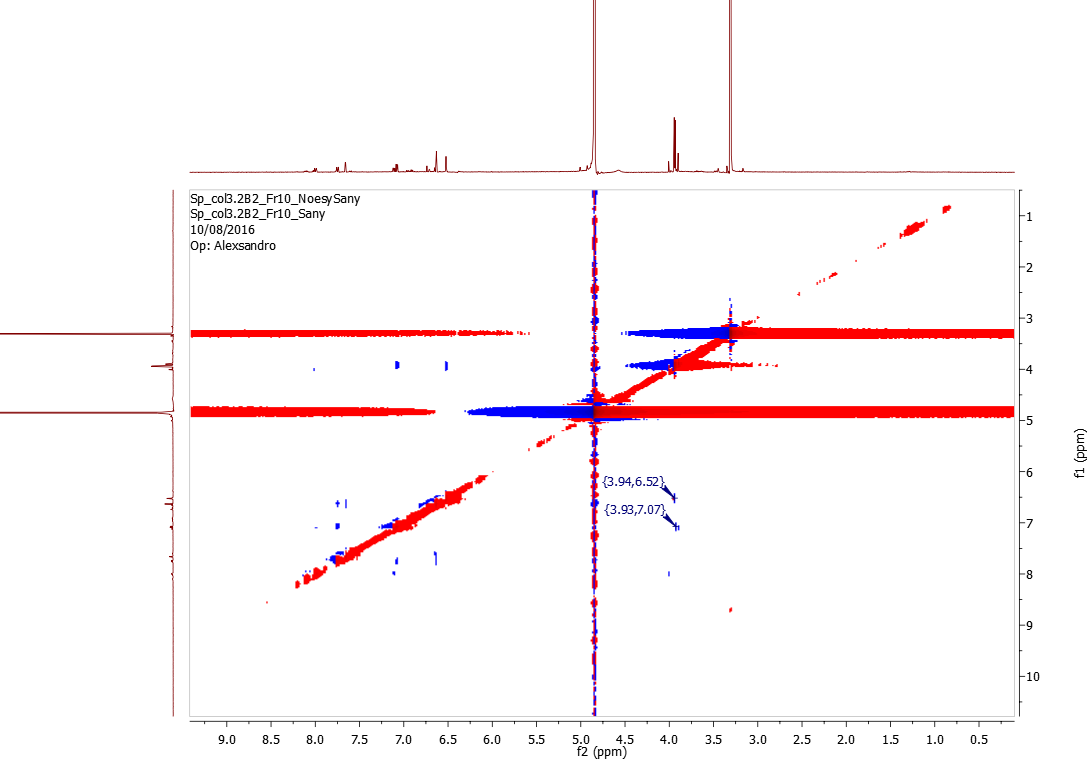


**Figure S55**. NOESY spectrum (^1^H-NMR: 500 MHz, CD_3_OD) of Compound **10**.


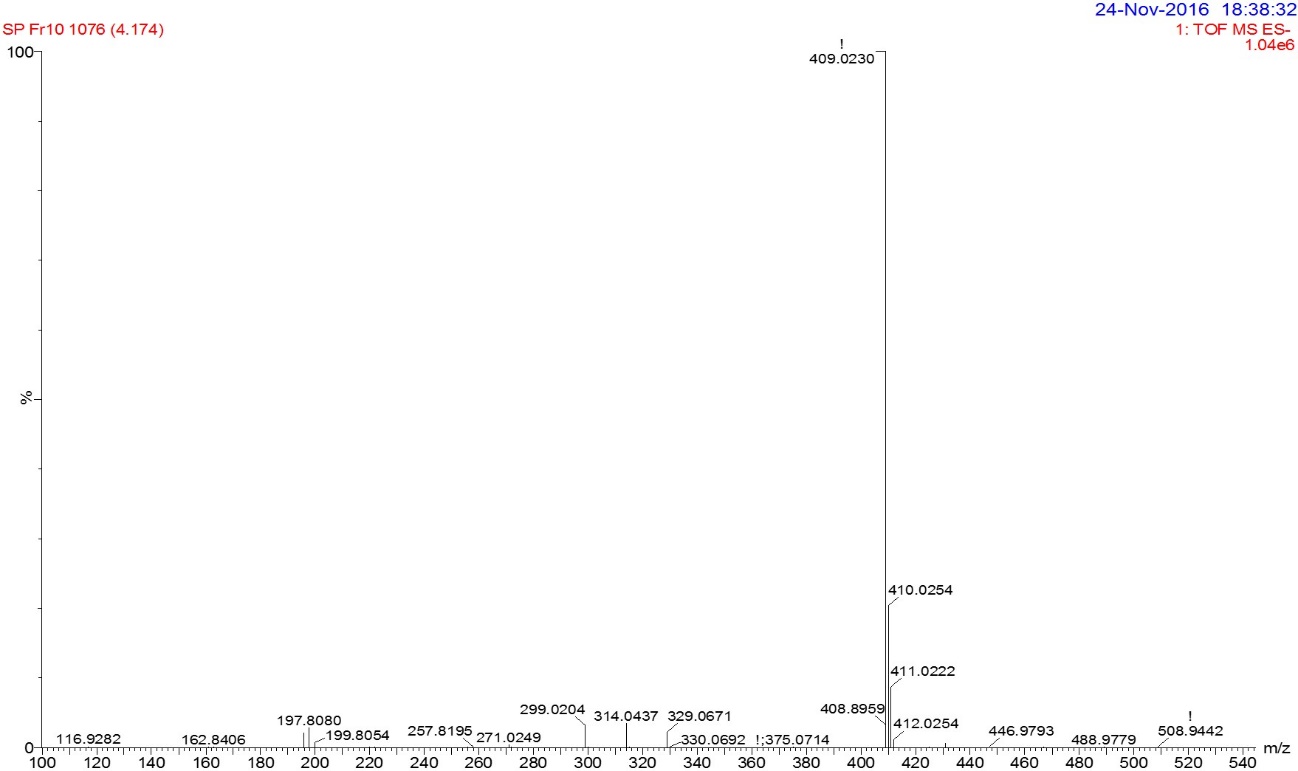


**Figure S56**. Mass spectrum of Compound **10**.
